# From β‑Dicarbonyl Chemistry to Dynamic Polymers

**DOI:** 10.1021/acs.chemrev.5c00307

**Published:** 2025-09-23

**Authors:** Youwei Ma, Christoph Weder, Filip E. Du Prez, José Augusto Berrocal

**Affiliations:** † Institute of Materials, 27218 École Polytechnique Fédérale de Lausanne (EPFL), Lausanne 1015, Switzerland; ‡ Adolphe Merkle Institute, 27211 University of Fribourg, Chemin des Verdiers 4, 1700 Fribourg, Switzerland; § NCCR Bio-inspired Materials, 27211 University of Fribourg, Chemin des Verdiers 4, 1700 Fribourg, Switzerland; ∥ Centre of Macromolecular Chemistry (CMaC), Department of Organic and Macromolecular Chemistry, Faculty of Sciences, 26656 Ghent University, 9000 Ghent, Belgium; ⊥ Institute of Chemical Research of Catalonia (ICIQ), Barcelona Institute of Science and Technology (BIST), Avda. Països Catalans, 16, Tarragona E-43007, Spain

## Abstract

The past two decades have witnessed an explosion of the use of dynamic bonds in polymer science. The β-dicarbonyl skeleton has emerged as a most versatile platform motif that has been utilized to synthesize a plethora of dynamic polymers that leverage either reversible metal–ligand coordination or exchangeable dynamic covalent bonds. The high modularity and intrinsic dynamic nature of the structures based on the β-dicarbonyl motif have received considerable interest across diverse fields, in applications that include drug delivery, the development of sustainable polymers, 3D printing, actuators, and many others. This review summarizes the progress on dynamic polymers derived from β-dicarbonyl synthons and focuses on three main topics. The first section provides a comprehensive overview of the prevalent methodologies employed for the preparation of polymers containing β-dicarbonyl moieties. The second part highlights the key features, development, and applications of dynamic polymers based on the β-dicarbonyl chemistry, including metallo-supramolecular polymers and dynamic covalent polymer networks. In the concluding section, we offer our views on the future challenges and prospects pertaining to this class of dynamic polymer systems.

## Graphical abstract

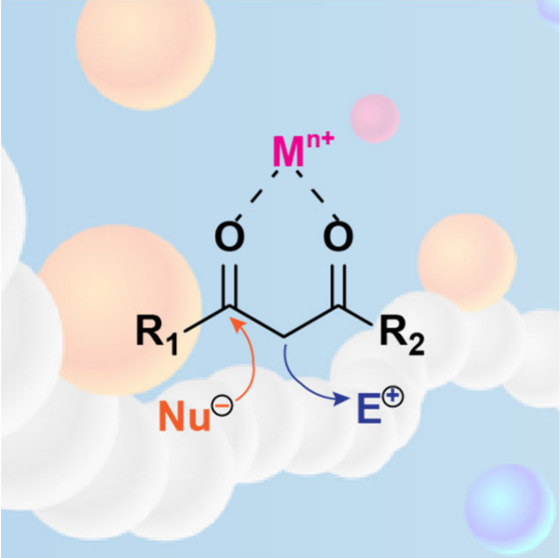

## 1 Introduction

Living creatures have developed unique and dynamic strategies of homeostasis and recognition that allow them to regenerate or adapt the structure or composition of chemical systems to support life.^1–3^ These strategies often involve the use of reversible, dynamic interactions of different strengths and energetic levels. Following these principles, researchers have integrated the concept of dynamic bonds (**DB**s) into synthetic macromolecular materials, referred to as dynamic polymers, which can display remarkable properties and functions, including high mechanical strength,^4,5^ self-healing,^6,7^ mechanochromic responses,^8–11^ and shape memory behavior,^12,13^ among others.^14–16^

Conceptually, any chemical interaction that can be reversibly broken and reformed can be considered a **DB**.^17,18^ This general definition resonates well with noncovalent interactions and dynamic covalent bonds (**DCB**s).^19^ Noncovalent interactions include metal–ligand coordination,^6,20–22^ hydrogen bonding,^23–27^ as well as host–guest,^28,29^ aromatic,^30,31^ and hydrophobic interactions.^32–34^ These noncovalent chemical linkages are inherently dynamic due to their kinetic lability and sensitivity to external stimuli, such as changes in temperature, irradiation with light of a specific wavelength, or mechanical forces (Figure 1a). The stimuli–responsiveness also allows for easy tuning of the mechanical, viscoelastic, and processing properties of polymer materials leveraging noncovalent interactions. However, such high modularity often comes at the expense of the limited chemical and thermomechanical robustness of polymers assembled solely via noncovalent interactions.

**Figure 1:**
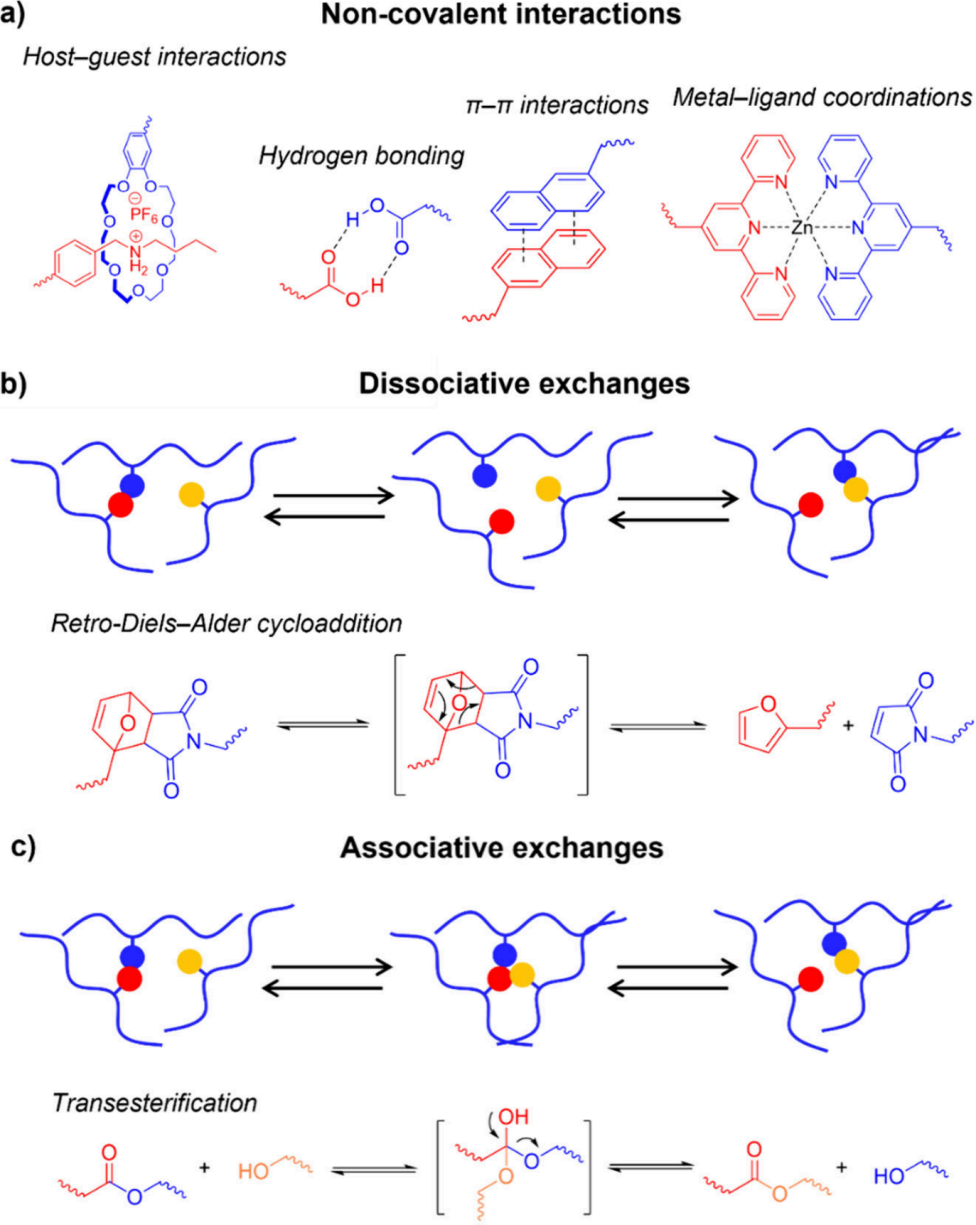
(a) Illustration of dynamic noncovalent interactions between selected chemical moieties. Examples of **DCB**s undergoing (b) dissociative exchange and (c) associative exchange.

Being based on reversible, *covalent* linkages, **DCB**s generally possess higher strength and are more chemically stable than noncovalent interactions, which should overcome (some of) the limitations previously discussed for polymers leveraging noncovalent interactions. One of the main consequences of the higher strength and stability of **DCB**s is the higher energy that is required to trigger the dynamic process, i.e., the chemical exchange among chemical functionalities that constitute the **DCB**.^35^ Such exchange can occur through either dissociative (Figure 1b) or associative pathways (Figure 1c).^36–39^ In dissociative processes, **DCB**s break prior to the formation of new bonds, whereas bond breaking and reformation of **DCB**s occur simultaneously in the associative pathway. When considering dynamic covalent networks (**DCN**s), particularly the polymer networks with **DCB**s incorporated as cross-links, these two mechanisms have important consequences for the density of cross-links under exchange conditions. Indeed, dissociative **DCN**s undergo a decrease in the cross-link density, while associative **DCN**s retain the original density of cross-links. This difference results in a sharp decrease in viscosity for dissociative **DCN**s, whereas a more gradual decrease in viscosity is displayed by associative **DCN**s upon heating.^18,40^ Examples of dissociative dynamic covalent reactions include retro-Diels–Alder cycloadditions,^41,42^ oxime–urethane dissociation,^43,44^ boronic ester hydrolysis,^45^ and nitroxide radical exchanges^46,47^ (Figure 1b). Associative exchange reactions comprise transesterification,^48–50^ which formed the basis of the first report on associative **DCNs** by the Leibler lab in 2011,^51^ transamination of vinylogous urethanes,^52,53^ diketoenamines,^54^ and imines,^55–57^ as well as silyl ether exchange (Figure 1c).^58,59^ Associative **DCN**s were initially termed “vitrimers” due to their Arrhenius-like behavior of the rate of dynamic exchange, which is reminiscent of that of silica (lat. *vitrum*).^51^ However, recent studies have found that such Arrhenius-like dependence is more universal and applicable to both associative and some dissociative **DCN**s.^36,60^

β-Dicarbonyl skeletons — which feature a nucleophilic methylene group positioned between two electrophilic carbonyls — represent essential synthons in organic synthesis and powerful ligands in coordination chemistry.^61–63^ These motifs have been popular in materials science and biomedical analysis.^62,64,65^ Since the seminal work on the synthesis of vinylogous urethanes from β-ketoesters and amines reported by the Du Prez lab,^52^ β-dicarbonyl skeletons have also attracted significant interest in the context of dynamic polymer networks. The rapid emergence of β-dicarbonyl-based compounds in this domain can be partially attributed to the high synthetic accessibility and unique reactivity of these motifs, which promotes their high chemical versatility. The close proximity between two carbonyl groups strongly favors the prototropic tautomerism between the β-dicarbonyl and the enol form toward the latter, as it enables an intramolecular hydrogen bonding interaction (1, Figure 2).

**Figure 2:**
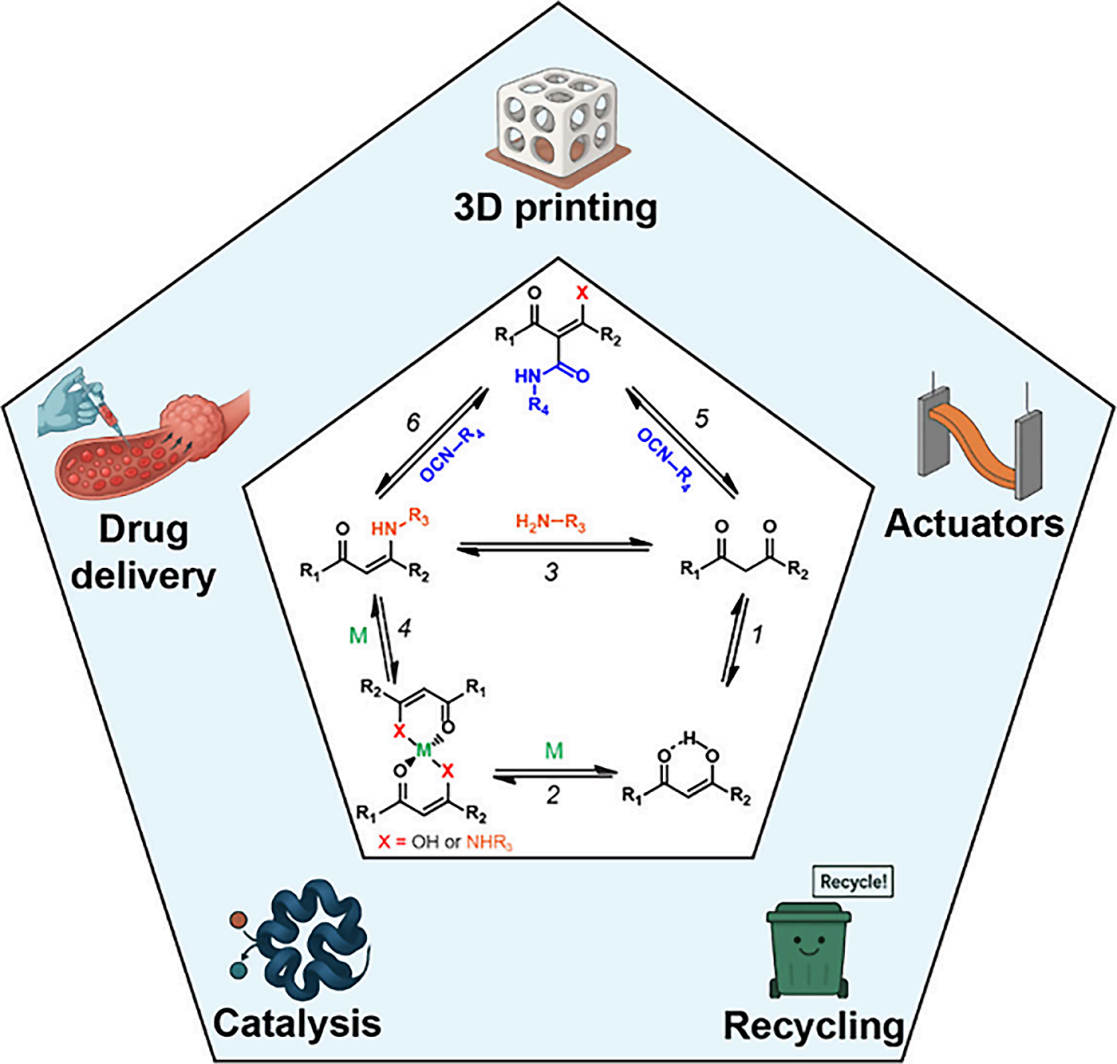
Schematic illustration of the synthesis of dynamic bonds based on the β-dicarbonyl skeleton (inner pentagon): keto–enol tautomerism (1), metal coordination of enol (2), nucleophilic substitution of amine and β-dicarbonyl to afford vinylogous acyl (3), metal coordination of vinylogous acyl (4), nucleophilic addition of β-dicarbonyl and isocyanate to form hydroxylketoenamide (5), nucleophilic addition of vinylogous acyl and isocyanate to produce aminoketoenamide (6), and examples of applications for dynamic polymer materials synthesized from β-dicarbonyl containing starting compounds (outer layer).

The enolic form (or its conjugated base) of the β-dicarbonyl compound can serve as a ligand that coordinates metal ions (2, Figure 2). Nucleophilic attack on one of the carbonyl groups of the β-dicarbonyl skeleton provides synthetic accessibility to vinylogous acyl compounds. Indeed, depending on the nature of the substituents R_1_ and R_2_, vinylogous amides, vinylogous ureas, and vinylogous urethanes can all be prepared through one-step reactions if primary amines are used as nucleophiles (3, Figure 2). These derivatives also possess metal ion coordination capabilities (4, Figure 2). Finally, the β-dicarbonyl skeleton increases the nucleophilicity of the methylene bridge positioned between the two carbonyl groups (α-methylene), consequently unlocking the possibility for addition to isocyanates and the formation of dynamic covalent hydroxylketoenamides (5, Figure 2) or aminoketoenamides (6, Figure 2), depending on the nature of the starting β-dicarbonyl compound.

The large success of β-dicarbonyl chemistry in dynamic polymers is evidenced by a plethora of research studies that showcase its broad applicability in 3D printing,^66^ catalysis,^67^ self-healing and recyclable polymers,^52,68–70^ polymer electrolytes,^71^ light-emitting diodes (LEDs),^72,73^ drug delivery^74,75^ (outer layer, Figure 2), and other applications.^70,74–77^ Here, we summarize the state of research on dynamic polymers based on β-dicarbonyl chemistry and review the fundamental properties and behaviors exhibited by these dynamic polymers. We hope to provide both novice and expert researchers in this field with a comprehensive overview of the potential of β-dicarbonyl chemistry for the preparation of dynamic polymers, as well as a deeper insight into the characteristics exhibited by the different types of β-dicarbonyl-derived dynamic polymers.

## 2 Incorporating β-Dicarbonyl Motifs in Macromolecules

β-Dicarbonyls can be generally classified into three main categories based on the number and type of substituents attached to the dicarbonyl moiety: β-diketones, malonic esters/amides, and β-keto esters/amides (Chart 1). Each of these structural motifs is capable of undergoing tautomerization to form enolates, which accounts for their acidity in aqueous environments and their strong affinity for coordinating metal ions.^78^ Among them, β-keto amides/esters can react with nucleophiles such as amines through either condensation or substitution reactions, while β-diketones and malonic amides/esters can only undergo condensation and substitution reactions, respectively. Although a wide range of β-dicarbonyl small-molecule derivatives are readily accessible,^65,79,80^ only a few of them have so far been employed in the synthesis of β-dicarbonyl-containing polymers, with curcumin (**Cur**), malonic esters, and acetoacetates being the most prominent scaffolds (Chart 1).^52,65,81–83^ This selection is primarily attributed to the presence of additional reactive functional groups in these molecules — such as hydroxyl groups in **Cur** and malonic esters, or (meth)­acrylate and *tert*-butyl moieties in acetoacetates — which facilitate further polymerization or enable chemical modification of other polymers.

**Chart 1:**
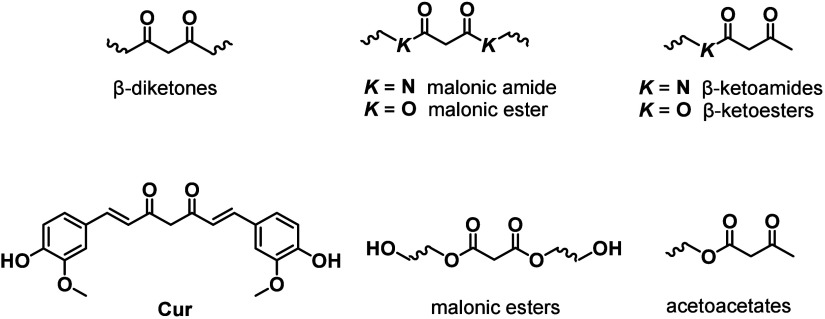
Chemical Structures of the Motifs Discussed in Section 2

The incorporation of β-dicarbonyl motifs into either the side chains or the backbone of macromolecules is generally achieved through one of these four distinct approaches: (1) free or controlled radical (co)­polymerizations of vinyl monomers containing β-dicarbonyl motifs, exemplified by (2-acetoacetoxy)­ethyl methacrylate (**AEMA**) (Figure 3a); (2) the postpolymerization modification of polymers containing hydroxyl groups through transesterification with *tert*-butyl acetoacetate (**tBA**) or of polymers containing hydroxyl- or amino-groups via ring-opening reactions with diketene (Figure 3b); (3) Suzuki polycondensation of monomers comprising β-dicarbonyl motifs in the presence of palladium catalysts (Figure 3c); and (4) polyaddition reactions of β-dicarbonyl-containing dihydroxyl monomers and diisocyanates (Figure 3d).

**Figure 3:**
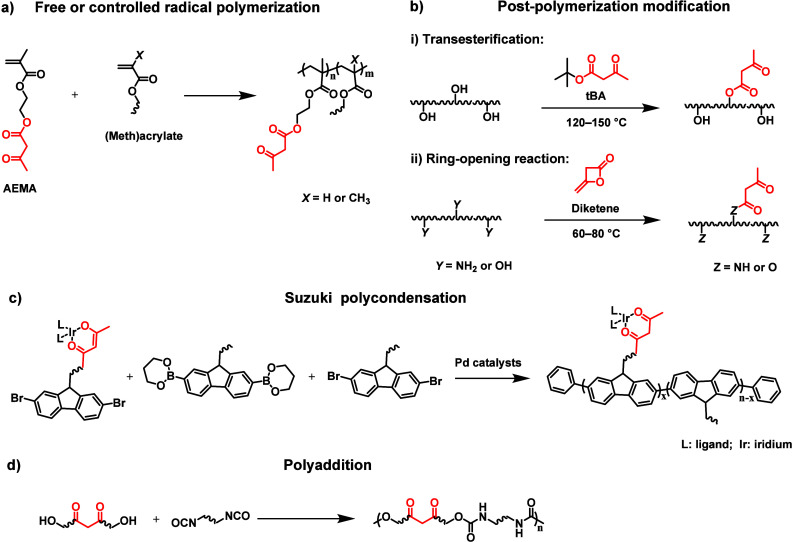
General methods for the incorporation of β-dicarbonyl motifs into the backbone or side chain of macromolecules. (a) Free or controlled radical polymerization of β-dicarbonyl-containing vinyl monomers, such as (2-acetoacetoxy)­ethyl methacrylate (**AEMA**). (b) Postpolymerization modification of (i) hydroxyl-group-containing macromolecules via transesterification with *tert*-butyl acetoacetate (**tBA**) or (ii) hydroxyl- or amino-group-containing macromolecules via ring-opening of diketene. (c) Suzuki polycondensation of β-dicarbonyl-containing dibromide monomers in the presence of palladium catalysts. (d) Polyaddition of β-dicarbonyl-containing dihydroxyl compounds and isocyanates.

Each of these methods requires different reaction conditions and affords polymers whose composition, structure, and properties can be tailored for a wide range of applications. The postpolymerization modification with either **tBA** or diketene, as shown in Figure 3b, generally requires temperatures around 120–150 °C for **tBA** or above 60 °C for ketene but proceeds in the absence of any catalyst.^84–89^ The ease of operation, e.g., typical lack of sensitivity to air and moisture of the starting materials, is one of the main advantages of this approach. Postpolymerization functionalization reactions usually yield polymers that preserve the structures and functions of the starting materials. In stark contrast, the polymerization of β-dicarbonyl-containing monomers via (free) radical polymerization (Figure 3a), Suzuki polycondensation (Figure 3c), and polyaddition (Figure 3d) involves the transformation of small molecules into their corresponding macromolecules. The immediate consequence is that this approach provides access to polymers with distinct structures, features, and properties compared to their small-molecule precursors. Overall, methods for postpolymerization functionalization and direct polymerization are complementary, and their combination allows one to diversify designs, compositions, and functions.

### 2.1 β-Dicarbonyl Motifs as Side Chains

The initial strategy to introduce β-dicarbonyl groups into polymers has been the azodiisobutyronitrile (**AIBN**)-initiated free radical polymerization of ethyl acryloylacetate (**EAA**) or acryloylacetone (**AAe**), which affords poly­(ethyl acryloylacetate) (**PEAA**) or poly­(acryloylacetone) (**PAAe**) (Chart 2), respectively.^90,91^ These early examples were subsequently followed by free radical polymerization approaches to prepare poly­(2-acetoacetoxy ethyl methacrylate) (**PAEMA**)^92^ (Chart 2) and copolymers of methyl (meth)­acrylate and **AEMA**,^93,94^ comprising β-dicarbonyl groups as side chains (Figure 3a). Such polymerizations proceed under mild conditions and are relatively insensitive to moisture and oxygen.^90–93^ The authors demonstrated that the macromolecules thus prepared display a high dispersity (Đ) and that the copolymers made are statistical.^91,93^

**Chart 2:**
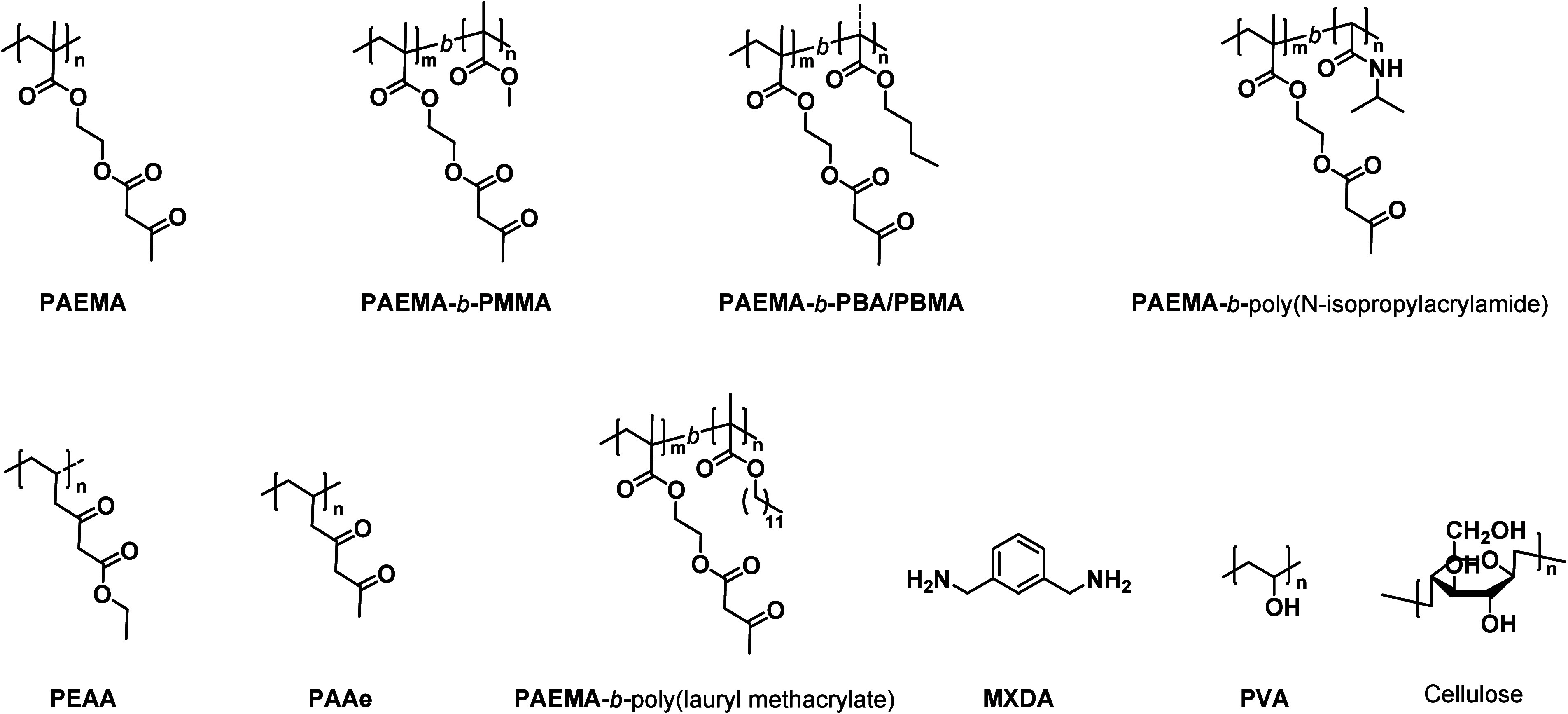
Chemical Structures of the Monomers, Polymers, and Compounds Discussed in Section 2.1

The examples discussed above demonstrate that the use of free radical polymerizations in the synthesis of β-dicarbonyl-containing polymers poses stringent limitations when precise control over molecular weight, dispersity (Đ), and macromolecular architecture is desired. To address these limitations, synthetic approaches were directed toward controlled (radical) polymerization techniques (Figure 3a). The first example of such an endeavor was reported in 2001 by Schlaad and colleagues,^95^ who synthesized poly­(2-hydroxyethyl methacrylate) and poly­(2-hydroxyethyl ethylene) via living anionic polymerization and subsequently introduced β-ketoester groups via transesterification of the hydroxyl groups with **tBA** by thermal treatment at 120 °C. The resulting polymers exhibit a low Đ, in the range of 1.03–1.09. The two-step methodology applied was necessary, as the catalysts (ammonium or *sec*-butyllithium) used in the anionic polymerizations were susceptible to deactivation due to the strong chelation offered by the β-ketoester motif. To circumvent this limitation, Schlaad and co-workers later employed reversible addition–fragmentation chain transfer (RAFT) radical polymerization to directly polymerize **AEMA** without the use of metal ion catalysts.^96^ The authors successfully prepared well-defined **PAEMA** homopolymers and block copolymers of **AEMA** and methyl methacrylate (**MMA**)/*n*-butyl (meth)­acrylate (**nBA**/**BMA**)/*N*-isopropylacrylamide (Chart 2) with Đ = 1.12–1.22, demonstrating a practical route for the direct synthesis of low-Đ, β-ketoester-containing polymers from the readily available **AEMA** monomer.^96^ Further studies involving these well-defined **PAEMA** homopolymers demonstrated their propensity to self-assemble into helical superstructures, characterized by a diameter of approximately 12 nm and a length spanning from 200 to 500 nm.^97^ The formation of these structures was attributed to hydrogen bonding interactions between adjacent β-ketoester groups.^97^

Following the first approaches of Schlaad and co-workers, controlled radical polymerization techniques quickly established themselves as convenient methods to synthesize *well-defined* β-dicarbonyl-containing polymers. Krasia-Christoforou and co-workers conducted a comparative study between block and statistical copolymers of lauryl methacrylate and **AEMA** synthesized using either RAFT or free radical polymerization techniques (**PAEMA**-*b*-poly­(lauryl methacrylate, Chart 2).^98^ The authors found that the copolymer synthesized via RAFT exhibits a low Đ of 1.12, while the counterpart prepared via free radical polymerization has a considerably higher Đ value of 2.9. Moreover, the block copolymer synthesized via RAFT displays superior thermal stability and provides better control over the microphase-separated structure than the statistical copolymer made by free radical polymerization. The block copolymer synthesized by RAFT self-assembles into spherical micelles with hydrodynamic diameters ranging from 30 to 50 nm in dilute hexane solutions.^98^ Sumerlin and co-workers also utilized RAFT to synthesize statistical and block copolymers of **AEMA** and methyl methacrylate^99^ or butyl methacrylate (**BMA**).^83,100,101^ The corresponding vitrimers were created by cross-linking the copolymers with *m*-xylylene diamine (**MXDA**, Chart 2). The authors observed that the block vitrimers exhibit better creep resistance than their statistical counterparts with otherwise similar composition and cross-link density.^83^ The enhanced creep resistance was demonstrated to be linked to the presence of microphase-separated structures in the block copolymers.

In addition to the RAFT technique, nitroxide-mediated free radical polymerization (NMP) also emerged as another viable method to produce β-dicarbonyl-containing polymers. This polymerization technique is tolerant to the presence of a number of functional groups and does not require the use of a metal catalyst.^102^ Jean et al. utilized NMP to synthesize homopolymers, and random and block *co*-/*ter*-polymers bearing β-diketone side groups with predictable molecular weights, compositions, and low Đ values.^72^ The resulting polymers were applied as matrices for doping luminescent molecules, thereby enabling the fabrication of solution-processed, white-light LEDs.

Metal ion-driven polymerization techniques have also been applied for the preparation of polymers featuring β-dicarbonyl motifs. Braddock et al. employed ring-opening metathesis polymerization (ROMP) to polymerize norbornene monomers containing a bis­(ketonato)­palladium­(II) complex in the presence of a ruthenium catalyst, resulting in a polymer with a loading of palladium­(II) of 23 wt %.^103^ Similarly, Hudson and co-workers demonstrated the applicability of atom transfer radical polymerization (ATRP) to polymerize acrylic monomers containing an iridium­(III)−β-ketoester complex, yielding polymers with low Đ values (from 1.08 to 1.14) and molecular weights of up to 40 kDa.^104^ The resulting iridium−β-ketoester coordination polymers exhibit promising optoelectronic properties. Later, Singha and co-workers reported the synthesis of **PAEMA** via the ATRP technique as well.^105^ The coordination of the β-dicarbonyl motifs installed in **PAEMA** and cobalt­(II) ions afforded a metallopolymer with superparamagnetic behavior.^105^ These papers introduced another viable technique for the polymerization of vinyl monomers containing β-dicarbonyl moieties,^105^ even though Schlaad and co-workers earlier mentioned that the presence of β-dicarbonyl groups might hinder ATRP by sequestering the metal ion catalyst.^96^

The postpolymerization modification of commercial and natural polymers is another well-established approach for the introduction of β-dicarbonyl functionalities (Figure 3b). The process can be achieved by targeting hydroxyl or amine groups through two distinct approaches. The first strategy involves the **tBA**-facilitated transesterification of hydroxyl groups present along the macromolecular chain, which is typically carried out at high temperatures (120–150 °C) (Figure 3b, (i). The transesterification involving a hydroxyl group and **tBA** has been extensively employed in the postmodification of synthetic polymers such as poly­(vinyl alcohol) (**PVA**) (Chart 2),^106,107^ poly­(ethylene glycol) (**PEG**),^71,108–110^ poly­(propylene glycol) (**PPG**),^86^ fluorinated polyether diol,^87^ as well as natural polymers or compounds, including cellulose (Chart 2),^111–114^ starch,^88,89^ and castor oil.^115,116^ The acetoacetylation agent, i.e., **tBA**, serves as both solvent and reagent in this process, pushing the reaction to completion on account of the Le Chatelier principle. The removal of *tert*-butanol, the reaction byproduct (using a Dean–Stark apparatus, for example), further increases the conversion of the hydroxyl moieties into the desired acetoacetate groups. However, the high temperatures (120–150 °C) required for this chemistry restrict the approach to functionalizing polymers that are sufficiently stable. Another limitation of this method is that it is not applicable for the conversion of amino groups due to their high reactivity toward **tBA**. Thus, the acetoacetylation of amino groups is typically carried out by resorting to the second approach shown in Figure 3b, ii, which involves the ring-opening reaction of diketenes with amines at lower temperatures (60–80 °C).^84,85^ It should be noted that diketene is also effective in introducing β-ketoester motifs starting from hydroxyl-containing polymers via direct reaction with the hydroxyl groups at 60 °C, overcoming some of the limitations of the transesterification method relying on **tBA**.^117^ However, the high versatility and effectiveness of diketene as an acetoacetylating agent are counterbalanced by its carcinogenic nature and propensity to hydrolyze into acetoacetic acid.^118^

While free and controlled radical polymerization protocols are frequently employed for the polymerization of vinyl monomers, compounds bearing other types of functional groups, such as dihydroxyl and dibromide compounds, are suited for polycondensation and polyaddition reactions. For example, palladium-catalyzed Suzuki polycondensations have been shown to provide efficient polymerizations of β-dicarbonyl-containing dibromide monomers (Figure 3c).^119–124^ Several groups have reported that dibromide monomers bearing an iridium−β-dicarbonyl complex — obtained via the coordination of iridium ions by the β-dicarbonyl motif — can afford iridium-doped metallopolymers in the presence of palladium catalysts. These metallopolymers have been utilized as phosphorescent substrates for LEDs.^119–124^

### 2.2 β-Dicarbonyl Motifs in the Backbone

The methods for the integration of β-dicarbonyl functionalities summarized above primarily involve their attachment to the side chains of polymers. In contrast, polyaddition reactions of β-dicarbonyl-containing dihydroxyl- and di-isocyanate-groups allow one to engineer *polymer backbones* comprising β-dicarbonyl motifs (Figure 3d). The naturally occurring compound **Cur** (Chart 1), which is extracted from the turmeric rhizome and has been used for thousands of years as a spice and dye, is a widely employed molecule featuring the β-dicarbonyl motif.^65^ **Cur** has garnered significant attention in medicinal chemistry because of its antimicrobial and antiviral properties. Previous studies have extensively investigated **Cur** and its derivatives in small-molecule form for molecular imaging and therapeutics.^80,82,125,126^ However, recent studies have explored the incorporation of **Cur** into polymer architectures, which is accessible by reacting the two phenolic hydroxyl groups with dianhydrides, divinyl ethers, dichlorophosphate (polycondensations),^127^ or with isocyanates (polyadditions).^128–130^ For example, Bao and co-workers reported the incorporation of β-dicarbonyl groups into the backbone of polyurethanes through a polyaddition involving **Cur**, isophorone diisocyanate (**IPDI**), and polytetrahydrofuran diol (Chart 3).^131^ The resulting polyurethanes are able to chelate europium ions (Eu^3+^), forming metallopolymers thanks to the presence of the β-diketone moiety in **Cur**.

**Chart 3:**
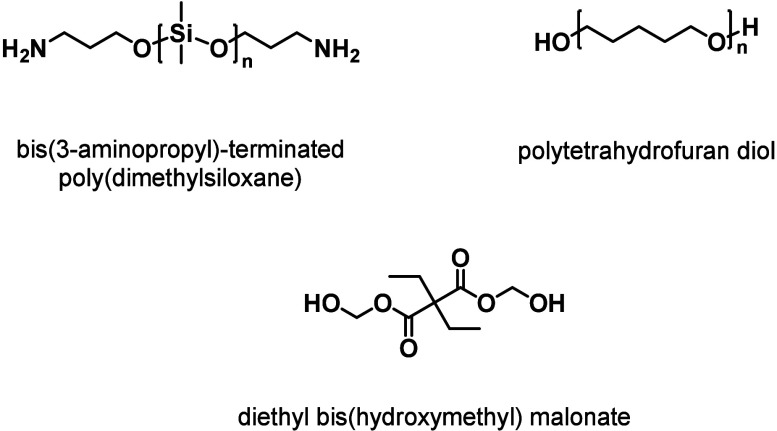
Chemical Structures of Some of the Structural Components of the Polymers Discussed in Section 2.2

Shi and co-workers employed a similar approach to synthesize poly­(urethane–urea)­s featuring β-diester motifs along the main chain via the polyaddition of bis­(3-aminopropyl)-terminated poly­(dimethylsiloxane), **IPDI**, and diethyl bis­(hydroxymethyl) malonate (Chart 3).^132^ These poly­(urethane–urea)­s were also able to coordinate Eu^3+^ and could be assembled into metallosupramolecular polymers that are held together by Eu^3+^–coordination.^132^ The authors also reported that the dynamic coordination of Eu^3+^ ions by the β-diester functionalities provides excellent mechanical properties and reversible stimuli-responsive fluorescence.^132^

The incorporation of β-dicarbonyl functionalities into polymers through direct polymerization of β-dicarbonyl-containing monomers or postmodification strategies affords polymer platforms with high versatility for further customization. Indeed, the engineered β-dicarbonyl groups can serve as reactive sites for subsequent reactions with alkenes, aldehydes, or amines, thereby accessing tailored polymer properties.^133^ These newly introduced functional groups can also serve as cross-linkable sites, thus offering the opportunity to manipulate polymer topology. Additionally, the chelating capability of β-dicarbonyl functionalities enables (transition) metal coordination, which can consequently impart new optical, electrical, magnetic, and antibacterial properties/functions.^134,135^

Despite the various approaches discussed in this section, incorporating β-dicarbonyls along the polymer backbone appears to be a less flourishing approach compared to side-chain functionalizations. This difference might be attributed to the large number of (commercially available) macromolecular architectures featuring hydroxyl- and amino-groups as functionalities on the side chain and the popularity of controlled/living polymerization techniques, which allows the direct introduction of the desired β-dicarbonyl moiety. Nevertheless, the development of polymers containing β-dicarbonyls directly installed on the backbone is a fundamentally interesting challenge that can lead to attractive characteristics. Indeed, the manipulation and functionalization of the polymer backbone offer the prospect of influencing materials’ properties significantly.

## 3 Classification of Dynamic Polymers Synthesized from β-Dicarbonyl Motifs

As discussed in Section 1, β-dicarbonyl moieties are versatile chemical skeletons that give place to different types of **DB**s thanks to their reactivity toward metal ions, electrophiles, and nucleophiles. Examples of **DB**s derived from β-dicarbonyl moieties include supramolecular systems assembled by hydrogen bonds or metal ion−β-dicarbonyl coordination, and **DCB**s such as enamides, vinylogous urethanes (**VU**s), and diketoenamines (Figure 4).

**Figure 4:**
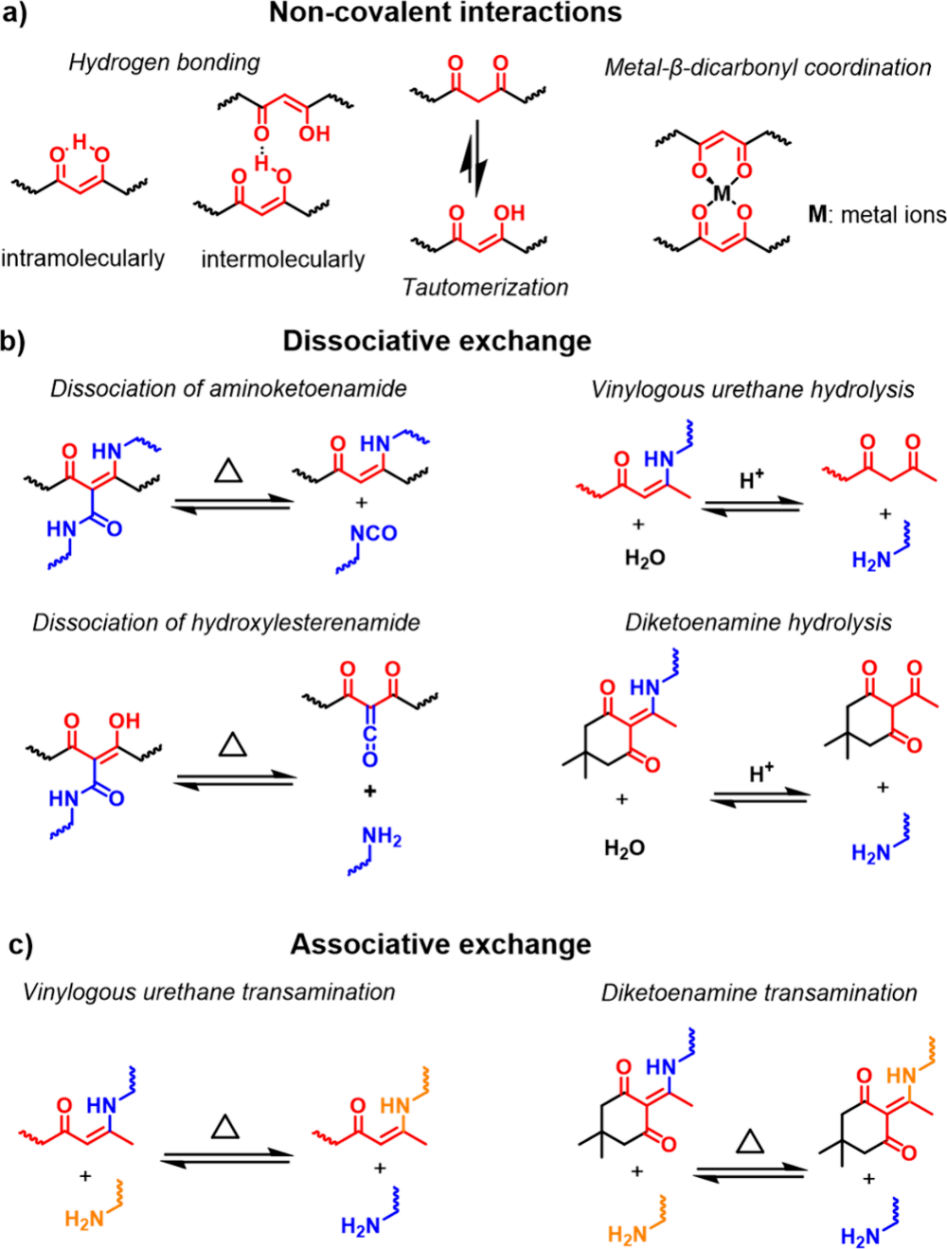
Dynamic chemistries derived from β-dicarbonyl motifs based on (a) noncovalent interactions, (b) dissociative exchange, and (c) associative exchange.

Studies on hydrogen bonding interactions in polymeric materials comprising unmodified β-dicarbonyl motifs are rather limited, possibly due to the weak strength of hydrogen bonding interactions they form, and the efforts dedicated to this topic focused primarily on understanding the self-assembly behavior of polymers in solutions.^97,98^ The exploitation of β-dicarbonyl motifs as hydrogen bonding donors and acceptors is complicated by the competition between inter- and intramolecular processes, as the tautomeric enol form of β-dicarbonyl skeletons is stabilized by intramolecular hydrogen bonding (Figure 4a). This prototropic equilibrium is influenced by parameters such as temperature, solvent and polymer polarity, acidity/basicity of the medium, and has triggered the formation of phase-separated structures in block copolymers featuring low-polarity chain skeletons.^83,98,136^

β-Dicarbonyls are prone to form coordination bonds with metal ions due to the back-donation of electron density from the metal to the π* antibonding orbitals of the carbonyl ligands (Figure 4a, right).^134^ Studies that have explored the coordination between metal ions and β-dicarbonyl motifs in polymers are abundant, and the metallosupramolecular interaction has been shown to endow the resulting polymers with a broad array of functionalities, depending on the polymer substrates, metal ions, and the specific metal−β-dicarbonyl coordination chemistry involved.^137,138^ The presence of metal ions enriches the properties of the resulting polymers, which can be further tuned through the selection of the metal ion, counteranion, and temperature. These parameters influence the binding constant of the complex, as well as the bond energy, which can range from the level of van der Waals interactions (0.4–4 kJ/mol) to that of covalent bonds (≥150 kJ/mol).

The β-dicarbonyl-derived **DCB**s mainly comprise enamide derivatives, **VU**s, and diketoenamines (Figure 4b–c). Hydroxylketoenamides and aminoketoenamides are enamide derivatives that undergo chemical exchange through a dissociative pathway in which they dissociate into ketenes (in the case of hydroxylketoenamides) or isocyanates (in the case of aminoketoenamides) and their respective constituents upon heating (Figure 4b).^76,139–142^ Such dissociative mechanisms have been supported by temperature-variable Fourier transform infrared spectroscopy (FT-IR) experiments.^76,139–142^

**DCN**s featuring **VU** or diketoenamine linkages have been extensively investigated both in terms of structure–property relationship and applications.^143,144^ Differently from hydroxylketoenamides and aminoketoenamides, **VU**s and diketoenamines follow either dissociative (i.e., hydrolysis and condensation) or associative (i.e., transamination) mechanisms depending on the chemical environment (Figures 4b–c). For instance, Du Prez and co-workers showed that upon heating above 100 °C, **VU**s undergo transamination reactions in the presence of free amino groups, passing through iminium intermediates (Figure 4c).^52,53^ However, when the same chemistry is conducted in polymer networks initially lacking free amines, the transamination pathway is significantly suppressed, thereby inhibiting vitrimer behavior.^145^ Subsequent investigations from the Abetz lab revealed that the presence of free amines in the initial polymer networks is not a stringent requirement for reversibility, as it can be compensated by a combined action of a Bro̷nsted acid and water.^146^ The Bro̷nsted acid protonates the **VU** motifs and converts them into iminium intermediates that can be rapidly hydrolyzed, releasing free amines (i.e., dissociation). The amines thus liberated engage in transamination reactions with nonhydrolyzed **VU** linkages upon heating, thereby triggering the exchange dynamics (Figure 4b).^146^ Recent studies from Weder, Berrocal, Shi, and co-workers have indicated that **VU** networks containing highly polar frameworks hydrolyze into their constituent materials (i.e., β-ketoesters and amines) when exposed to an excess of water, even in the absence of acids (Figure 4b).^108^ Conversely, the hydrolysis is significantly impeded in **VU** networks with less polar characteristics.^108^ Altogether, these findings stress the importance of chemical composition and chemical environment in directing the dynamic behavior of **VU** linkages. Similar conclusions are applicable to polymers based on the diketoenamine motif.^54,143,147,148^ Thus, these studies^52,53,108,145,146^ may serve as guiding principles for the newly emerging triketoenamines^149^ and other analogous hydrolyzable chemical motifs such as dioxaborolanes,^150,151^ acetals,^152–154^ and imines.^155–157^

Having discussed general aspects of β-dicarbonyl-containing dynamic polymers, it should be mentioned that metal−β-dicarbonyl coordination polymers and **DCN**s leveraging **DCB**s derived from β-dicarbonyl motifs possess different properties. The metal−β-dicarbonyl coordination polymers integrate the flexibility and viscoelasticity of polymers with the features of the metal ions incorporated. This combination can impart properties that are sometimes beyond the reach of conventional polymers. For instance, most traditional organic polymers (composed of carbon, hydrogen, nitrogen, and sulfur) lack the magnetic properties that are often observed in materials comprising iron, nickel, or cobalt. Kohri and co-workers showed that β-dicarbonyl-containing polymers can become magnetic upon incorporation of terbium nanoparticles.^92^ Polymers based on the coordination of metal ions by β-dicarbonyl skeletons typically feature reversibility and faster responsiveness to external stimuli than β-dicarbonyl-derived **DCN**s, which often translates into a lower thermodynamic stability/robustness of the materials.

Overall, metal−β-dicarbonyl coordination polymers often leverage the unique properties of metallic species and dynamic coordination bonds,^158,159^ while **DCN**s focus on the robustness and reversibility of **DCB**s.^18,40,160,161^ These differences result in different properties, and hence different potential applications for these two classes of materials. The specific characteristics, design principles, and applications of β-dicarbonyl-derived dynamic polymers will be elaborated meticulously in the following sections.

## 4 Polymers Comprising Metal−β-Dicarbonyl Interactions

Metallo-supramolecular polymers are polymer complexes formed through coordination interactions between metal ions and ligand-containing polymers.^162^ These systems generally integrate the flexibility and viscoelasticity of polymers with the optical, electrical, and or/magnetic properties brought by the metallic species. One of their appealing characteristics is the possibility to modulate the strength of the metal–ligand coordination, which influences the final materials’ properties, by chemical design.^162–167^ The judicious selection of the ligands and metal ions allows the design of healable polymers, provided that weak and dynamic complexes with bond strengths of ca. 100–200 kJ/mol are selected.^168,169^ Furthermore, the incorporation of functional metal ions endows these polymers with diverse functionalities, including specific dielectric properties, luminescence, magnetism, and catalytic activity, among other possibilities.^168^ For example, Shi and co-workers reported on the intense fluorescence of β-diester-containing poly­(urethane–urea)­s upon coordination with Eu^3+^ ions, whose properties contrast starkly with the absence of emission in blends of model poly­(urethane–urea)­s that lack the β-diester ligand and an equivalent amount of Eu^3+^ salt.^132^ This highlights the critical role of the β-diester motif in enhancing the fluorescence of Eu^3**+**^ by acting as an antenna that absorbs UV light and transfers the absorbed energy to the metal ion.

The deprotonation of the enol form of β-dicarbonyl motifs produces a species with high chelating power that is capable of binding to many different metal ions. As numerous reviews have already discussed the well-established small-molecule behavior of such complexes in great depth,^65,170–174^ we limit our discussion to the development of polymers comprising metal−β-dicarbonyl complexes. Our overview is structured in three sections that focus on the salient features of such complexes, namely (1) the intrinsic optical, electronic, magnetic, and catalytic characteristics, which are transferred to the polymer, (2) the impact of the (dynamic) coordination bonds on the polymers’ structure and thus thermal and mechanical properties, and (3) the synergistic effects between (1) and (2) and the emerging characteristics/functions that they bring to the polymer. We note that, in principle, all polymers comprising metal−β-dicarbonyl complexes exhibit properties that are impacted by different features, even if our discussion is limited to the specific aspects highlighted in the original works.

### 4.1 Intrinsic Optical, Electronic, Magnetic, and Catalytic Characteristics of the Metal–Ligand Complexes Transferred to the Polymer

The incorporation of metal ions in metal−β-dicarbonyl supramolecular polymers can transfer intrinsic features of these complexes to the resulting polymers. For example, iridium−β-dicarbonyl metallopolymers have been reported to be phosphorescent due to the presence of iridium ions.^72,119–121,123,124,175^ The color emitted stems from a combination of the iridium−β-dicarbonyl complexes and other chromophores in the metallopolymers. Various emission colors have been reported in the literature, including orange,^175^ white,^72,119,121,123^ red,^120,124^ and green.^72^ Cao and co-workers prepared metallo-supramolecular polymers with benzothiadiazole units (Chart 4) incorporated into the backbone of polyfluorene (Chart 4) and iridium−β-dicarbonyl complexes on the side chain.^123^ By adjusting the composition of these two sets of chromophores, it was possible to create metallo-supramolecular polymers that display white light emission as a result of the combination of blue emission of the polyfluorene, green emission of the benzothiadiazole units, and red emission of the iridium−β-dicarbonyl complexes.

**Chart 4:**
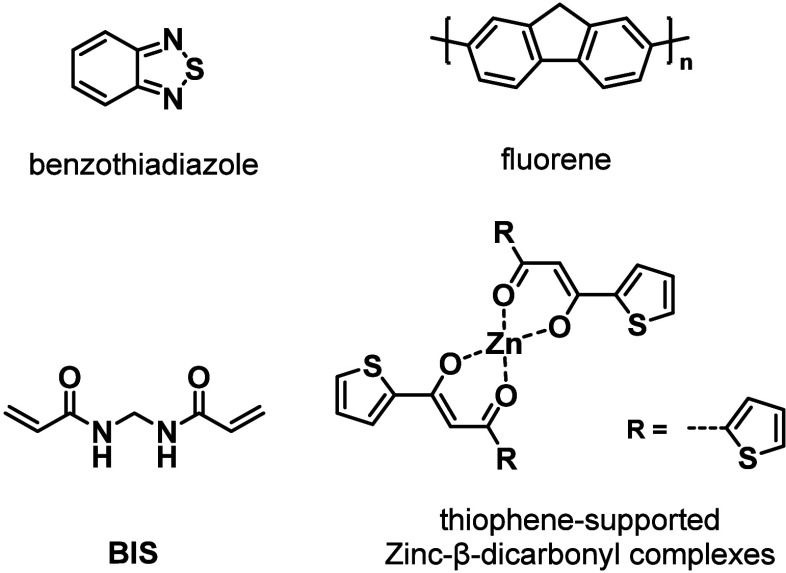
Chemical Structures of the Structural Components of the Polymers Discussed in Sections 4.1–4.2

Introducing lanthanide ions such as Eu^3+^,^92,176–178^ terbium (Tb^3+^),^92,123^ samarium (Sm^3+^),^73^ or neodymium (Nd^3+^)^158^ into β-dicarbonyl polymers can bestow the resulting polymer complexes not only with fluorescence but also magnetic properties. Kohri and co-workers reported the preparation of polymer complexes by doping lanthanides into poly­(β-ketoester) particles that were synthesized via dispersion polymerization of **AEMA** and *N*,*N*′-methylenebis­(acrylamide) (**BIS**) (Figure 5a, i and Chart 4).^92^ The polymer complexes thus made exhibit magnetism and fluoresce upon irradiation with UV light. The materials were reported to be useful in magnetic inks and anticounterfeiting materials (Figure 5a, (ii). Besides lanthanides, the addition of iron oxide^105^ or cobalt oxide^105,179^ into β-ketoester polymers has also been shown to afford magnetic materials. Moreover, González et al. demonstrated that introducing zinc ions into β-dicarbonyl polymers can confer catalytic activity.^67^ The authors prepared zinc-coordinated metallopolymers via electropolymerization of zinc−β-dicarbonyl complexes bearing thiophenyl groups (Chart 4).^67^ The resulting polymers proved to be catalytically active in the ring-opening polymerization of lactic acid to produce polylactic acids.^67^

**Figure 5:**
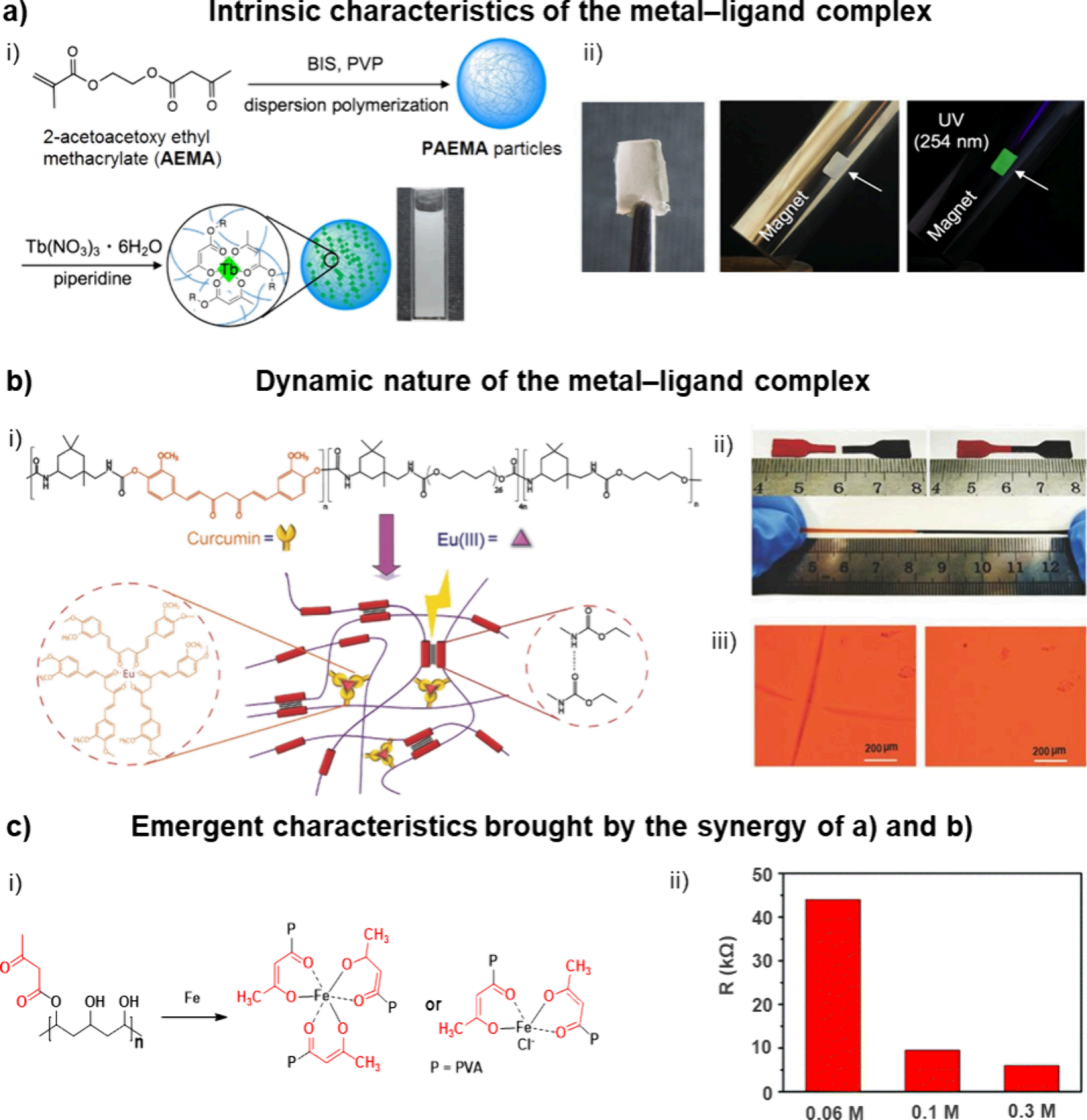
(a) Example of a β-ketoester-containing metallopolymer whose properties reflect an intrinsic feature of the metal–ligand complex. (i) Preparation of **PAEMA** and **PAEMA/Tb** metallopolymers; (ii) photos of a free-standing **PMMA** film containing the **PAEMA/Tb** complexes (left), and its attachment on a magnet under exposure to ambient light (middle) or UV light (λ = 254 nm) (right). Adapted with permission from ref 92. Copyright 2020 the American Chemical Society. (b) Example of a β-dicarbonyl-containing metallopolymer whose properties rely on the dynamic nature of the coordination bonds. (i) Molecular structure of **Cur**-based polymer and its coordination of Eu­(III); (ii) welding of the **Cur**–metallosupramolecular polymer at 25 °C for 24 h (top), and subsequent stretching test of the welded film (bottom); (iii) optical images of the **Cur**–metallosupramolecular polymer before (left) and after (right) being healed at 25 °C for 24 h. Adapted with permission from ref 131. Copyright 2018 Wiley. (c) Example of a β-ketoester-containing metallopolymer whose properties rely on a combination of intrinsic and dynamic features of the metal–ligand complex. (i) Coordination of Fe^3+^ by β-ketoester grafted **PVA**; (ii) electrical resistance of the **PVAA–Fe** hydrogels as a function of Fe^3**+**^ concentration. Adapted with permission from ref 187. Copyright 2019 the Royal Society of Chemistry.

### 4.2 Dynamic Nature of the Coordination Bonds and Their Effect on Polymers’ Structure and Mechanical Properties

Many complexes can form spontaneously and are thus thermodynamically stable, yet they are kinetically labile.^180^ The term “thermodynamically stable” refers to the fact that the Gibbs free energy of the complexation process is negative, i.e., the complex formation is energetically favored, while “kinetically labile” means that the ligand exchange among complexes is nevertheless feasible.^168^ Generally, complexes between metal ions and β-dicarbonyl-based ligands are kinetically labile. As such, polymers comprising these coordination species are dynamic (through rapid ligand exchange), a characteristic that has been exploited in self-healing,^68,131^ gas sensing,^181^ and metal adsorption.^182–185^ For instance, Beata et al. synthesized cross-linked poly­(methacrylic acid)­s (**PMAA**s) bearing β-ketoester groups for the solid-phase extraction of ruthenium, exploiting the strong chelating ability of these ligands.^182^ The ruthenium thus captured could be eluted and then recovered by passing a dilute acidic thiourea solution through an ion exchange process.^182^ Bao, Jia, and co-workers incorporated **Cur** (Chart 1) into polyurethanes and subsequently formed supramolecular networks via coordination of Eu^3+^ (Figure 5b, (i).^131^ These networks combine the mechanical properties of a robust elastomer (stress and strain at break of ca. 1.8 MPa and ca. 900%, respectively) with highly efficient self-healing (98% efficiency at 25 °C after 48 h), thanks to the robustness and dynamicity of the Eu^3+^–β-dicarbonyl complexes.^131^ The authors showed that the metallosupramolecular materials can be used to prepare capacitive sensors that enable the fabrication of stretchable and self-healing touch pads.^131^

### 4.3 Synergistic Effects of the Metal–Ligand Complexes and Emergent Characteristics/Functions of the Polymers

The examples discussed in Sections 4.1 and 4.2 showcase either the optical, electronic, magnetic, and catalytic properties, or the dynamic characteristics derived from the presence of the metal ions or the rapid ligand exchange of the metal−β-dicarbonyl complexes, respectively. However, the simultaneous presence of both chemical features offers the potential of creating materials that can leverage the synergy of all these characteristics. For instance, polymethacrylates comprising Eu^3+^–β-dicarbonyl complexes have been reported to be fluorescent (due to the presence of Eu^3+^ ions) and dynamic.^186^ The materials have been used to detect Cu^2+^ ions in aqueous solution and HCl gas through Eu^3+^–fluorescence quenching. The minimal detection threshold for Cu^2+^ ions was as low as 2.0 × 10^–8^ M at pH 7.^186^ Additionally, the red emission from the polymer complexes — which was attributed to the characteristic emission of Eu^3+^ centered at 612 nm — could be switched “off” and “on” upon exposure to HCl and ammonia vapors, respectively.^186^

The Xu lab has been active in exploring the possibility of introducing conductive and dynamic characteristics in polymer materials incorporating metal−β-dicarbonyl complexes. Initial efforts involved the synthesis of hydrogels from β-ketoester-modified **PVA** and Fe^3+^ ions, in which the Fe^3+^–β-ketoester complexes serve as cross-links (Figure 5c, (i).^187^ These hydrogels display remarkable self-healing capabilities as well as pH-, redox-, light-, and temperature-responsive behavior.^187^ Moreover, the presence of Fe^3+^ ions imparts the hydrogels with ionic conductivity. Increasing the concentration of Fe^3+^ ions from 0.06 to 0.3 M caused a reduction of the hydrogel’s resistance by an order of magnitude (Figure 5c, (ii). Xu and co-workers further embedded Fe^3+^ ions in a double-network hydrogel based on polyacrylamide (**PAM**) and **PVA**.^106^ The double-network hydrogel exhibited high extensibility (>700%), high conductivity, good healing efficiency (80% at room temperature within 24 h), and excellent fatigue resistance.^106^ The coordination between Fe^3+^ and **PVA** provides ionic conductivity and self-healing ability to the double network hydrogel, while the **PAM** network offers high stretchability and compressibility. The same group also combined **PVA** with catechol-modified chitosan (Chart 5). The authors first created a homogeneous mixture of **PVA** and catechol-modified chitosan, and subsequently added Fe^3+^ ions that coordinate both β-ketoesters and catechols, affording metallosupramolecolar hydrogels.^188^ The hydrogels exhibit good adhesion (adhesive strength to porcine skin of 102 kPa), rapid self-healing capabilities (96% healing efficiency at room temperature within 5 min), pH responsiveness, and toughness of up to 1386 kJ m^–3^. The authors also demonstrated that the hydrogels can serve as wearable sensors for detecting human movement, and also as bioelectrodes for electrocardiography.^188^

**Chart 5:**
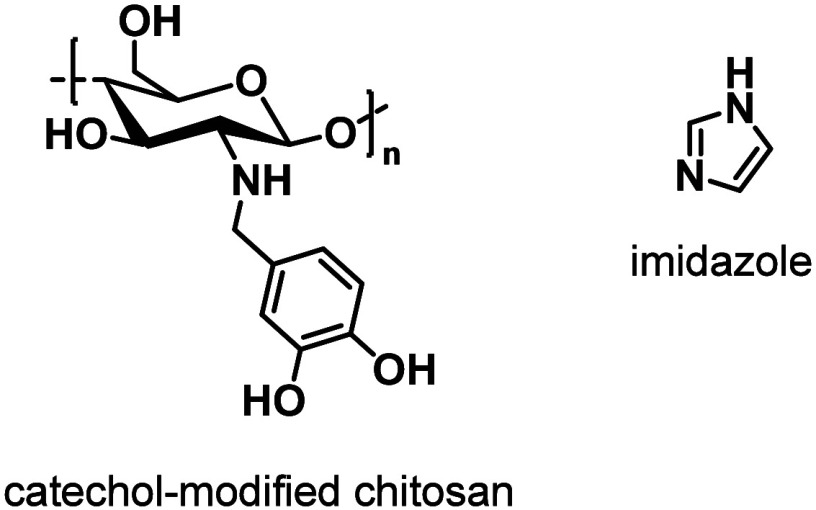
Chemical Structures of the Structural Components of the Polymers Discussed in Section 4.3

The complexation of metal ions through the β-diketone moiety of **Cur** (i.e., curcumin) affords multifunctional coordination polymers. Jiang and co-workers reported the synthesis of novel polyurethane adhesives incorporating **Cur** into the backbone and imidazole (Chart 5) as chain ends.^189^ Upon complexation of Cu^2+^ ions by the **Cur** moieties, the resulting polymer complexes exhibit strong adhesion to various material surfaces, particularly metal surfaces, thanks to the surface–polymer coordination interactions mediated by the imidazole moieties. When exposed to NIR light for 30 s, the supramolecular adhesive would debond due to the disassembly of the metal–ligand coordination bonds. Such dissociation was induced by the photothermal effect mediated by the Cu^2+^ ions. The responsiveness to NIR light also favored the self-healing of the polymer complexes upon irradiation for 5 min. Density functional theory (DFT) calculations revealed that the coordination bonds comprised stronger Cu^2+^–**Cur** coordination and weaker Cu^2+^–imidazole coordination.^189^ The synergy of this hierarchical dynamic structure justified the strong adhesive strength and rapid adhesion switching speed, which are intrinsically contradictory features, of the adhesives.^189^

## 5 Vinylogous Urethane Dynamic Covalent Networks

Vinylogous urethanes (**VU**s) are **DCB**s that can be accessed via either the condensation of β-ketoesters and primary amines,^52^ or a click reaction between alkyne esters and primary or secondary amines.^190^ The establishment of **VU**s in the portfolio of **DCB**s dates back to 2014, when Pomposo and co-workers reported that β-ketoesters and primary amines react to give enamine bonds that can be used as dynamic cross-links to modulate the topologies of single-chain polymer nanoparticles by controlling the pH.^191^ A year later, Du Prez and co-workers brought enamine linkages to the realm of bulk polymer materials by preparing **DCN**s comprising these bonds.^52^ Admittedly, the term “vinylogous urethane” was coined for the enamine moiety in this work as the skeleton of the **VU** motif resembles that of a carbamate — the structural motif present in polyurethanes — albeit with a vinylic bond inserted in between the C = O and the nitrogen atom.^52^ The authors found that **VU**s undergo dynamic exchange according to an associative pathway upon heating to between 100 and 140 °C (Figure 4c), which brought **VU**-containing **DCN**s to enter the field of vitrimers, as the network topology can be rearranged without sacrificing its integrity (i.e., the cross-link density remains largely unchanged).^151,192–194^ While the exchange is possible in the absence of any catalyst, the exchange kinetics of **VU**s can be modulated by the addition of acid or base catalysts, enabling the deliberate design of **VU** vitrimers with predictable and adjustable viscoelastic properties.^53^ These seminal findings have sparked significant interest within the scientific community and fostered rapid advancements in the exploration of structure–property relationships and applications of **VU** vitrimers.

In this section, we first engage in a detailed examination of the influence exerted by the specific chemical structure of the **VU**, the nature of the catalyst, and the network topology on the exchange kinetics and dynamic properties of **VU**-based **DCN**s. These chemical insights can serve as guidelines for the design of reprocessable **VU** polymers, which are discussed in Section 5.2. Aside from being reprocessable, **VU** polymer networks are also chemically recyclable. The closed-loop recycling of **VU** polymers and factors influencing it — including cross-link density, molecular weight, and polarity of polymer skeleton, among others — form the topic of Section 5.3. Finally, Section 5.4 deals with the design principles of recently developed click strategies for the synthesis of **VU** polymer networks, which are attractive alternatives to the polycondensation methods relying on the reaction of β-ketoesters and primary amines. The click-synthesis of **VU** polymers can be advantageous in controlling the ratio between *cis* and *trans* isomers and in avoiding water as a synthetic byproduct, which is characteristic of polycondensations.

### 5.1 Influence of the Chemical Structure on the Dynamics of VU Polymer Networks

**VU** linkages undergo transamination reactions in the presence of free amines when brought to sufficiently high temperatures (Figure 4c, left). This chemical process is the pillar of the dynamic behavior of **VU DCN**s. As demonstrated by work carried out on small-molecule **VU** model compounds, the kinetics of the transamination reaction are influenced by steric effects, electronic effects, and the presence (and nature) of external or internal catalysts (i.e., neighboring group participation effects).^53,195–197^ These examples suggest that transamination processes are facilitated by the presence of bulky groups, extending the conjugation of the amino groups in the **VU** compounds,^198^ and the addition of Lewis and Bro̷nsted acids.^53^ The number of parameters controlling the **VU** exchange significantly increases when considering polymer systems, with network topology,^83^ chain length,^100^ chain rigidity,^199^ and spatial proximity between **VU** motifs and free amines^200^ also playing important roles. All these parameters affect the ability of the networks to become sufficiently dynamic and flow.^53,83,100,196,198,200^ Consequently, the meticulous design of chemical structures and chemical environments at the (macro)­molecular level enables the precise modulation of the dynamic characteristics of **VU** linkages and tailoring of the properties of the materials in which they are embedded.

As previously mentioned, the presence of free amines is crucial for the transamination reaction between **VU** linkages. This concept was mentioned in the seminal work from the Du Prez lab,^52^ albeit experimental verification was not initially provided in this study. Further collaborative work between the Du Prez and Leibler laboratories solidified this concept through the exploration of polydimethylsiloxane (**PDMS**)-based **VU** vitrimers (Figure 6a).^145^ The behavior of **VU** vitrimers featuring free amino groups was compared to that of equally built **VU**-based polymers in which the amine functionalities were “masked” as methyl acetoacetate derivatives by postsynthetic functionalization.^145^ The authors showed that thermal reshaping and reprocessing are only possible in the **VU** vitrimers comprising free amines, which proved to be a key structural requisite for a dynamic exchange. Instead, masking the amines with methyl acetoacetate significantly quenches the transamination (Figure 6a, (i)), as evidenced by the suppression of the stress relaxation behavior in the networks (Figure 6a, (ii)).

**Figure 6:**
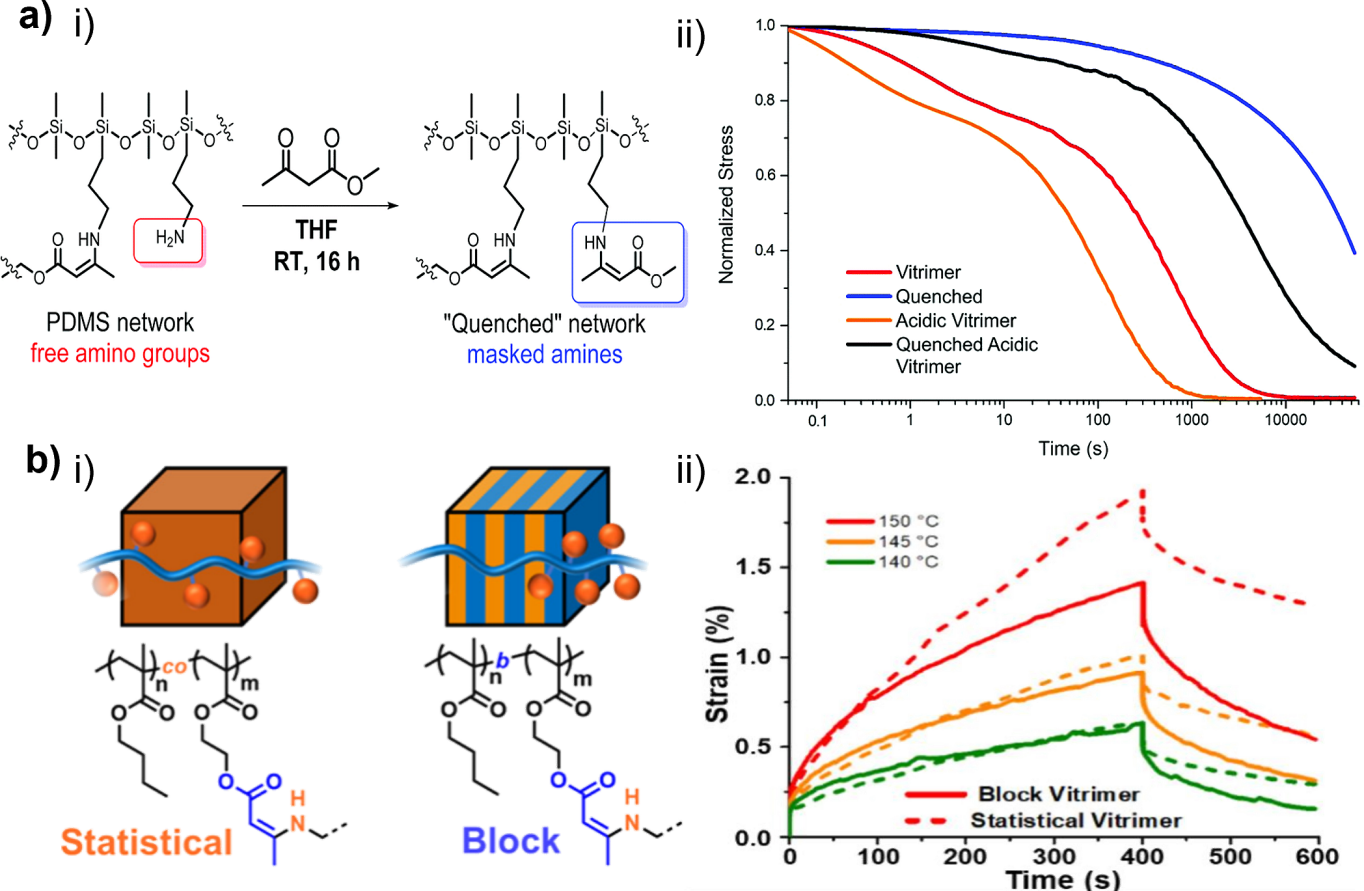
(a) Influence of free amino groups on the dynamics of **VU** vitrimers: (i) synthesis of the quenched **PDMS-**based **VU** vitrimer; (ii) normalized stress relaxation profiles (2% strain, 100 °C) of the **PDMS-**based **VU** vitrimer under different experimental conditions. Adapted with permission from ref 145. Copyright 2017 the Royal Society of Chemistry. (b) Comparison of statistical and block **VU** vitrimers prepared from (i) butyl methacrylate (**BMA**) and (2-acetoacetoxy)­ethyl methacrylate (**AEMA**); (ii) Overlaid creep recovery curves of the statistical and block vitrimers at 140, 145, and 150 °C at a constant stress of 5 kPa. Adapted with permission from ref 83. Copyright 2020 the American Chemical Society.

Following a similar line of thought, Zhou and co-workers systematically investigated the dynamic behavior of polyether-based **VU** vitrimers.^86^ The authors found that increasing the number of free amino groups accelerates the transamination of **VU** moieties, ultimately increasing the stress–relaxation rates of the **VU** vitrimers. Moreover, the cross-link density and the content of free amines in the vitrimers control the topology freezing temperature (*T*_*v*_), which marks the onset of the dynamic behavior of the network; the higher the cross-link density or content of free amines, the lower the *T*_*v*_.^86^

Although initial reports highlighted the key role of free amines in enabling exchange dynamics in **VU** networks, recent studies by Abetz and co-workers demonstrate that transamination reactions can also occur if the original material does not contain any free amino groups.^146^ Indeed, free amines can be produced *in situ* in the presence of a Bro̷nsted acid catalyst and water and facilitate the dynamic reaction.^146^ The Bro̷nsted acid protonates the **VU** motif, transforming it into an electrophilic iminium intermediate. Nucleophilic attack by water triggers the dissociation of the iminium intermediate, with the consequent release of a free amine that can engage in the transamination mechanism, as discussed above (Figure 4c, left).^146^ The dissociation also produces free β-ketoester motifs that can recombine with the generated free amines and form the **VU** motif again. Thus, this work introduced an additional approach that allows triggering dynamic exchanges among **VU** linkages, even in the absence of free amino groups in the original **VU**-based polymers.

Du Prez and co-workers demonstrated that the viscoelastic characteristics of **VU** vitrimers — in particular stress relaxation — not only depend on free amino functionalities and catalysts present in the polymer networks but are also influenced by the rigidity and polarity of the backbone, molecular weight of the starting monomers, and the cross-link density.^199^ For instance, variations in the molecular weight yield vitrimers with notably distinct activation energies for stress relaxation (from 68 to 149 kJ mol^–1^), although the authors stated that their work did not “establish clear relationships between these parameters and the stress relaxation properties”.^199^

The intricate interplay between multiple factors makes it difficult to develop a general framework to predict the relaxation kinetics of **VU** vitrimers. In an attempt to fill this fundamental gap and establish structure–reactivity relationships at the (macro)­molecular level, Sumerlin and co-workers studied the influence of the molecular weight of polymer constituents by copolymerizing **AEMA** and **BMA** using RAFT, which afforded copolymers **P­(BMA**-*co*-**AEMA)**s (Chart 6) with different yet controlled molecular weights.^100^ These building blocks were subsequently cross-linked with tris­(2-aminoethyl)­amine (**TREN**, Chart 6) to afford **VU** vitrimers with the same cross-link density and free amine content but different average molecular weights between cross-links.^100^ Stress relaxation measurements reveal that increasing the molecular weights leads to decreased stress relaxation rates, i.e., higher values of activation energy (*E*_a_) for viscous flow. These variations became more evident beyond the entanglement molecular weight threshold. The study also emphasized the necessity of taking not only the average molecular weight into account but also the molecular weight distribution when assessing their impact on the ability to flow of **VU** vitrimers.^100^

**Chart 6:**
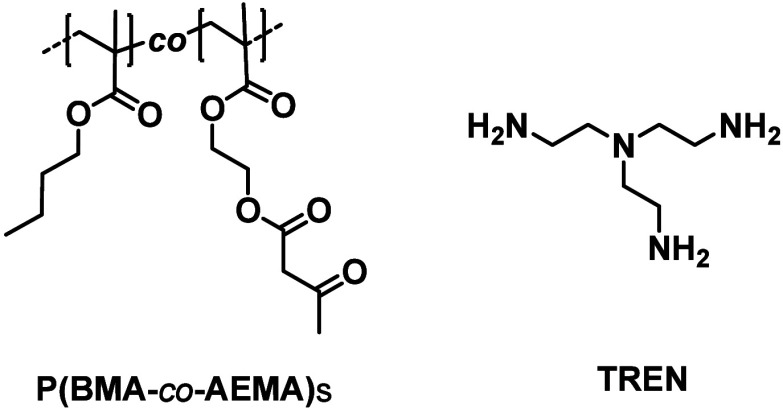
Chemical Structures of the Structural Components of the Polymers Discussed in Section 5.1

The relationship between structure and exchange kinetics at the molecular level was investigated by Ruipérez and co-workers in a computational study on the transamination of amines and vinylogous acyls, in particular vinylogous ureas and vinylogous urethanes.^198^ The computations were carried out using density functional theory (DFT) calculations at the 6–311++G­(2df,2p) and Def2TZVPP level of theory. The authors reported that vinylogous ureas exhibit faster exchange kinetics and lower activation energies for the transamination with free amines than vinylogous urethanes. This difference was attributed to hydrogen bonding interactions that stabilize both transition states and intermediates in vinylogous ureas.^198^ Steric hindrance also plays an important role, as the presence of bulky amino groups in vinylogous acyls was found to promote transamination. The influence of the chemical nature of the amine moiety on transamination was also investigated by comparing the reactivity of vinylogous urethane toward benzylamine or aniline. Aniline was found to be less prone to undergo a nucleophilic attack on the vinylic moiety of vinylogous urethane than benzylamine on account of its lower nucleophilicity. On the other hand, the reaction with aniline affords a more conjugated structure, which favors the protonation of the vinylic α-carbon in the exchange process.^198^ Thus, knowledge on the basicity/nucleophilicity and the extent of conjugation of free amines is useful to predict and control the exchange kinetics involving vinylogous urethanes and free amines.^198^ The theoretical work from the Ruipérez lab provides important guidelines for the design of **VU** vitrimers with targeted viscoelastic properties through the judicious choice of network constituents.

The complex interplay of multiple parameters, jointly influencing the chain dynamics of **VU** vitrimers, has been further corroborated by a recent collaboration between the Du Prez and Rowan groups.^201^ The authors observed that, in solution, small molecules containing vinylogous urea residues exhibit faster bond exchange rates than the vinylogous urethane analogs, as predicted by the theoretical results reported by Ruipérez and co-workers.^198^ However, when the same reactive moieties were incorporated as cross-links in macromolecular systems, comparable stress relaxation and creep behavior were observed. Interestingly, the simultaneous presence of vinylogous urethane (10 mol %) and vinylogous urea (90 mol %) linkages in the same polymer led to an order of magnitude acceleration in the stress-relaxation rate at 150 °C. The authors attributed such acceleration to intermolecular hydrogen bonding interactions (brought by the presence of vinylogous urea moieties) that catalyze the exchange reaction in the networks. The study also showed that this effect has an optimum, as elevated concentrations of vinylogous ureas (above 50 mol %) led to phase separation and slowed down the interchain dynamics of the vitrimers (i.e., suppressing creep) at temperatures below 90 °C.^201^ Nevertheless, the study suggests that combining vinylogous urethane and vinylogous urea linkages is an additional tool for designing **DCN**s with controllable chain dynamics. Finally, the macromolecular architecture of **VU** vitrimers can also play a crucial role in the **VU** exchange dynamics.^83,202^ Sumerlin and co-workers used the RAFT polymerization of **BMA** and **AEMA** to synthesize **P­(BMA**-*co*-**AEMA)** and **P­(BMA**-*b*-**AEMA**), a statistical and a block copolymer, respectively, with comparable molecular weights and compositions. **P­(BMA**-*co*-**AEMA)** and **P­(BMA**-*b*-**AEMA)** were then transformed into **VU** vitrimers by a cross-linking reaction with an equivalent amount of **MXDA** (Chart 2) (Figure 6b, (i)).^83^ The **VU** vitrimer derived from the block copolymer (block vitrimer) self-assembles under formation of a lamellar morphology, a behavior that was not observed for the **VU** vitrimer based on the statistical copolymer (statistical vitrimer). This structural difference endowed the block vitrimer with a superior resistance to macroscopic deformation in comparison to the statistical vitrimer (Figure 6b, (ii)). The study was an early comprehensive investigation of the influence of **VU** networks’ topology on their viscoelastic flow and sparked further exploration of other **DCN**s resorting to imine^203^ and boronic ester **DCB**s.^204,205^

### 5.2 Influence of the Chemical Structure of VU Polymer Networks on Their Reprocessing Capabilities

One of the most exciting aspects related to **DCN**s lies in the possibility of overcoming the lack of reprocessability and (chemical) recyclability of conventional thermosets, thus extending the life cycle of the materials. **VU**-based **DCNs** are no exception in this regard, and **VU** linkages have been exploited to cross-link poly­(meth)­acrylates,^99–101,206,207^ polystyrenes,^208^ polyethylenes,^209^ fluorinated polymers,^210,211^ polyureas, polyurethanes,^66,212^ and epoxy resins with the goal of producing recyclable/reprocessable networks.^213–216^ The Sumerlin group synthesized poly­(methyl methacrylate) (**PMMA**)-based **VU** vitrimers by performing a RAFT copolymerization of methyl methacrylate (**MMA**) and **AEMA**, and subsequently cross-linking the copolymer by means of **TREN** (Figure 7a, (i)).^99^ The resulting **VU** vitrimer displays exceptional reprocessability and retains its chemical structure and mechanical properties over six (re)­processing cycles (Figure 7a, (ii)).^99^ Expanding on this strategy, Urban and co-workers introduced *n*-butyl acrylate (**nBA**) as an additional comonomer in the RAFT copolymerization of **MMA** and **AEMA** and cross-linked the resulting terpolymer using **TREN** (Figure 7b, (i)).^206^ The copolymer network is reprocessable via compression molding at 120 °C over four cycles (Figure 7b, (ii)), and displays notable self-healing capabilities under ambient conditions, both before and after compression molding, as evidenced by optical microscopy (Figure 7b, (iii)) and tensile testing (Figure 7b, (iv)). The authors attributed the room-temperature self-healing behavior to a synergistic interplay between the reversible *E* ⇌ *Z* isomerization of the vinylic moieties of the **VU** functionality and van der Waals interactions.^206^

**Figure 7:**
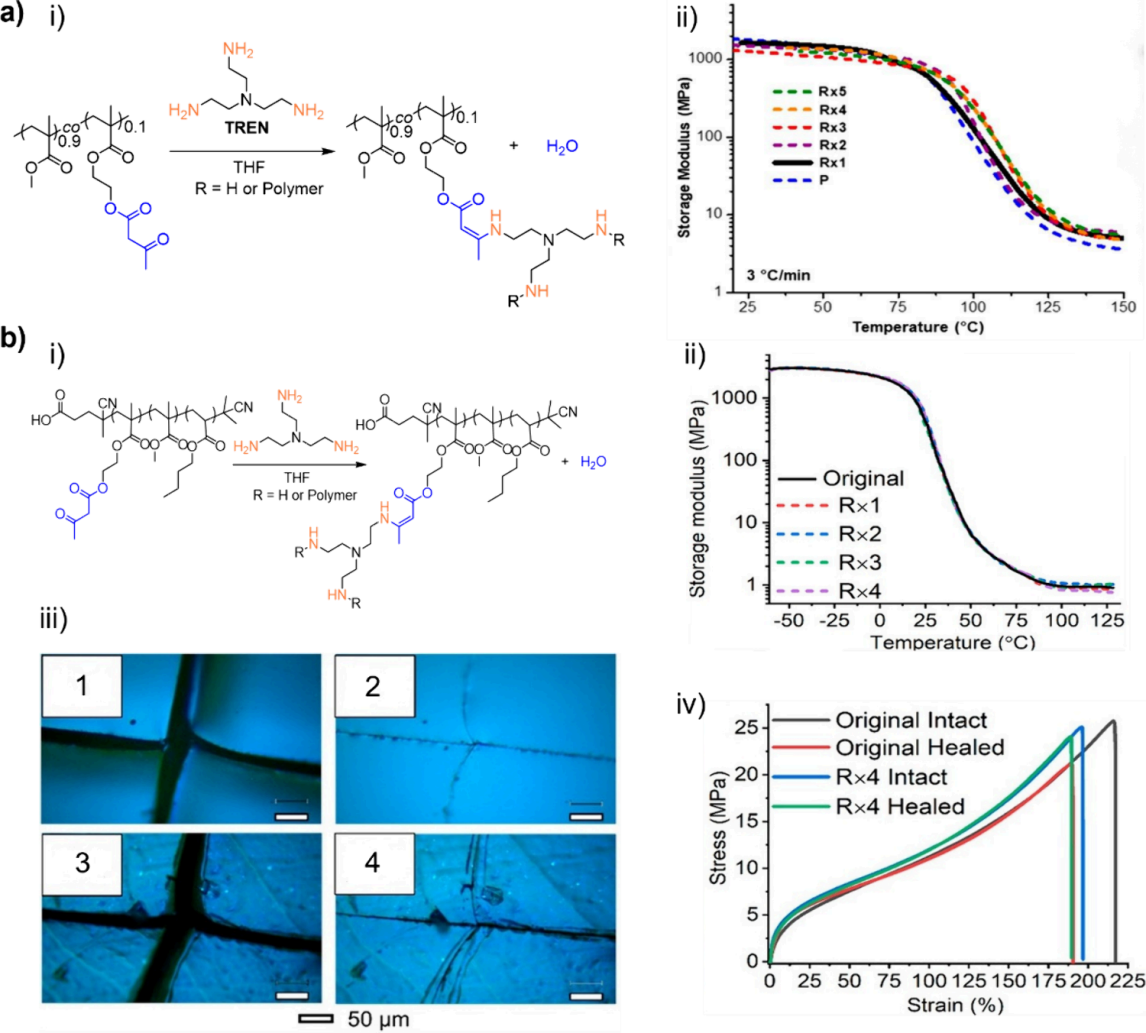
(a) (i) Synthesis of **PMMA**-based **VU** vitrimer via cross-linking of the copolymer of **MMA** and **AEMA** with **TREN**; (ii) Comparison of the dynamic mechanical analysis (DMA) traces of **PMMA**-based **VU** vitrimer as prepared and after multiple (5x) reprocessing cycles. Adapted with permission from ref 99. Copyright 2019 the American Chemical Society. (b) (i) Synthesis of acrylate-based **VU** vitrimer via cross-linking reaction of the terpolymer prepared from **MMA**, **AEMA**, and **nBA** with **TREN**; (ii) Comparison of the dynamic mechanical analysis (DMA) traces of the acrylate-based **VU** vitrimer as prepared and after multiple (4x) reprocessing cycles; (iii) Optical images of the original acrylate-based **VU** vitrimer before (1) and after (2) being healed at room temperature for 24 h, and of the same vitrimer after the fourth reprocessing before (3) and after (4) being healed at room temperature for 24 h (iii); (iv) Stress–strain curves of the intact and healed specimens of the pristine vitrimer and after the fourth reprocessing cycle (R × 4). Adapted with permission from ref 206. Copyright 2022 the American Chemical Society.

In addition to the challenges associated with recycling and reprocessing, accessing polymers from renewable feedstocks has been a topic of particular interest in promoting the circularity of plastics and releasing the pressure on oil-derived chemicals.^217^ The **VU** functionality presents a promising avenue for exploring renewable resources in the preparation of **VU** vitrimers. Castor oil and its derivatives,^115,116,218^ vegetable oils,^219^ cardanol,^220^ vanillin,^221^ (all shown in Chart 7) and polysaccharides^222,223^ have all been successfully employed to synthesize **VU** vitrimers. Leveraging the dynamic chemistry of **VU**s, Wang and co-workers devised a straightforward approach for synthesizing polyamide elastomers derived from castor oil that combine recyclabiliy and weldability.^218^ Hydrogen bonding interactions between the amide moieties of the polymer backbone facilitates strain-induced crystallization, which is responsible for a significant increase in the tensile strength of the vitrimers (from 35 to 156 MPa) after a mechanical training process consisting of repetitive cyclic stretching.^218^ Lin, Sheng, and co-workers described another interesting approach to exploit the dynamic character of **VU** linkages by compression molding two biobased **VU** vitrimers possessing distinct mechanical properties into a new one.^220^ The two **VU** vitrimers were prepared by reacting cardanol acetoacetates with **MXDA** (Chart 2) or 4,4-diaminocyclohexylmethane (**PACM**, Chart 7), and the two polymers were compression molded at 130 °C and 10 MPa for 40 min. This treatment (carried out in three rounds) facilitated the exchange between the two polymers and afforded a material with comparable mechanical and thermal properties to a homogeneous **VU** vitrimer prepared by directly combining cardanol acetoacetates, **MXDA**, and **PACM** in the same ratio. Extrapolating from this example, melt-mixing techniques (which also incorporate compression molding) could allow one to tune vitrimers’ properties in a gradient manner and could be applicable to **DCN**s employing other **DCB**s.

**Chart 7:**
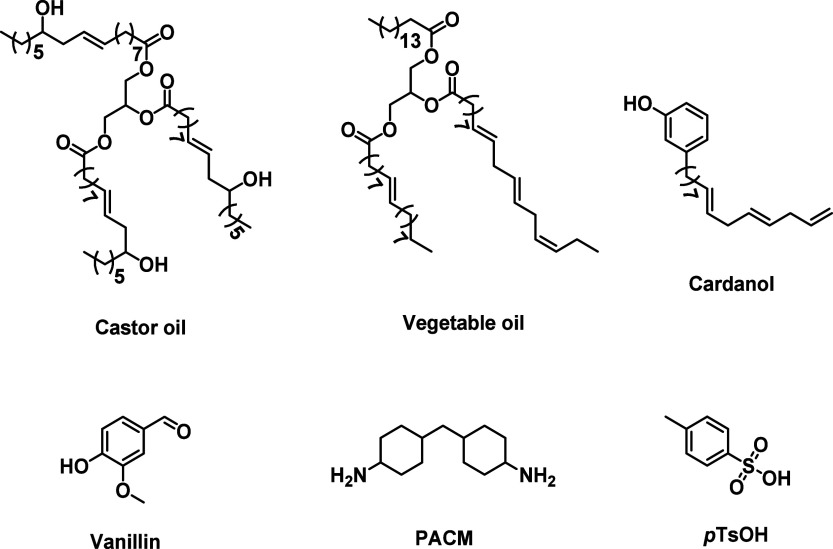
Chemical Structures of the Structural Components of the Polymers Discussed in Section 5.2

A major limitation that has hindered the technology transfer of **VU** vitrimers from academic research to industrial manufacturing thus far is related to the long relaxation times displayed by most **VU** vitrimers, which do not meet the requirements of high-speed processing demanded by melt extrusion and injection molding.^39^ The Du Prez group has provided important insights on how this limitation may perhaps be overcome. In a first investigation, the team demonstrated that the combination of *p*-toluenesulfonic acid (*p* **TsOH**, Chart 7) as a catalyst and placing pendant, free amino groups in close proximity to the **VU** linkages in the polymer architecture affords stress relaxation times lower than 1 s at 150 °C.^200^ The same group later noticed that replacing **VU** linkages with vinylogous ureas, together with the addition of a catalyst, also accelerates stress relaxation.^224^ Indeed, they reported a stress relaxation time as low as 2.4 s at 170 °C for poly­(vinylogous urea) networks upon incorporation of 0.5 mol % of *p* **TsOH** as catalyst.^224^ In hindsight, such fast stress relaxation behavior can probably be ascribed to the strong hydrogen bonding interactions between vinylogous urea groups, which stabilizes the intermediate states along the transamination pathway, as suggested by the computational investigations of the Ruipérez group (cf. Section 5.1).^198^

Another bottleneck preventing the industrialization of **VU** vitrimers is the irreversible plastic deformation (or creep) at service temperature.^195^ This phenomenon can lead to premature rupture or displacement of polymers during service, and should thus be prevented.^145^ Both creep and (re)­processing of **VU** vitrimers are rooted in the dynamic exchange between **VU** bonds and free amines, therefore maintaining high dynamics at reprocessing temperatures while pursuing creep–resistance is a formidable challenge.^195,225^ As the presence of free amines is essential in determining the exchange kinetics of the transamination of **VU** linkages (cf. Section 5.1),^52,145^ an immediate approach to suppressing creep should involve masking the free amines or reducing their content in the **VU** networks. However, this action will negatively impact the reprocessability of the material, highlighting the necessity to devise more creative alternatives. In this context, Du Prez and co-workers proposed in a recent study the addition of small quantities of dimethyl glutarate dibasic ester (**DBE-5**) in the architecture of the **VU** vitrimers to consume free amino groups via formation of dicarboxamide moieties (Figure 8b).^226^ A control **VU** vitrimer without **DBE-5** was also prepared for reasons of comparison (Figure 8a). It was shown that the addition of the **DBE-5** limited the availability of amines for exchange and consequently suppressed creep at low temperatures. On the other hand, reprocessing and rapid material flow of the **VU** vitrimers were obtained at elevated temperatures (Figure 8d, left), as the dicarboxamides are thermally reversible and release free amines upon heating (Figure 8d, right). Moreover, the temperature dependence of the stress–relaxation rate of the dibasic ester containing **VU** vitrimers (Figure 8d) differed dramatically from that of the control **VU** vitrimer (Figure 8c).^226^ The former displayed a distinct acceleration of the relaxation rate in a confined temperature window (Figure 8d), while the latter appeared to be more gradual (Figure 8c).^226^ Overall, this strategy proved to be effective in hindering creep without hampering the reprocessability of the vitrimers, providing a valuable guideline for other vitrimer systems.

**Figure 8:**
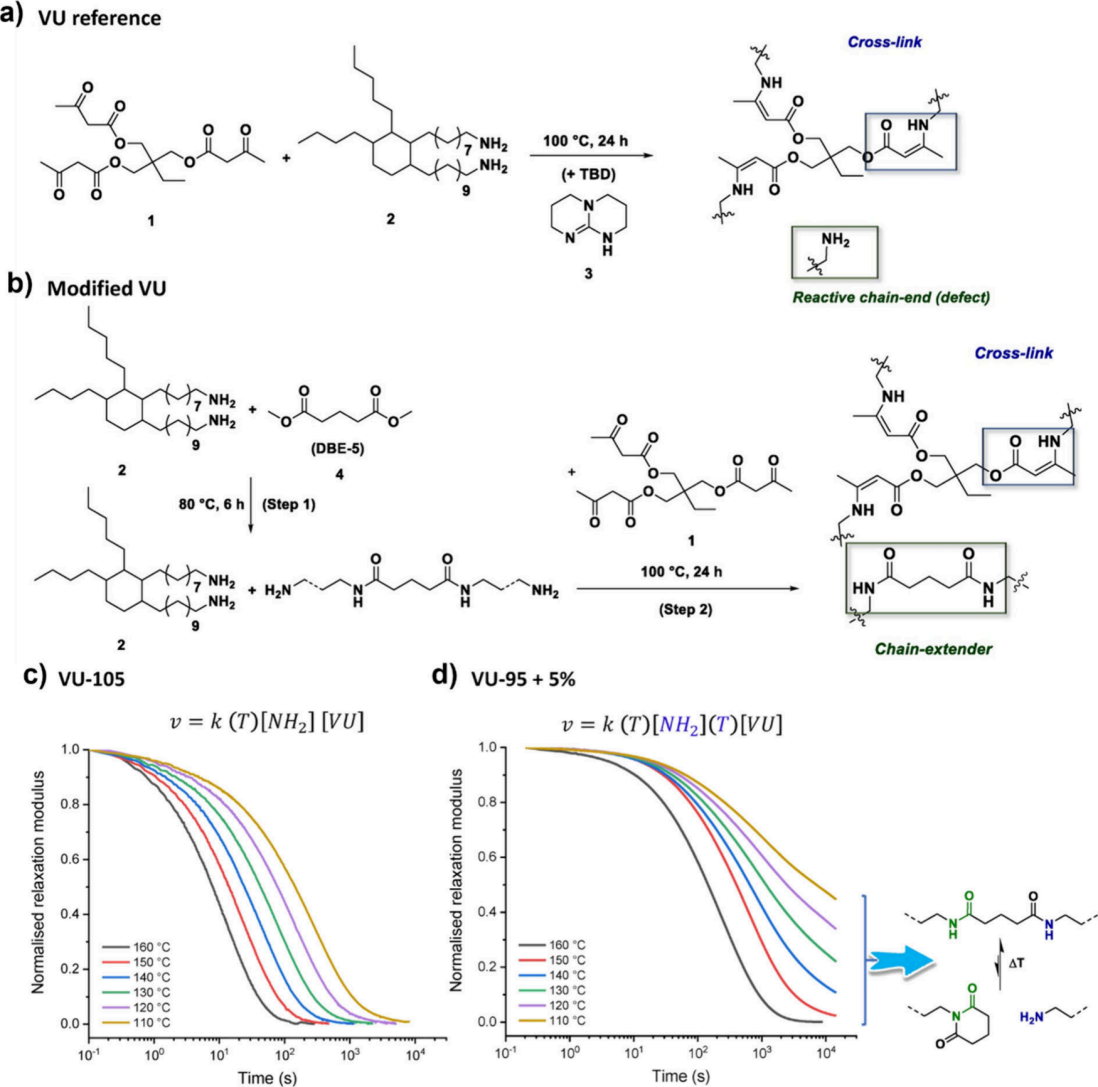
Synthesis of (a) reference **VU** vitrimers promoted by triazabicyclodecene (**TBD**), and (b) modified **VU** vitrimers in the presence of 1–20 mol % of **DBE-5** and excess amines, followed by cross-linking with acetoacetate derivatives. Normalized stress relaxation profiles (0.5% strain) of (c) reference and (d) modified **VU** vitrimers measured between 110 and 160 °C. Adapted with permission from ref 226. Copyright 2022 Wiley.

The Du Prez group subsequently expanded the successful dibasic ester approach described above to related strategies. For example, the temporary sequestration of the free amines in **VU** vitrimers was performed by resorting to acrylates that engage in aza-Michael reactions, yielding dynamic β-amino esters.^227^ This design largely suppresses creep, while the materials’ reprocessability is hardly affected, as free amines can be released via thermally induced dissociation of the β-amino esters (Figure 9a). In another approach, triethylenetetramine was introduced as a comonomer in the curing of **VU** formulation, which results in a **VU** vitrimer that does not feature reactive primary amines.^228^ This strategy attenuates creep on account of the lower reactivity of secondary amines toward **VU** linkages at lower temperatures. Nevertheless, the exchange between **VU** linkages and proximal secondary amines at elevated temperatures liberated primary amines, which reinstated the vitrimers’ ability to flow (Figure 9b).

**Figure 9:**
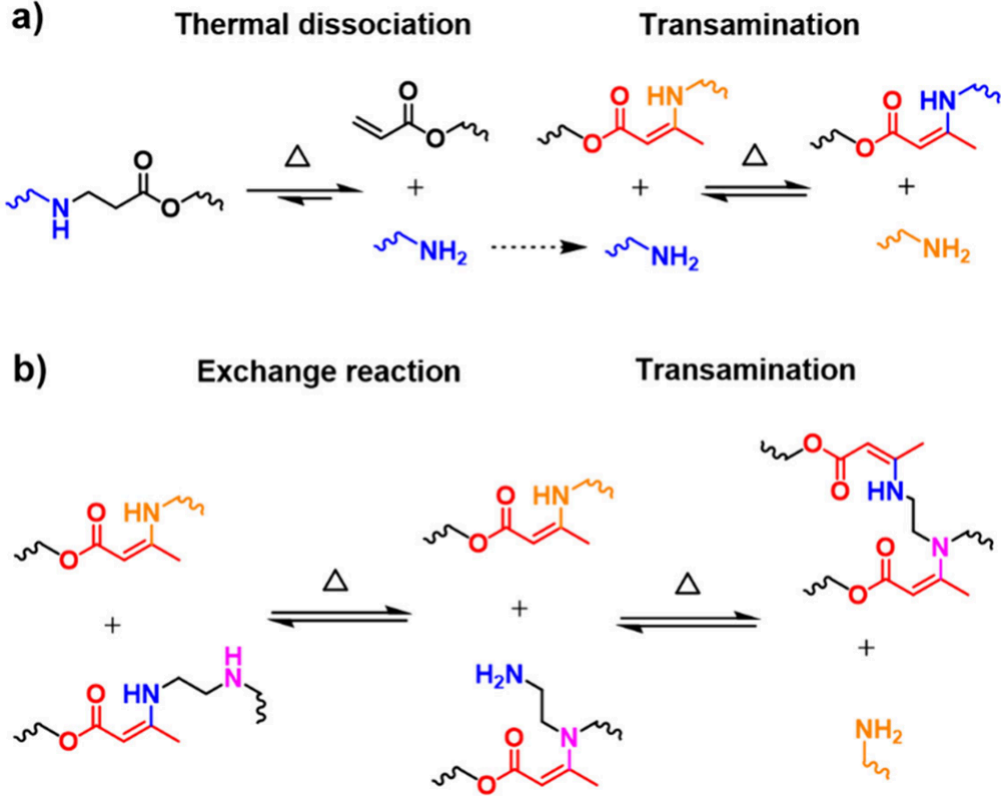
(a) Thermal dissociation of β-amino ester into acrylate and amine (left), followed by the transamination between the dissociated amine and a **VU** linkage. Adapted with permission from ref 227. Copyright 2022 the Royal Society of Chemistry. (b) Exchange between a **VU** moiety and a secondary amine to afford a pendant primary amine and a secondary amine-substituted **VU**, followed by the subsequent transamination of the released primary amine with **VU**. Adapted with permission from ref 228. Copyright 2022 the American Chemical Society.

The underlying principle of all the approaches described above consists of regulating the availability of free primary amines by masking them at the service temperature and releasing them at elevated temperatures to allow for (re)­processing. The chemical “trick” employed is based on the use of a second moiety that stores the primary amines in the form of a “dormant” chemical functionality that can be activated at higher temperature. The successful execution of this concept allows controlling the viscous flow of **VU** vitrimers on demand.

Masking the free amines influences the viscoelastic behavior of **VU** networks by controlling the kinetics of exchange at the molecular level. However, as previously stated, other *macro*molecular parameters, such as the chemical characteristics of the polymer (e.g., polarity, rigidity, conformation), network topology, and material morphology, also play important roles. For example, microphase-separation or constraints imposed by supramolecular interactions (e.g., hydrogen bonds) could also hinder creep without affecting reprocessability.^83,201^ As discussed in Section 5.1, the incorporation of large amounts of vinylogous ureas (above 50 mol %) into a **VU** network can suppress creep below 90 °C due to the strong hydrogen bonding interactions brought by the vinylogous urea linkages, which consequently leads to phase separation.^195^ A recent study from the Sumerlin group revealed that introducing guanine–cytosine hydrogen bonding motifs (10 mol %) into **VU** networks imparted significant creep resistance, even at 150 °C.^229^

Other strategies that involve the incorporation of latent catalysts,^230–232^ clever choice of protecting groups,^195^ increasing the valency of cross-linkers,^233^ and neighboring group participation^197,234–236^ have been explored in other classes of **DCN**s, and have been summarized in a recent review by Du Prez, Winne, and co-workers.^195^ For example, an intriguing report from the Helms group describes that engineering polytopic cross-linking functionality at the chain ends of flexible polyetheramines significantly reduces the creep of the resulting polymer networks to less than 1%, in stark contrast to 200% of the control sample featuring monotopic cross-links.^233^ These methods could be borrowed for further development of creep-resistant, highly reprocessable **VU** vitrimers.

### 5.3 Closed-Loop Recycling of VU Polymer Networks

Dynamic polymer materials offer the exciting prospect of furnishing a potential solution to the urgent problem of the lack of circularity in the life cycle of plastics.^217,237^ **VU** vitrimers are reprocessable and even depolymerizable under milder conditions than polyurethanes and polyureas, which can be considered their conventional polymer analogues. As stated in Section 5, the **VU** motif is reminiscent of a carbamate, the difference being the presence of a conjugated double bond between the carbonyl group and the nitrogen atom (Figure 10). The similarity between the carbamate and urea skeletons allows one to extend the comparison with **VU**s to urea bonds as well.

**Figure 10:**
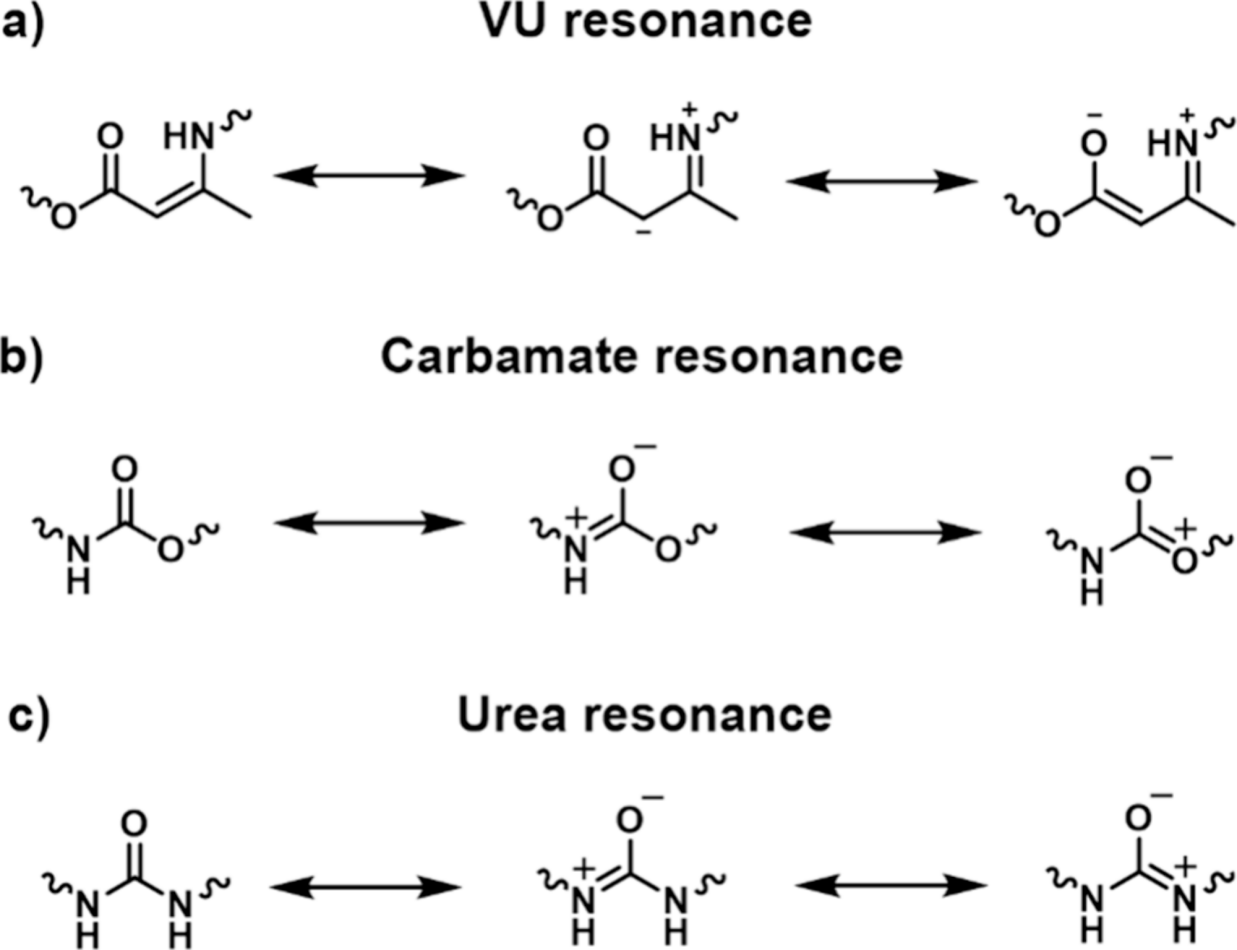
Resonance structures of (a) **VU**, (b) carbamate, and (c) urea moieties.

Polyurethanes and polyureas are highly inert polymers due to the stability of carbamates and ureas, which derive from the conjugation between the carbonyl group (C = O) and the lone electron pair of the nitrogen atom (Figures 10b–c).^237–239^ This stability is also reflected in the harsh conditions necessary to trigger the decomposition of these polymers, which requires high temperatures (above 160 °C) and Lewis acid catalysts.^240–243^ However, the dissociation of carbamates and ureas usually produces very reactive (and often toxic) isocyanate species that can undergo many side reactions, severely affecting the recovery of these building blocks.^244^ Other approaches to increase the chemical recyclability of polyurethanes and polyureas have focused on perturbing the conjugation of the carbamate and urea moieties by introducing bulky substituents on the nitrogen atom.^245–249^

The presence of the vinylic bond in the **VU** motif extends the conjugation of the system compared to carbamates and ureas (Figure 10a),^250^ and additionally facilitates chemical modifications. The vinylic bond and its adjacent amine (i.e., enamine) in the **VU** motif can be partially described as an iminium species (Figure 10a), and this species is susceptible to hydrolysis, which affords a β-ketoester and an amine.^53,146^ **VU** bonds thus offer an intriguing combination of chemical stability and degradability, and may offer a path to sustainable alternatives to polyurethanes and polyureas. On a less positive note, the extended conjugation described above imparts a coloration to the **VU**-containing polymers that may be undesirable, depending on the intended application.

Initial efforts in recycling **VU** vitrimers focused on the thermal reprocessing strategies based on transamination promoted by free amines reported by the Du Prez group in 2015,^52^ which have been already discussed in Section 5.1. This approach provides excellent results in reprocessing polymer films several times. However, the high temperatures necessary to activate the exchange of the **VU** bonds (above 120 °C) can lead to chemical degradation, such as oxidation of the free amine functionalities, which can further result in deteriorated physical properties and undesirable (dis)­coloration of the **VU** vitrimers.^110,251^ Moreover, thermal reprocessing may not be suitable for carbon fiber reinforced polymer composites, which are vastly applied as sporting goods, car components, and wind turbine blades, due to the inherent nonflowable nature of carbon fibers.^252–255^ To circumvent these limitations, several research groups developed chemical recycling methods relying on the depolymerization of **VU** vitrimers by treatment with small molecules carrying primary amine groups at temperatures between 60 and 120 °C, hence lower than those used for the thermal reprocessing (Figure 11, left).^88,116,220,256^ The working principle of these strategies is rooted in the Le Chatelier principle: the use of an excess of primary amine small molecules pushes the equilibrium toward the disassembled state of the **VU** network and the formation of new **VU** small molecules (Figure 11, left).^257^ Repolymerization with a fresh amine cross-linker, followed by the removal of the amine small molecules, reinstates the **VU** vitrimers.^88,116,220,256^ The proposed approaches were shown to be successful, although their execution poses questions related to their energy efficiency and atom economy.^54,258^

**Figure 11:**
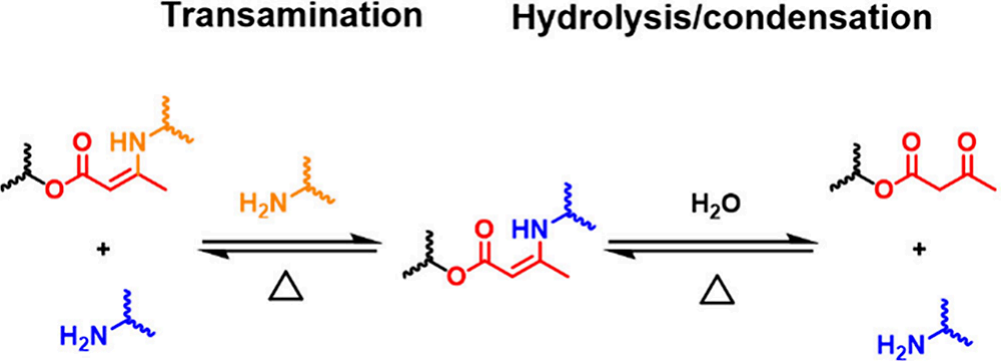
Transamination between an amine and a vinylogous urethane during thermal reprocessing or chemical recycling processes (left), and water-assisted dissociation of a vinylogous urethane in the presence or absence of acid into the parent β-ketoester and primary amine and its reverse condensation (right).

Berrocal, Shi, Weder, and co-workers recently reported a similar concept to promote the chemical recycling of **VU** vitrimers that was based on chemical hydrolysis.^108^ The strategy devised by the authors involved hydrolysis of the **VU** bonds (and hence depolymerization) using an excess of water, which allowed the recovery of the network constituents (Figure 11, right).^108^ Subsequent repolymerization of the recovered constituents (favored by water removal) led to closed-loop recycled **VU** vitrimers with excellent retention of the mechanical and thermal properties.^108^ The authors also explored the boundaries of the chemical hydrolysis with respect to the polarity of the polymer backbone comprising the **VU** linkages. Neutral water suffices to promote the hydrolysis of **VU** vitrimers comprising hydrophilic polyethylene glycol (**PEG**) as building block, and the rate of depolymerization can be readily controlled by the temperature, amount of water, and molecular weight of the **PEG** building block. However, the depolymerization in water is stifled if the more hydrophobic poly­(tetrahydrofuran) is employed to construct the skeleton of the **VU** vitrimers. In this case, a mixture of THF and acidic water is necessary to cause the depolymerization.^108^ These findings were later confirmed by the Chen lab, who reported the HCl-promoted closed-loop recycling of hydrophobic **VU** vitrimers synthesized from hexyl-substituted β-ketoesters (Chart 8) and **TREN**,^259^ and Zhou and co-workers, who investigated the influence of acidity on the depolymerization of vitrimers based on vinylogous carbamothioates (Chart 8). The latter are structurally similar to **VU**s in which the β-ketoester is replaced by a β-ketothioester.^260^ Overall, all these investigations have demonstrated that the depolymerization of **VU** vitrimers is readily feasible, can be carried out at room temperature, only requires the addition/removal of (acidic) water and optionally a cosolvent to swell the material, and promotes the closed-loop recycling of **VU** vitrimers.^261^

**Chart 8:**
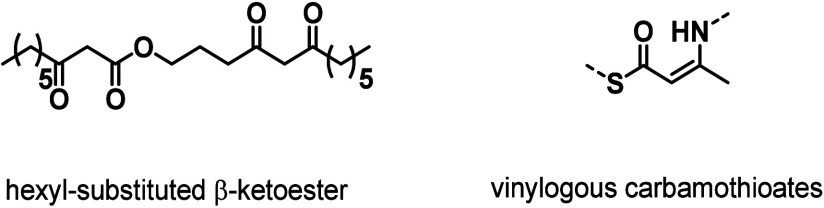
Chemical Structures of the Structural Components of the Polymers Discussed in Section 5.3

### 5.4 Click Strategies for the Synthesis of VU Polymer Networks

**VU** vitrimers are conventionally synthesized by polycondensation reactions of β-ketoesters and amines (Figure 12a). This process yields water as a byproduct, which often hinders the applicability of solution-cast films of **VU** vitrimers because of the presence of porous defects. Thus, alternative synthetic approaches have been developed.

**Figure 12:**
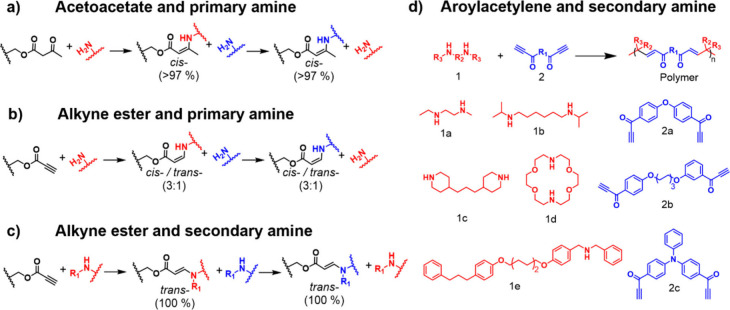
Synthesis of the **VU** motif via (a) amine–acetoacetate condensation, amino–yne click reaction with (b) primary or (c) secondary amines, and their respective exchange reactions between corresponding **VU** motifs and amines. (d) Synthesis of poly­(vinylogous urethane)­s (**PolyVU**s) via click polymerization of diamines and bis­(aroylacetylene)­s.

Du Prez and co-workers demonstrated the feasibility of amino–yne “click” polyadditions of alkyne esters and amines for the synthesis of **VU** vitrimers, which avoid the release of water as byproduct.^190^ This synthetic strategy was, however, marred by a side reaction that led to the formation of an amide bond, albeit the extent of this reaction was quantified as less than 5% and the properties of the thus-prepared, water-free **VU** vitrimers were comparable to those of **VU** vitrimers prepared from the polycondensation of structurally similar β-ketoesters and amines.^190^ In the same year, Gao, Shen, Lin, and co-workers also resorted to amino–yne click polyadditions of alkyne ester monomers devoid of a methyl substituent adjacent to the alkyne motif to synthesize **VU** vitrimers.^262^ This structural feature, i.e., the absence of the methyl substituent, affords **VU** motifs containing a lower fraction of *cis* isomers (67%) than generally obtained in conventional amine−β-ketoester polycondensations (97%) (Figures 12a-b). This difference is accompanied by a lower activation energy for bond exchange (35 kJ mol^–1^ vs 59 kJ mol^–1^, respectively). Nevertheless, the polymers exhibit superior mechanical properties and faster stress relaxation than conventional **VU** vitrimers. The same group expanded the scope of their methodology in a subsequent investigation, in which they explored the polyaddition of *secondary* amines with alkyne esters to prepare **VU** vitrimers containing sterically hindered **VU** motifs.^263^ The **VU** residues in the networks prepared by this approach were exclusively formed in the *trans* configuration (Figure 12c), and the authors could control the steric hindrance by selecting different types of secondary amines as starting materials. The activation energy for the bond exchange of the resulting **VU**s varied from 52 to 90 kJ mol^–1^ and followed the order piperidyl ∼ methyl < ethyl < isopropyl < *tert*-butyl. The activation energy for the stress relaxation behavior of the corresponding **VU** polymers followed a similar trend.

The disparities between the “click” polyaddition and polycondensation in the synthesis of **VU** vitrimers stem from the higher reactivity of the monomers in the former method. Indeed, polyadditions to synthesize **VU** vitrimers proceed at 0 °C, while polycondensations generally require temperatures between 50 and 80 °C. This higher reactivity is also manifested by the high conversions (up to 99%) obtained in reactions between secondary amines and alkyne esters,^264^ a process that is inherently not feasible at moderate temperatures when acetoacetates are employed as reactants. The low reactivity of acetoacetates with secondary amines has been strategically harnessed to impart creep resistance and high reprocessability to **VU** vitrimers by incorporating small quantities of triethylenetetramine (Figure 9b), as described in Section 5.2.^228^

The high reactivity of amino–yne click polyadditions also enables the use of diamines and bis­(aroylacetylene)­s to prepare high-molecular weight, linear **VU** polymers with high efficiency.^264,265^ Such polymers are chemically recyclable due to the presence of **VU** linkages in the backbone, holding promising prospects for the development of sustainable polymers, provided that properties comparable to those of currently nonrecyclable, commercial plastics could be reached and that the building blocks are economically viable. Along these lines, Svete and co-workers described the click polymerization of bis­[3-(dimethylamino)­acryloyl]­arenes and phenylenediamines to afford a novel poly­(vinylogous urethane) (**PolyVU**) with a molecular weight of ca. 3000 Da. The **PolyVU** thus prepared could be quantitatively depolymerized to its monomers by treatment with an excess of dimethylamine at 50 °C.^266^ Similarly, the Qin lab developed **PolyVU**s with molecular weights of up to 49 kDa via the amino–yne click polymerization of bis­(aroylacetylene)­s and diamines in the absence of catalysts (Figure 12d).^265^ A broad scope of bis­(aroylacetylene)­s and diamines was used as monomers in this study, affording polymers with modular backbones in high yields (up to 99%) (Figure 12d). The resulting **PolyVU**s could also be depolymerized by treatment with Lewis acids and monoamines.^265^ The same group later demonstrated the polymerization of bis­(ethynylsulfone)­s and various diamines into high-molecular-weight (up to 160 kDa) poly­(β-aminovinylsulfone)­s, and the subsequent high-yield depolymerization of these polymers via treatment with monofunctional amines. Overall, these investigations laid the foundations for the future development of high-performance, chemically recyclable **VU** polymers.

In summary, the amino–yne click reaction has significantly expanded the scope of amine compounds that can be employed in the preparation of **VU** polymers, encompassing aromatic and aliphatic, and primary and secondary amines. The large variety of employable amines allows the diversification of the pool of polymeric designs available and the fine-tuning of the dynamics and characteristics of the **VU** polymer (networks), thereby enhancing versatility and applicability.

## 6 Other Dynamic Polymer Networks Derived from β-Dicarbonyl Motifs

Besides their high reactivity toward amines, β-ketoester motifs have also been shown to undergo transformations into hydroxylesterenamides (also referred to as acetoacetyl formed amides) via nucleophilic addition of the α-methylene to an isocyanate (Figure 13a). This reaction had been extensively applied in the synthesis of coatings,^93,267^ but the dynamic nature of hydroxylesterenamides remained unexplored until Shi and co-workers used this unit to cross-link polybutadienes in 2019, establishing a new **DCN**.^142^ The resulting **DCN** could be reprocessed by compression molding over three cycles without discernible alterations to the chemical and mechanical properties of the materials. The authors postulated that the reversibility of the hydroxylesterenamide unit might derive from the thermal dissociation of the motif into ketene and amine.^142^ This proposed mechanism was subsequently confirmed by the Du Prez group, who experimentally observed the generation of ketenes by temperature-dependent FT-IR experiments.^76^ In this work, the authors leveraged hydroxylesterenamide linkages to prepare reprocessable polyurethane foams with a density as low as 32 kg/m^3^ from a hydrophobic terpene-based polyol, toluene diisocyanate, and 1,8-diazabicyclo[5.4.0]­undec-7-ene (**DBU**) (Chart 9).^76^ Strategies to circumvent the use of highly toxic and unstable isocyanates in the synthesis of hydroxylesterenamides are also under exploration. For instance, Liu et al. exploited the heat-triggered Curtius rearrangement of acyl azides into isocyanates.^268^ The authors used this chemistry to convert terephthaloyl diazides (Chart 9) into the corresponding diisocyanates in situ at 80 °C and subsequently react these species with castor oil acetoacetates in a one-pot process that afforded a **DCN** comprising dynamic hydroxylesterenamide cross-links.^268^ The resulting polymers are thermally healable and reprocessable at 130 °C. Moreover, the presence of a natural product such as castor oil provided biodegradability: the **DCN** degraded in soil within 24 weeks.^268^

**Figure 13:**
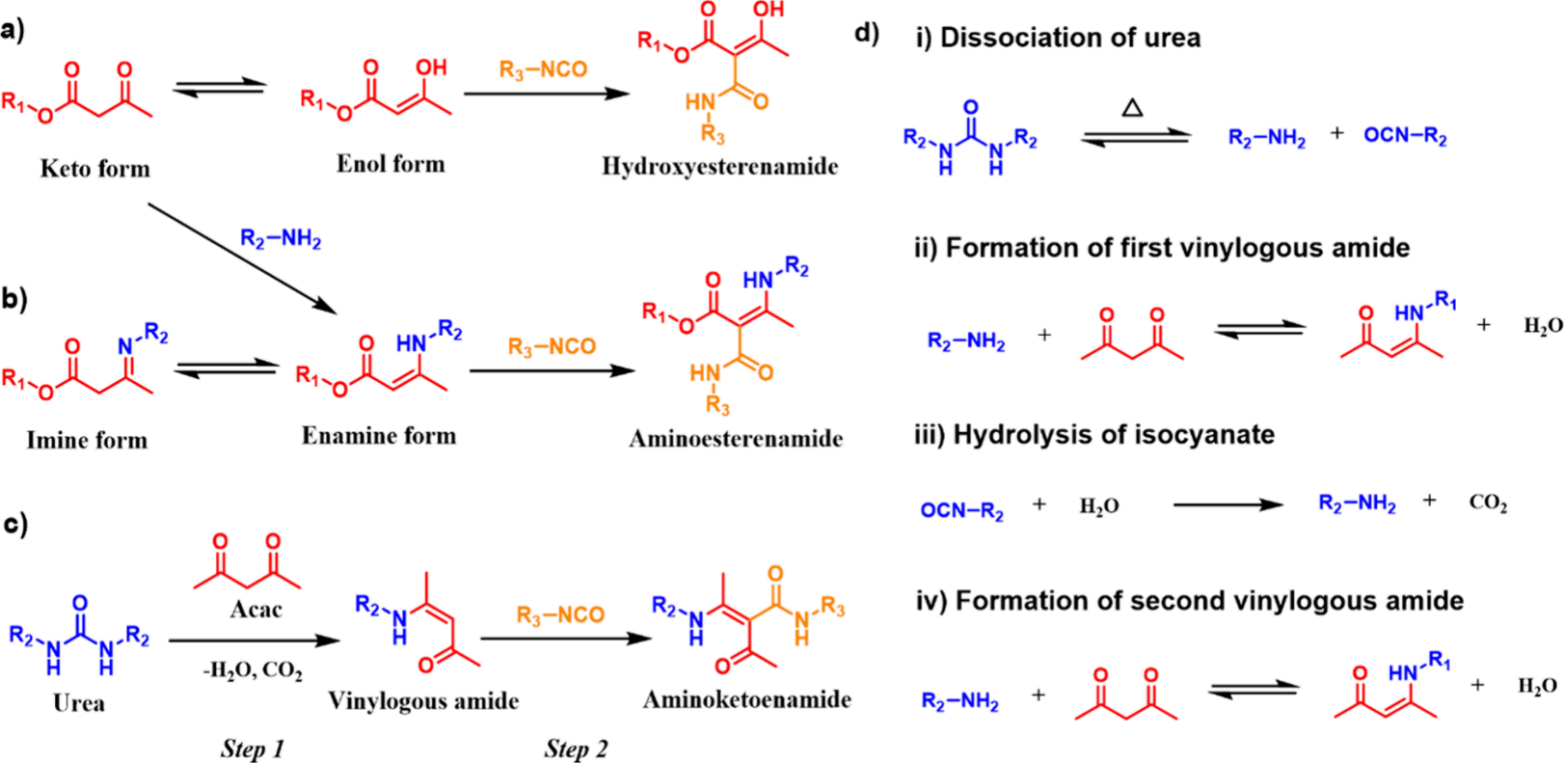
(a) Keto–enol tautomerism of β-ketoesters, and nucleophilic addition of β-ketoesters into isocyanates to afford hydroxylesterenamides. (b) Imine–enamine tautomerism of **VU**, and the nucleophilic addition of **VU** into isocyanates to afford aminoesterenamides. (c) Conversion of a conventional urea bond into a vinylogous amide by reaction with acetylacetone (**Acac**; *step 1*), followed by the subsequent transformation of the vinylogous amide into aminoketoenamide upon reaction with isocyanate (*step 2*). (d) Proposed reaction mechanism for the **Acac**-induced degradation of urea bonds into vinylogous amides (d).

**Chart 9:**
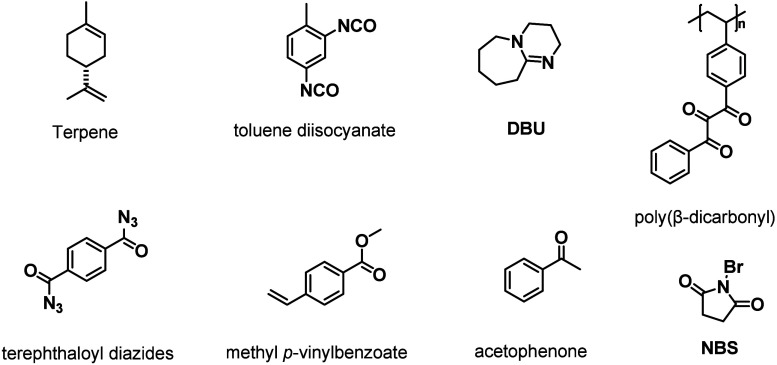
Chemical Structures of the Structural Components of the Polymers Discussed in Section 6

In addition to β-ketoesters, isocyanates can also react with **VU**s, affording aminoesterenamide linkages on account of the high nucleophilicity of **VU**s (cf. Section 1) in a process that was reported by Ma et al. (Figure 13b).^139^ The authors demonstrated that, upon heating, these linkages can dissociate into **VU**s and isocyanates, consequently facilitating the thermally induced repair and reprocessing of cross-linked poly­(aminoesterenamide) networks at 150 °C. The synthesis of aminoesterenamide involves three starting materials, namely β-ketoesters, isocyanates, and amines. This provides the opportunity to build macromolecular architectures with high modularity in a way that is analogous to small molecule multicomponent reactions, such as the Biginelli, Passerini, or Ugi reactions.^269^ Such high modularity of macromolecular design should also allow to customize the characteristics of the resulting **DCN**s to meet specific requirements.

Aminoesterenamides can also be prepared by the degradation of urea bonds. A collaboration between the Shi, Weder, and Berrocal groups revealed that simply heating polyureas with an excess of acetylacetone (**Acac**) affords vinylogous amides (*step 1*, Figure 13c).^140^ The authors proposed a reaction mechanism that encompasses four processes, namely *i*) the thermal dissociation of a urea moiety into an amine and an isocyanate, *ii*) the condensation of the amine with **Acac** to afford a vinylogous amide and water, *iii*) the hydrolysis of the isocyanate under formation of CO_2_ and a second amine, and *iv*) the condensation between the second amine and **Acac** to yield a vinylogous amide (Figure 13d). The vinylogous amides thus made could be subsequently transformed into dynamic aminoketoenamides upon reaction with isocyanates, exploiting the nucleophilicity of the formal α-methylene group (*step 2*, Figure 13c). The authors leveraged this framework to show that conventional polyureas could be chemically upcycled into dynamic covalent poly­(aminoketoenamide)­s that are thermally healable and reprocessable in the absence of any catalyst.^140^ In a follow-up study, it was shown that the controlled incorporation of substoichiometric amounts of aminoketoenamide linkages within poly­(urea–urethane)­s lowers the stress–relaxation of these materials, with activation energies ranging from 39 to 92 kJ mol^–1^.^141^

One limitation related to **DCN**s based on hydroxylesterenamides, aminoesterenamides, or aminoketoenamides arises from the highly reactive nature of the chemical species — ketenes and isocyanates — that are generated during the dynamic exchange of these **DCB**s.^270^ Both species are susceptible to hydrolysis, meaning that the preservation of material properties over multiple reprocessing cycles relies heavily on the experimental conditions: the reprocessing should be carried out in an inert atmosphere and avoid the presence of moisture.

The nucleophilicity of the α-methylene group has been extensively exploited by the Helms group with triketone derivatives.^54,271^ The triketones can react with primary aromatic or aliphatic amines, yielding diketoenamines and water as products. This chemistry allows the preparation of dynamic covalent poly­(diketoenamine)­s (**PDK**s) networks. In a seminal example, Helms and co-workers prepared triketone dimer **TK-6** (featuring a 6-carbon atom spacer between two triketone motifs), from the cyclic β-dicarbonyl compound dimedone and adipic acid.^54^ The subsequent cross-linking reaction of **TK-6** with **TREN** afforded poly­(diketoenamine) **PDK-6** networks (Figure 14a). The authors showed that **PDK-6** can be depolymerized using strong acids (5 M H_2_SO_4_), which provided starting **TK-6** and the protonated form of **TREN**. The recovery of the two building blocks was enabled by separation techniques that leveraged the chemical nature of the two species; **TK-6** was precipitated in acidic water and could be filtered off, while protonated **TREN** was recovered using a basic ion-exchange resin (Figure 14a).^54^ The authors also demonstrated that the acidic depolymerization/separation strategy is applicable in the case of mixed waste streams, and the presence of additives and fillers.

**Figure 14:**
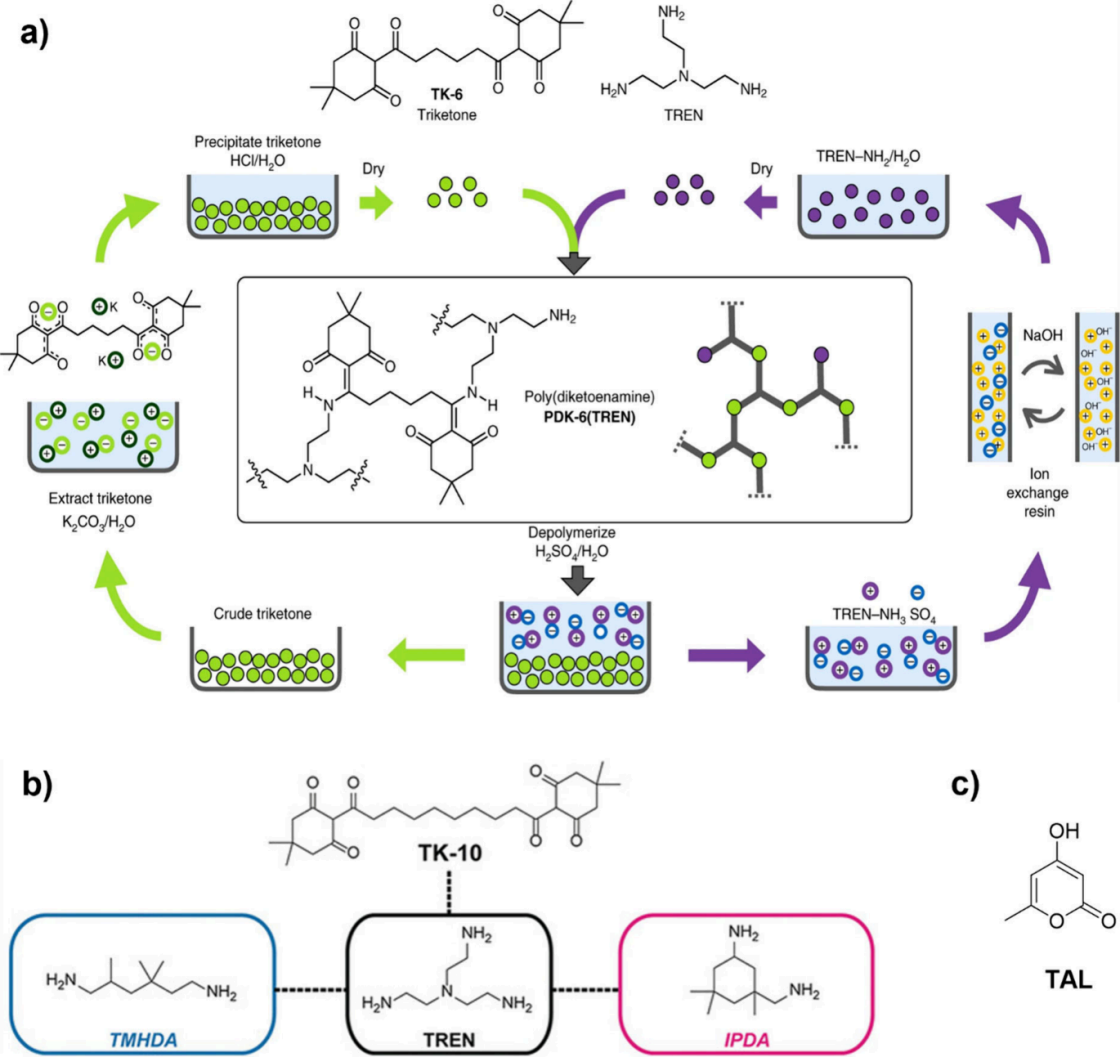
(a) Synthesis of poly­(diketoenamine) (**PDK-6**) via polycondensation of **TREN** and triketone (derived from adipic acid, **TK-6**), and hydrolysis of **PDK-6** in strong acid to recover **TREN** and **TK-6** using regenerative chemical processes. Adapted with permission from ref 54. Copyright 2019 Springer Nature. (b) Chemical structures of triketone dimer **TK-10**, trimethylhexamethylene diamine (**TMHDA**), isophorone diamine (**IPDA**), and **TREN**. Adapted with permission from ref 271. Copyright 2019 Wiley. (c) Chemical structure of triacetic acid lactone (**TAL**).

Following this work, the Helms group also investigated the influence of molecular weight and structural characteristics of polymer segments on the thermomechanical behavior of **PDK**s.^271^ More specifically, they reacted varying proportions of trimethylhexamethylene diamine (**TMHDA**) or isophorone diamine (**IPDA**) with triketone dimer **TK-10**, which is structurally similar to **TK-6** but features a 10-carbon atom spacer, and **TREN** (Figure 14b).^271^ **TMHDA** is conformationally flexible, while **IPDA** is much stiffer; consequently the nature of the diamine used in the polymer design significantly influences the viscoelastic properties of the **PDK**. **PDK**s featuring **TMHDA** display a lower activation energy for stress relaxation than those comprising **IPDA**. Additionally, controlling the feed of **TMHDA** and **IPDA** during synthesis allowed the control of the molecular weight of the prepolymers, which then modulated the cross-link density of the final **PDK** networks.^271^ The results indicate that the topology freezing temperature (*T*_v_) decreases upon increasing the flexibility of the linear segments (i.e., increasing the **TMHDA** content). By contrast, *T*_v_ increases upon increasing the molecular weight of the **IPDA**-bearing, rigid segments.

Following these first two studies,^54,271^ Helms and co-workers studied the consequences of (i) the heteroatom substitutions on the triketone monomers,^147^ (ii) the valency of the primary amine cross-linkers (bivalent, trivalent, and tetravalent amines),^233^ and (iii) the presence of a proximal tertiary amine in the design of the cross-linker and its distance from the cross-linking primary amines^272^ on the depolymerization of **PDK**s. The presence of electronegative heteroatoms, such as nitrogen and oxygen, in the triketone ring lowers the rate of depolymerization.^147^ Increasing the valency of the primary amines (e.g., tetravalent amines vs a mixture of bivalent and trivalent amines) appears to be essential for ensuring complete **PDK** deconstruction.^233^ Finally, the presence of a proximal tertiary amine in the cross-linker accelerates **PDK** depolymerization compared to similarly built cross-linkers that do not feature such a functionality.^272^ However, this effect rapidly decreases upon increasing the separation between the tertiary amine and the hydrolysis site (i.e., the diketoenamine linkages).^272^ The same group has recently used biobased “triacetic acid lactones” (**TAL**, Figure 14c), which are prepared from glucose through a fed-batch fermentation process and, thus, provide an alternative to the oil-derived dimedone monomer. In addition to the bioderived origin, **TAL** is particularly attractive because of its planarity, which allows the formation of stacks of **TAL** motifs that bestow **TAL**-based **PDK**s with high *T*_g_ values (>150 °C).^148^ Such high *T*_g_ values extend the applicability of **PDK**s to high-temperature environments. The Helms and Scown groups carried out a comprehensive assessment of the costs and life-cycle carbon footprints of both virgin and chemically recycled **PDK**s through systems analysis.^273^ The study revealed that the cost of recycling **PDK** resins ($1.5 kg^–1^) is comparable to that of polyethylene terephthalate and high-density polyethylene (∼ $1 kg^–1^), and lower than that of polyurethanes ($4.8 kg^–1^).^273^ The production of fresh **PDK** resins emitted 86 kg CO_2_ equivalents (CO_2_e) per kilogram of resin, while the chemical recycling produced only 2 kg CO_2_e kg^–1^.^273^

The examples discussed above^54,147,148,271–273^ relate to materials prepared from purpose-designed building blocks. However, the chemistry of **PDK**s has also been shown to be applicable in the context of the upcycling of polymer waste.^274^ A collaboration between the Helms and Leibfarth laboratories demonstrated that the triketone groups can be installed on the side chain of polyolefin waste via amidyl-radical-mediated C–H functionalization.^274^ Subsequent cross-linking with polytopic amines affords diketoenamine**-**based **DCN**s. The diketoenamines introduced microphase separation, and this results in improved mechanical properties, better creep–resistance, and higher thermal stability compared to the linear polyolefin counterparts due to the incorporation of the dynamic diketoenamine cross-links.^274^ Collectively, these efforts, spanning from structural design to economic evaluation, underscore the potential of **PDK**s as promising candidates for circular polymers, thereby contributing to the advancement of a circular plastics economy.

The nucleophilicity of the α-methylene group of β-dicarbonyls has been leveraged using aldehydes as electrophiles as well.^133^ A very recent study from Wang, Hadjichristidis, Li, and co-workers revealed that the reaction of β-ketoesters and benzaldehydes affords α-acetyl cinnamate, which can undergo exchange above 140 °C through an associative mechanism (Figure 15a, (i)).^275^ The authors incorporated α-acetyl cinnamate cross-links in poly­(meth)­acrylate networks that also featured β-ketoester moieties as pendant groups. Treating these networks at high temperatures (140 °C) promoted the chemical exchange between the α-acetyl cinnamate and β-ketoester groups, which ultimately led to vitrimeric behavior (Figure 15a, (i)). These materials exhibit high thermal stability, good creep resistance (Figure 15a, (ii)) at temperatures ≤ 120 °C, and display excellent reprocessability at 180 °C (Figure 15a, (iii)).^275^ This work demonstrated that high creep resistance and good thermal reprocessability are not mutually exclusive but can be engineered in vitrimers by rational design of dynamic motifs undergoing exchange at high temperatures.

**Figure 15:**
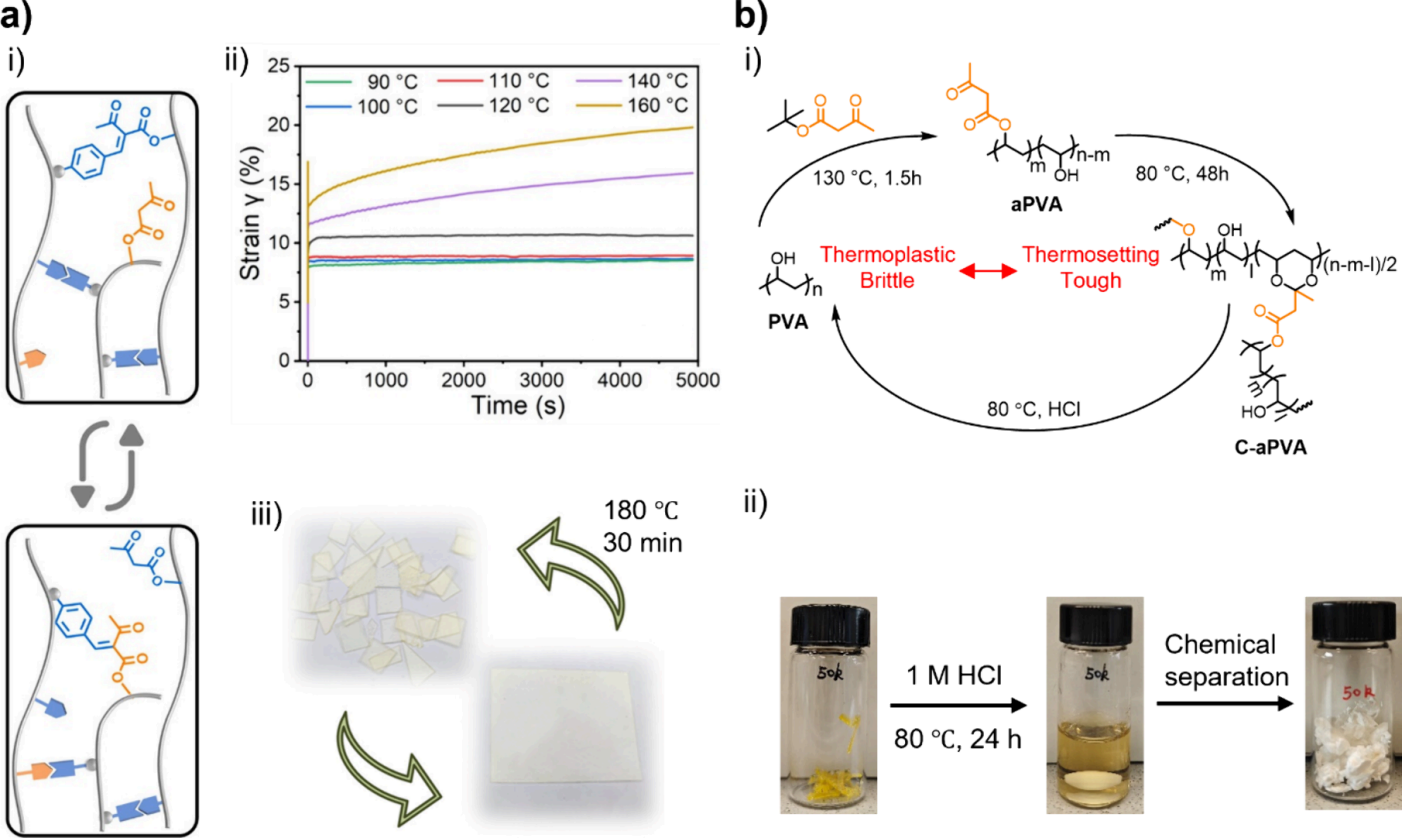
(a) (i) Topological network rearrangement assisted by a dynamic exchange between α-acetyl cinnamate and β-ketoester moieties; (ii) creep behavior of the α-acetyl cinnamate-cross-linked vitrimer at different temperatures; (iii) thermal reprocessing of α-acetyl cinnamate-cross-linked vitrimers at 180 °C for 30 min. Adapted with permission from ref 275. Copyright 2024 Wiley. (b) (i) Closed-loop recycling of **PVA** thermosets through the conversion of **PVA** into **PVA** acetoacetate (**aPVA**) with *tert*-butyl acetoacetate (**tBA**), subsequent transformation of **aPVA** into **DXE**-cross-linked **PVA** (**C-aPVA**) by reaction between the β-ketoester and 1,3-diol groups, and final deconstruction of **C-aPVA** into **PVA** at 80 °C in the presence of 1 M HCl; (ii) photographs illustrating the chemical processes shown in (i). Adapted with permission from ref 276. Copyright 2024 Wiley.

As discussed in Section 5, the carbonyl moieties of β-dicarbonyl derivatives are electrophilic sites that can react with amines and yield dynamic **VU** linkages.^52,53,108^ Expanding such reactivity to other nucleophiles, such as alcohols and thiols, would present a great opportunity to broaden the applicability of β-dicarbonyl chemistry and the scope of polymer substrates. However, mastering such reactions has, until recently, remained elusive, as β-ketoesters have been shown to be inert toward methanol and ethanol during the synthesis of dynamic **VU** motifs in which these alcohols were used as solvents.^52,88,224^ Ma, Stellacci, and co-workers have recently overcome this obstacle by reporting a system based on the reaction between β-ketoesters and 1,3-diols.^276^ The authors showed that this process proceeds in the absence of catalysts and affords β-(1,3-dioxane)­esters (**DXE**), which can dissociate into hydroxyls, acetone, and CO_2_ upon heating to 80 °C in an excess of aqueous HCl (Figure 15b, (i)).^276^ The authors demonstrated this chemistry by converting a fraction of the alcohol groups of poly­(vinyl alcohol) (**PVA**) — featuring only 1,3-diols along its backbone — into partially acetoacetate modified **PVA** (**aPVA**), and subsequently reacting the β-ketoesters of **aPVA** with the remaining, unmodified 1,3-diols of **aPVA** to form **DXE** cross-links. This sequence of transformations affords a new, cross-linked polymer (**C-aPVA)** (Figure 15b, (i)) whose Young’s modulus and toughness are increased by 2- and 11-fold compared to linear **PVA**. The authors also demonstrated that **C-aPVA** is degradable at 80 °C in an excess of 1 M aqueous HCl, which enabled an excellent recovery (>90%) of **PVA** (Figure 15b, (ii)).^276^

Several other interesting chemistries involving β-dicarbonyl systems have been reported. For example, β-dicarbonyl can undergo oxidation to form vicinal tricarbonyl (**VT**) derivatives when treated with *N*-bromosuccinimide (**NBS**, Chart 9) in DMSO.^277^ The central carbonyl group in the **VT** moiety is highly electrophilic because of its position adjacent to two electron-withdrawing carbonyl groups. As such, **VT** can react with a broad range of nucleophiles, including water,^277^ alcohols,^278–280^ thiols,^281^ and aromatic amines,^282^ affording the corresponding geminal diols, hemiacetals, hemithioacetals, and hemiaminals (Figure 16). The formation of these products is intrinsically reversible and they can dissociate back to **VT**, liberating the corresponding nucleophiles under appropriate conditions.^283^

**Figure 16:**
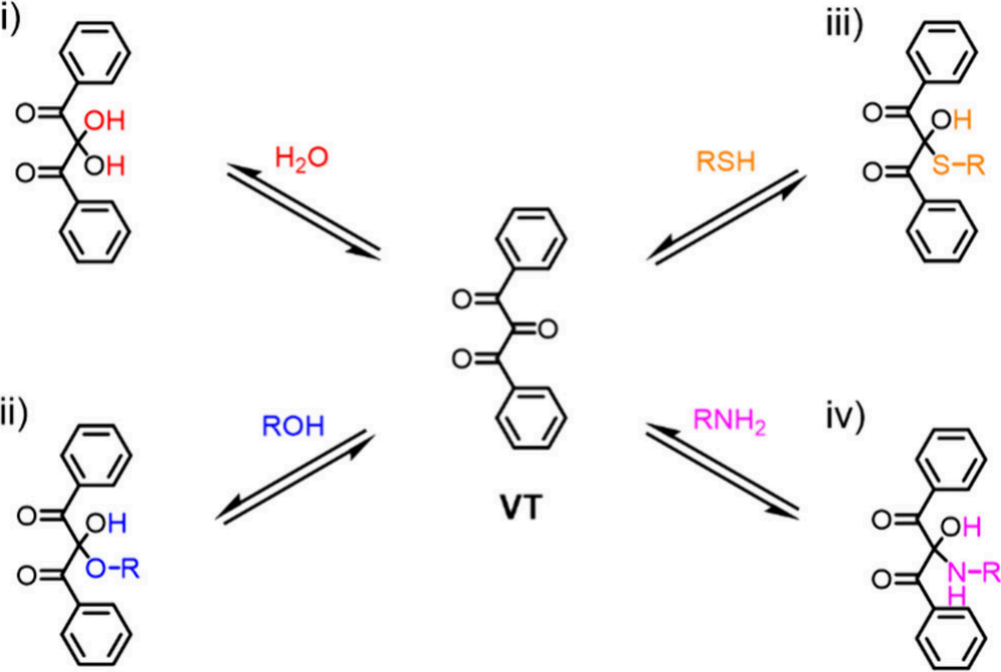
Reversible reactions of the vicinal tricarbonyl (**VT**) motif with water (i), or alcohol (ii), or thiol (iii), or amine (iv).

For example, Takeshi and co-workers initially synthesized a styrene derivative bearing the β-dicarbonyl unit via Claisen condensation of methyl *p*-vinylbenzoate and acetophenone (Chart 9).^278^ This compound was then subjected to free radical polymerization to give a poly­(β-dicarbonyl) (Chart 9). The oxidation of poly­(β-dicarbonyl) with **NBS** transforms the β-dicarbonyl moieties into **VT**s, which allows the preparation of **DCN**s upon cross-linking with 1,6-hexanediol. Heating the **DCN**s to 50 °C in the presence of water triggered the decross-linking process and allowed the recovery of the **VT**-containing polymer with a yield of 96%.^278^

## 7 Applications of Dynamic Polymers Derived from β-Dicarbonyl Motifs

Among the numerous dynamic chemistries based on the β-dicarbonyl motifs, **VU** chemistry is the most representative and extensively investigated. The building blocks to access a broad range of **VU** derivatives are either commercial or easily accessible, and the dynamic nature of **VU** linkages, together with the high compatibility of these bonds with a number of postfunctionalization^87,284–286^ and polymerization techniques, make **VU** chemistry a promising platform for (new) chemistry and functions.^69,74,75,111,222,287,288^ The general applicability of **VU** polymers can be further expanded if functional starting materials and composite fillers are incorporated into the polymer (networks). Selected examples include ion transport,^71,289^ shape memory behavior,^290–293^ light-responsive character,^69,74,75,111,222,287,288^ antibacterial functions,^113,114,294–297^ antiflame properties,^298,299^ and drug delivery,^74,75,300,301^ among others.

Many natural or synthetic polymers, including cellulose,^114,285,286,294,296^ polymethacrylates,^284^ and fluorinated polymers^87^ have been modified through the incorporation of dynamic **VU** motifs, allowing one to obtain new polymer properties and characteristics. The Sui lab developed a cellulose sponge featuring β-ketoester groups that could react with various functional amines.^286^ The microstructures and mechanical properties of the sponge were retained after the modification process: the resulting **VU** bonds could be dissociated by treatment with acid, and reformed under mild, neutral conditions.^286^ The surface of the cellulose sponge could switch reversibly between hydrophilic and hydrophobic nature as a function of pH on account of the pH-responsiveness of the **VU**–amine ensemble. This characteristic could be applied to achieve efficient oil–water separation.^285^ Du Prez, Bowman, and co-workers demonstrated that **VU** chemistry can be exploited to decorate the surface of polymers with compounds that would be otherwise incompatible, and even laminate two incompatible films to form a tight bond.^87^ The authors first synthesized two intrinsically incompatible **VU** vitrimers comprising polypropylene glycol (**PPG**) or perfluorinated polyether (**PFPE**, Chart 10) skeletons by cross-linking bisacetoacetate-modified **PPG** and **PFPE** with **TREN**. Compression molding of these two polymer films at 150 °C and pressure of 2 tons for 20 min afforded a new, laminated film in which the two layers could not be peeled off without damaging the other layer, indicating strong adhesive forces brought by the formation of new **VU** linkages at the interface.^87^ In a separate demonstration, the authors incorporated a drop of **PFPE** bisacetoacetates onto the surface of the **PPG**-based **VU** vitrimer by heating the mixture to 120 °C for 10 min and showed that the contact angle of the modified **VU** vitrimer increased from 107 to 117°. This finding presents a promising strategy for direct surface modification or assembly of incompatible matrices, which usually relies on the use of adhesives.

**Chart 10:**
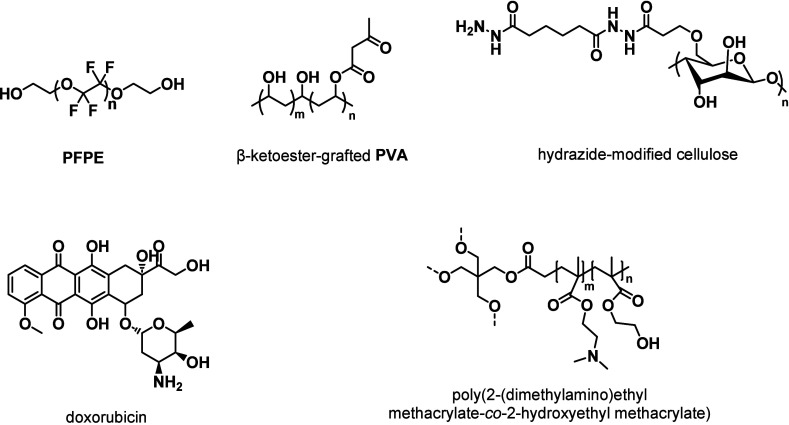
Chemical Structures of the Structural Components of the Polymers Discussed in Section 7

Incorporating functional compounds or composite fillers into **VU** vitrimers often allows one to impart the material with functions and properties that are characteristic of the incorporated species.^71,289,291,297,298^ This strategy offers the possibility to expand the applicability of **VU** vitrimers and obtain properties beyond reach for neat **VU** polymers. On the other hand, as discussed in the previous sections, engineering materials with **VU** bonds allows the extension of the materials’ lifetime via thermal healing, reprocessing, and/or chemical recycling strategies. Bao and co-workers developed a novel solid polymer electrolyte by incorporating lithium bis­(fluorosulfonyl)­imide (**LiFSI**) into poly­(ethylene oxide) (**PEO**)-based **VU** vitrimers (Figure 17a, (i)).^71^ The addition of **LiFSI** accelerated the stress relaxation rates of these materials, improved their reprocessability (Figure 17a, (ii)), and provided moderate ion conductivity (∼10^–5^ S cm^–1^ at room temperature) (Figure 17a, (iii)).^71^ Evans and co-workers later revealed that the presence of the **LiFSI** salt decreases the relaxation time (and thus increases the relaxation rate) in the viscoelastic measurements by a factor of ∼ 70 relative to the neat **PEO**-based **VU** vitrimers, since **Li** ^**+**^ ions coordinate to **VU** bonds and further catalyze their exchange.^289^ However, increasing the density of cross-links decreased the ionic conductivity of the vitrimer electrolytes due to the restricted chain movement.^289^ Gu and co-workers reported a linear polyacrylate resin with pendant sulfobetaine and β-ketoester groups, which could be cross-linked by polysiloxane-based multiamines (**HPSi**) to afford **VU** vitrimers (Figure 17b, (i)).^297^ The extent of incorporation of **HPSi** in the vitrimers was systematically controlled to tailor their mechanical, thermal, and self-healing properties. Furthermore, the vitrimers exhibited antibacterial rates above 95% against *E. coli* and *S. aureus* thanks to the presence of sulfobetaine groups (Figure 17b, (ii)).^297^ In another work, Wurm and co-workers showed that the incorporation of phosphonate groups into the backbone of **VU** vitrimers provided the materials with flame-retardant abilities comparable to commercial, flame-retardant resins, and most importantly, with reprocessability.^298^

**Figure 17:**
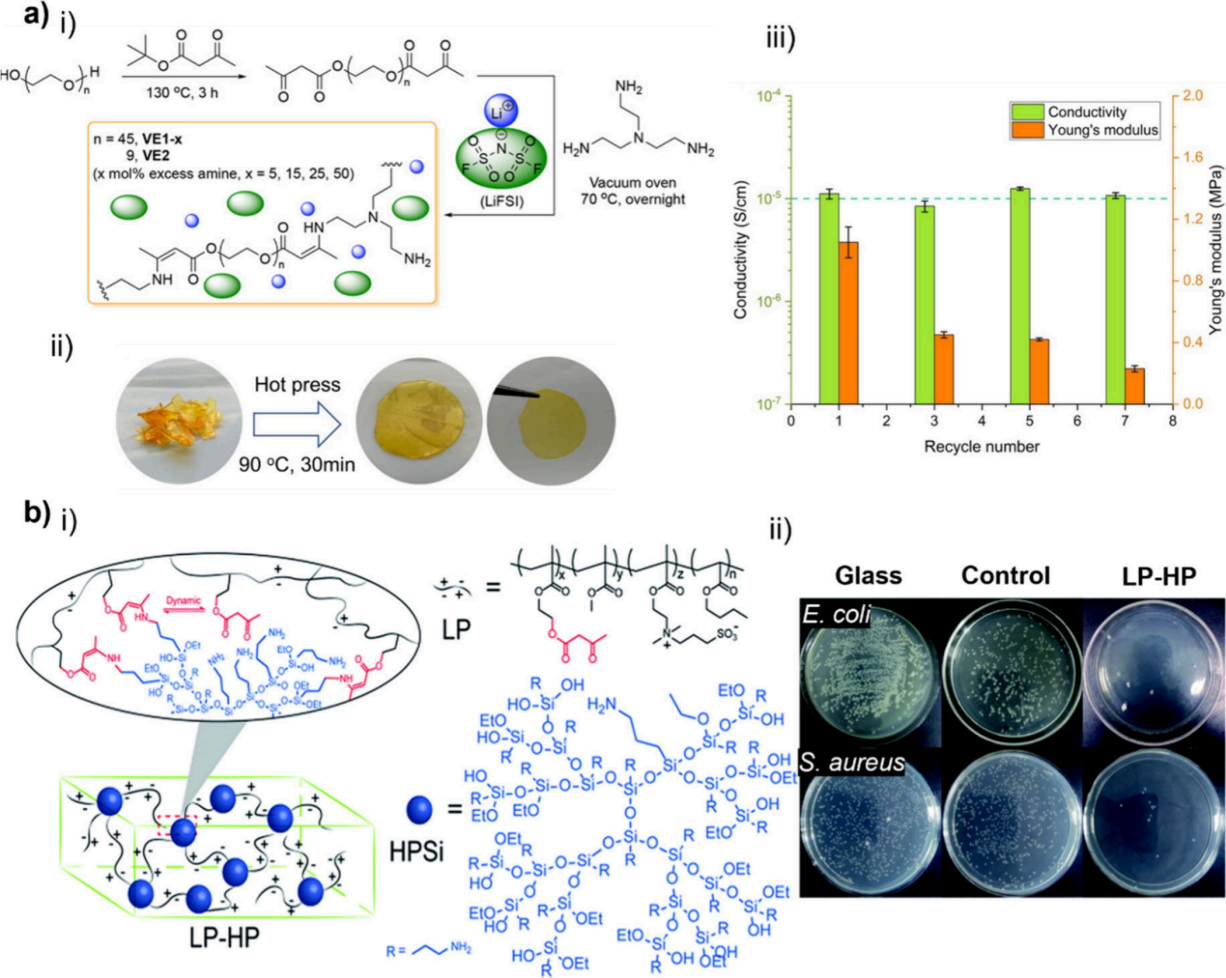
(a). (i) Preparation of **PEO**-based **VU** vitrimer electrolyte; (ii) thermal reprocessing of the shreds of the **PEO**-based **VU** vitrimer into a homogeneous film via compression molding at 90 °C for 30 min; (iii) ionic conductivity and Young’s modulus of the **PEO**-based **VU** vitrimer electrolyte after being reprocessed seven times. Adapted with permission from ref 71. Copyright 2022 the American Chemical Society. (b) (i) Chemical structures of polyacrylate resin (**LP**) bearing the sulfobetaine and β-ketoester groups, a multiamine hyperbranched polysiloxane (**HPSi**), and a polyacrylate-based **VU** vitrimer (**LP-HP**); (ii) antibacterial activities of glass, control sample (without the sulfobetaine motif), and **LP-HP** against *E. coli* (top) and *S. aureus* (bottom) after 3 h of contact. Adapted with permission from ref 297. Copyright 2017 the Royal Society of Chemistry.

The dissociative nature of **VU** polymers makes them also promising candidates for drug delivery, particularly in the form of hydrogel formulations.^74,75,300,301^ Lu and co-workers prepared biocompatible hydrogels with tunable mechanical properties, injectability, self-healing behavior, and pH-responsiveness by cross-linking β-ketoester-grafted **PVA** with hydrazide-modified cellulose (Chart 10).^301^ The hydrogels display a self-healing efficiency of 98% after keeping the fractured surfaces in contact for 2 h in a physiological environment. Importantly, they display pH-controlled release of pharmacologically active doxorubicin (Chart 10), on account of the reversibility of the amide-modified **VU** bonds. Moreover, the materials could be internalized by cells via 3D encapsulation without altering cell viability. In another investigation, Yuan and co-workers first synthesized four-armed star-shaped poly­(2-(dimethylamino)­ethyl methacrylate-*co*-2-hydroxyethyl methacrylate) (Chart 10) by ATRP and then carried out the transesterification of the pendant hydroxyl groups with **tBA** to install β-ketoester moieties.^75^ The β-ketoester-modified copolymer thus made was cross-linked by poly­(ether imide) and polydopamine to afford a thermoresponsive nanocomposite hydrogel. This material was subsequently loaded with doxorubicin, a chemotherapeutic agent, injected into the tumor of a mouse, and the near-infrared (NIR)-stimulated, controlled release of the drug was demonstrated. The authors also reported that this approach effectively killed the cancer cells and reduced the side effects of the drug.^75^

As discussed in Section 4, metal−β-dicarbonyl coordination polymers can be designed to integrate both intrinsic and dynamic properties derived from the simultaneous presence of the metal ion and the β-dicarbonyl moieties. These features are attractive for the use of metal−β-dicarbonyl coordination polymers as sensors,^185^ catalysts,^67,302^ and LEDs,^303–307^ to name a few. Muzafarov and co-workers synthesized a **PDMS** comprising β-diketone side groups (Figure 18a, (i)) that formed complexes with Eu^3+^ ions, which resulted in red fluorescence upon irradiation with 365 nm light (Figure 18a, (ii)).^176^ When the luminescent polymer was exposed to ammonia vapors (ammonia is a competitive ligand for Eu^3**+**^), the fluorescence intensity displayed a 1.5-fold decrease after 45 min (Figure 18a, (iii)). Removal of ammonia by vacuum reinstated the original fluorescence intensity (Figure 18a, (iii)).^176^

**Figure 18:**
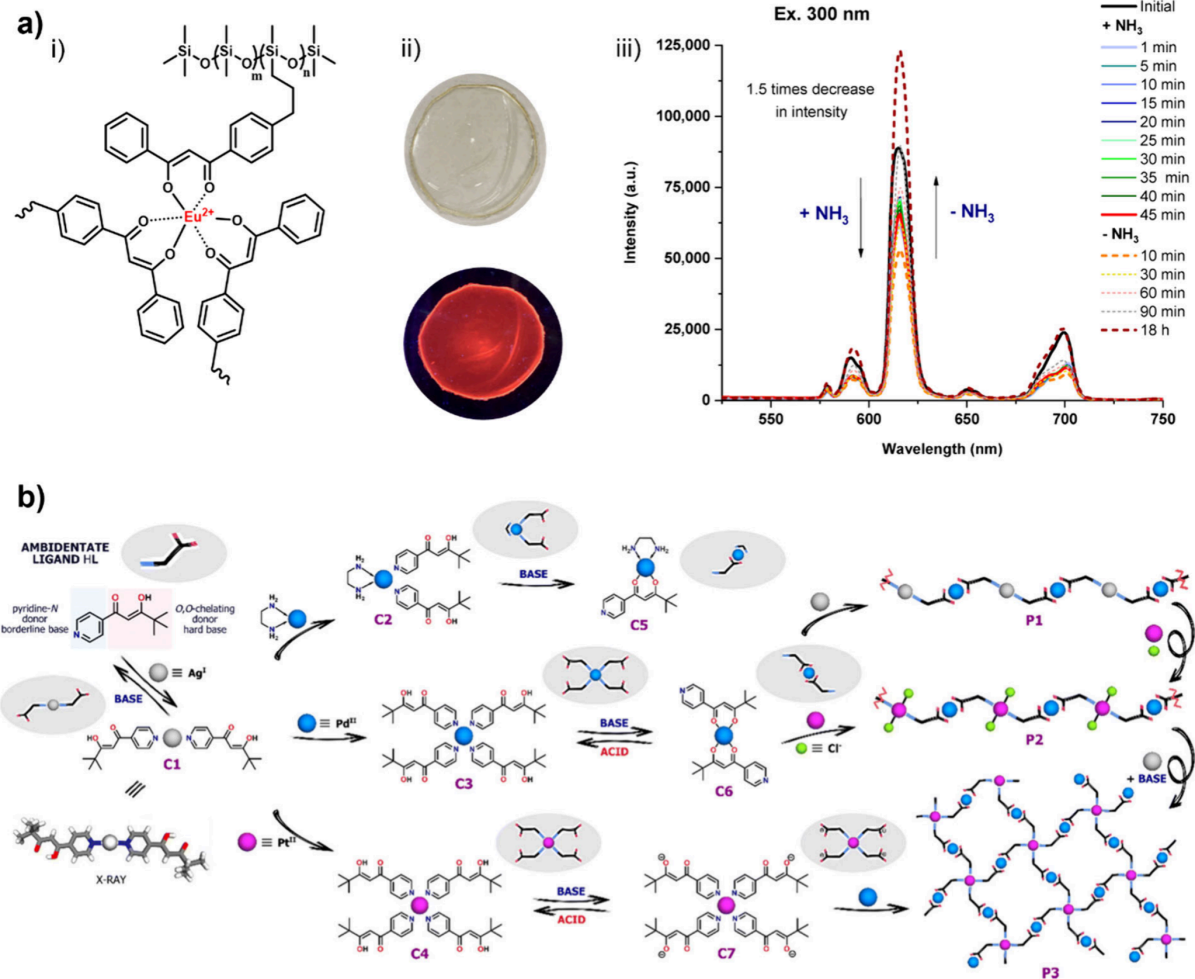
(a) (i) Chemical structure of Eu^3+^–β-diketone complexes on the side chain of **PDMS** (i); (ii) photographs of the thus made **PDMS** material exposed to ambient light (top) and to 365 nm light (bottom); (iii) fluorescence of the **PDMS**–material in the presence and absence of ammonia, measured as a function of time (λ_ex_ = 300 nm). Reproduced with permission from ref 176. Copyright 2022 MDPI. (b) Coordination-driven assemblies based on bifunctional ligand 4,4-dimethyl-1-(pyridin-4-yl)­pentane-1,3-dione and Ag^+^, Pd^2+^ and Pt^2+^ ions. Reproduced with permission from ref 308. Copyright 2023 Royal Society of Chemistry.

Stefankiewicz and co-workers demonstrated that the strong coordination of precious metal ions, including Ag^+^, Pd^2+^, and Pt^2+^, by β-diketonate species can be used to assemble small-molecule complexes into heterometallic polymeric materials.^308^ The authors synthesized 4,4-dimethyl-1-(pyridin-4-yl)­pentane-1,3-dione (**HL**), a bifunctional ligand comprising a β-diketonate and a pyridine moiety (Figure 18b, left). Different architectures of complexes in either small-molecular or macromolecular form were obtained by manipulation of the coordination modes of these two motifs. Moreover, the architectures could be switched through the nature of the metal salts or by controlling acid–base equilibria (Figure 18b, middle). The authors demonstrated that a metallo-supramolecular polymer was obtained in a yield of 74% upon sequential addition of Pt^2+^ and Pd^2+^ ions (Figure 18b, right), and that the supramolecular polymer thus made is able to catalyze the Heck cross-coupling reaction between iodobenzene and styrene thanks to the presence of the metal.^308^

In comparison to **VU** vitrimers and metal−β-dicarbonyl coordination polymers, the investigation on the applications of dynamic polymers leveraging other exchangeable linkages derived from the β-dicarbonyl synthon, such as diketoenamines and enamide derivatives, remains largely unexplored. Most of the research efforts in this context have been focused on establishing structure–property relationships on exchange dynamics and controlling polymers’ reprocessability or depolymerization.^108,141,143,147,233^ We foresee that this wealth of fundamental knowledge is only anticipating future work on the exploration of potential applications of polymer materials resorting to such linkages.

## 8 Summary and Outlook

Dynamic polymers incorporating reversible covalent bonds in their backbone or as cross-links represent a paradigm shift for the future of polymer and materials science. Among these dynamic polymers, those derived from β-dicarbonyl chemistry have garnered considerable attention due to their versatility, ease of synthesis, and wide availability of commercially viable starting materials. This review has summarized the rapidly emerging developments achieved in this field, providing an overview of (i) general approaches for the incorporation of β-dicarbonyl motifs into macromolecules, (ii) progress in metal−β-dicarbonyl coordination polymers, (iii) development of vinylogous urethane polymer networks, and (iv) development of other dynamic covalent networks based on dynamic linkages derived from the β-dicarbonyl chemistry, such as hydroxylketoenamide, aminoketoenamide, and diketoenamine.

Although researchers have pushed the boundaries of polymers leveraging β-dicarbonyl chemistry a great deal, we propose several key topics that could be explored in future research to foster industrial applicability. The ability of β-dicarbonyl motifs to coordinate metal ions presents a promising avenue for the development of novel metallo-supramolecular polymers. The dynamic nature of the coordination bonds, in conjunction with the intrinsic properties of the metal–ligand complexes, can spark the creation of multifunctional supramolecular polymers suitable for diverse optical, electric, magnetic, or catalytic applications. On the other hand, the integration of the two has been little explored and could lead to a dramatic expansion of the arsenal of functionalities and applications of β-dicarbonyl polymers. Exquisite knowledge of the chemical, physical, and material features of these metallo-supramolecular polymers is pivotal to reaching this goal, which calls for interdisciplinary collaborations among different communities.

β-Dicarbonyl motifs have shown to be reactive to amines, isocyanates, aldehydes, and 1,3-diols to afford the corresponding dynamic covalent bonds. These motifs can also react with other electrophilic or nucleophilic moieties, such as alkenes, alkynes, and thiols,^133^ but investigations of the properties of the resulting motifs are scarce. We postulate that some of the bonds formed from the reaction between β-dicarbonyl motifs and some of these functionalities may be reversible under appropriate conditions, such as high temperature or in the presence of catalysts, on account of their conjugation, which is reminiscent of that of **VU**. Possibly, exploring new electrophiles/nucleophiles can be beneficial in modulating the optical characteristics of the β-dicarbonyl-derived **DCB**s. This may be relevant for packaging applications, for example, for which some of these current **DCB**s do not possess optimal coloration. All in all, the rapid establishment of **VU** polymers strongly suggests that not only would such an endeavor be interesting from a fundamental chemistry standpoint, but it could also lead to new, technologically relevant polymer materials.

The development of closed-loop or chemically recyclable polymers offers a solution to the urgent issues associated with the life cycle of plastic products and the management of (micro)­plastic waste. Promising approaches to chemically recyclable polymers have been reported, resorting to β-dicarbonyl motifs and their derivatives, yet the impression is that we have only scratched the surface, and more scalable and cost-competitive approaches need to be explored. The field has just started to explore the (closed-loop) recycling of **VU** polymers, while recyclable polymer networks comprising diketoenamine and enamide linkages are still in their infancy. It is our opinion that the valuable experience and knowledge acquired from **VU** polymers could be a useful tutorial for recyclable networks based on other β-dicarbonyl derivatives. The high modularity and tunable reactivity of the β-dicarbonyl skeleton can be further explored to provide other types of recyclable polymers, which is an exciting opportunity for future research.

The transformation of synthetic or natural polymers into high-performance, reprocessable or recyclable polymers via a straightforward chemical modification (often a transesterification) is an intriguing topic to explore more in detail. Polymers equipped with β-dicarbonyl motifs can be readily cross-linked into dynamic covalent polymers by simple treatment with amines or isocyanates. Conceptually, this is an almost universal approach to transform any linear polymer into (recyclable) cross-linked architectures that could lead to strategies for upcycling plastic waste or repurposing polymers without developing brand-new recyclable polymers from scratch.

The next topic of discussion is related to engineering dynamic covalent polymers derived from the β-dicarbonyl chemistry with fast (re)­processing characteristics. The formidable challenge here is to enhance the dynamics of exchange between dynamic bonds, which will decrease the flow viscosity of the dynamic material, without enhancing the materials’ tendency to creep. A few approaches have been reported to boost exchange dynamics, such as incorporating pendant amines in close proximity to vinylogous urethane bonds, or leveraging the addition of catalysts, neighboring group participation, and incorporation of dual-dynamic covalent bonds. Interesting strategies to make creep-resistant, yet fast-reprocessing vinylogous urethane vitrimers by controlling the interplay between the associative exchange of vinylogous urethane linkages and the dissociative exchanges of dicarboxamide bonds^226^ and β-amino esters have recently been introduced.^227^ We anticipate that strategies resorting to the preparation of interpenetrated polymer networks, or controlling network morphology via microphase-separation or crystallinity, which have been employed in other vitrimer systems,^195^ may be helpful in reaching the challenging trade-off between creep resistance and fast reprocessing.

Last but not least, dynamic polymers derived from the β-dicarbonyl chemistry offer the intriguing prospect of possibly replacing conventional and more challenging-to-recycle polyurethanes, polyureas, and perhaps even epoxy resins. This possible transformation will certainly be governed by economic considerations, i.e., sustainable polymers leveraging β-dicarbonyl chemistry have to be economically more viable than current commercially available alternatives to become a technological reality. While the costs associated with (large-scale) production (e.g., starting materials, solvents, temperature, and general energy input) certainly play an important role in the equation, life-cycle assessment quantifications are also key parameters to consider. Concerning this aspect, it appears that dynamic polymers have the incredible advantage of providing access to practically infinitely, efficiently, and performance-retaining recyclable materials.

## Abbreviations

AEMA: (2-acetoacetoxy)­ethyl methacrylate
AIBN: azobis­(isobutyronitrile)
AAe: acryloylacetone
ATRP: atom transfer radical polymerization
Acac: acetylacetone
aPVA: PVA acetoacetate
BIS: *N*,*N*′-methylenebis­(acrylamide)
BMA: butyl methacrylate
Cur: curcumin
DBs: dynamic bonds
DCBs: dynamic covalent bonds
DCNs: dynamic covalent networks
DFT: density functional theory
DXE: β-(1,3-dioxane)­ester
EAA: ethyl acryloylacetate
FT-IR: Fourier transform infrared spectroscopy
HPSi: polysiloxane-based multiamine
HL: 4,4-dimethyl-1-(pyridin-4-yl)­pentane-1,3-dione
IPDI: isophorone diisocyanate
IPDA: isophorone diamine
LEDs: light-emitting diodes
LiFSI: bis­(fluorosulfonyl)­imide
MXDA: *m*-xylylenediamine
NMRP: nitroxide-mediated free radical polymerization
nBA: *n*-butyl acrylate
NBS: *N*-bromosuccinimide
PVA: poly­(vinyl alcohol)
PEG: poly­(ethylene glycol)
PMAAs: poly­(methacrylic acid)­s
PVAA: β-ketoester-modified poly­(vinyl alcohol)
PAM: polyacrylamide
PACM: 4,4-diaminocyclohexylmethane
*p*TsOH: *p*-toluenesulfonic acid
PolyVU: poly­(vinylogous urethane)
PDK-6: poly­(diketoenamine)
PPG: polypropylene glycol
PFPE: perfluorinated polyether
PEO: poly­(ethylene oxide)
RAFT: reversible addition–fragmentation chain transfer
ROMP: ring-opening metathesis polymerization
RDRP: reversible deactivation radical polymerization
tBA: *tert*-butyl acetoacetate
TREN: tris­(2-aminoethyl)­amine
TMHDA: trimethylhexamethylene diamine
TAL: triacetic acid lactone
VUs: vinylogous urethanes
VT: vicinal tricarbonyl

## References

[1] Armon S., Efrati E., Kupferman R., Sharon E. (2011). Geometry and Mechanics
in the Opening of Chiral Seed Pods. Science.

[2] Elbaum R., Zaltzman L., Burgert I., Fratzl P. (2007). The Role of Wheat Awns
in the Seed Dispersal Unit. Science.

[3] Forterre Y., Skotheim J. M., Dumais J., Mahadevan L. (2005). How the Venus
Flytrap Snaps. Nature.

[4] Sun J.-Y., Zhao X., Illeperuma W. R. K., Chaudhuri O., Oh K. H., Mooney D. J., Suo Z. (2012). Highly Stretchable
and Tough Hydrogels. Nature.

[5] Feng H., Zheng N., Peng W., Ni C., Song H., Zhao Q., Xie T. (2022). Upcycling of Dynamic
Thiourea Thermoset
Polymers by Intrinsic Chemical Strengthening. Nat. Commun..

[6] Burnworth M., Tang L., Kumpfer J. R., Duncan A. J., Beyer F. L., Fiore G. L., Rowan S. J., Weder C. (2011). Optically Healable
Supramolecular Polymers. Nature.

[7] Li C. H., Wang C., Keplinger C., Zuo J. L., Jin L., Sun Y., Zheng P., Cao Y., Lissel F., Linder C., You X. Z., Bao Z. (2016). A Highly Stretchable
Autonomous Self-Healing
Elastomer. Nat. Chem..

[8] Davis D. A., Hamilton A., Yang J., Cremar L. D., Van Gough D., Potisek S. L., Ong M. T., Braun P. V., Martínez T. J., White S. R., Moore J. S., Sottos N. R. (2009). Force-Induced Activation
of Covalent Bonds in Mechanoresponsive Polymeric Materials. Nature.

[9] Hemmer J. R., Rader C., Wilts B. D., Weder C., Berrocal J. A. (2021). Heterolytic
Bond Cleavage in a Scissile Triarylmethane Mechanophore. J. Am. Chem. Soc..

[10] Sagara Y., Karman M., Verde-Sesto E., Matsuo K., Kim Y., Tamaoki N., Weder C. (2018). Rotaxanes
as Mechanochromic Fluorescent
Force Transducers in Polymers. J. Am. Chem.
Soc..

[11] Hemmer J. R., Bauernfeind V., Rader C., Petroselli M., Weder C., Berrocal J. A. (2023). Triarylmethane Mechanophores Enable
Full-Visible Spectrum Mechanochromism. Macromolecules.

[12] Wu B., Lu H., Le X., Lu W., Zhang J., Theato P., Chen T. (2021). Recent Progress
in the Shape Deformation of Polymeric Hydrogels from
Memory to Actuation. Chem. Sci..

[13] Miao W., Zou W., Jin B., Ni C., Zheng N., Zhao Q., Xie T. (2020). On Demand Shape Memory
Polymer via Light Regulated Topological Defects
in a Dynamic Covalent Network. Nat. Commun..

[14] Mura S., Nicolas J., Couvreur P. (2013). Stimuli-Responsive
Nanocarriers for
Drug Delivery. Nat. Mater..

[15] Hu S., Pei X., Duan L., Zhu Z., Liu Y., Chen J., Chen T., Ji P., Wan Q., Wang J. (2021). A Mussel-Inspired
Film for Adhesion to Wet Buccal Tissue and Efficient Buccal Drug Delivery. Nat. Commun..

[16] Montero
De Espinosa L., Meesorn W., Moatsou D., Weder C. (2017). Bioinspired
Polymer Systems with Stimuli-Responsive Mechanical Properties. Chem. Rev..

[17] Wojtecki R. J., Meador M. A., Rowan S. J. (2011). Using the
Dynamic Bond to Access
Macroscopically Responsive Structurally Dynamic Polymers. Nat. Mater..

[18] Zou W., Dong J., Luo Y., Zhao Q., Xie T. (2017). Dynamic Covalent
Polymer Networks: From Old Chemistry to Modern Day Innovations. Adv. Mater..

[19] Rowan S. J., Cantrill S. J., Cousins G. R. L., Sanders J. K. M., Stoddart J. F. (2002). Dynamic
Covalent Chemistry. Angew. Chem., Int. Ed..

[20] Borre E., Stumbe J. F., Bellemin-Laponnaz S., Mauro M. (2016). Light-Powered Self-Healable
Metallosupramolecular Soft Actuators. Angew.
Chem., Int. Ed..

[21] Deng Y., Zhang Q., Feringa B. L., Tian H., Qu D.-H. (2020). Toughening
a Self-Healable Supramolecular Polymer by Ionic Cluster-Enhanced Iron-Carboxylate
Complexes. Angew. Chem., Int. Ed..

[22] Holten-Andersen N., Harrington M. J., Birkedal H., Lee B. P., Messersmith P. B., Lee K. Y. C., Waite J. H. (2011). pH-Induced Metal-Ligand Cross-Links
Inspired by Mussel Yield Self-Healing Polymer Networks with near-Covalent
Elastic Moduli. Proc. Natl. Acad. Sci. U. S.
A..

[23] Chen Y., Kushner A. M., Williams G. A., Guan Z. (2012). Multiphase Design of
Autonomic Self-Healing Thermoplastic Elastomers. Nat. Chem..

[24] Phadke A., Zhang C., Arman B., Hsu C.-C., Mashelkar R. A., Lele A. K., Tauber M. J., Arya G., Varghese S. (2012). Rapid Self-Healing
Hydrogels. Proc. Natl. Acad. Sci. U. S. A..

[25] Cordier P., Tournilhac F., Soulie-Ziakovic C., Leibler L. (2008). Self-Healing and Thermoreversible
Rubber from Supramolecular Assembly. Nature.

[26] Song Y., Liu Y., Qi T., Li G. L. (2018). Towards Dynamic, but Supertough Healable
Polymers via Biomimetic Hierarchical Hydrogen Bonding Interactions. Angew. Chem., Int. Ed..

[27] Yanagisawa Y., Nan Y., Okuro K., Aida T. (2018). Mechanically Robust, Readily Repairable
Polymers via Tailored Noncovalent Cross-Linking. Science.

[28] Nakahata M., Takashima Y., Yamaguchi H., Harada A. (2011). Redox-Responsive Self-Healing
Materials Formed from Host-Guest Polymers. Nat.
Commun..

[29] Zhang M., Xu D., Yan X., Chen J., Dong S., Zheng B., Huang F. (2012). Self-Healing
Supramolecular Gels Formed by Crown Ether Based Host-Guest
Interactions. Angew. Chem., Int. Ed..

[30] Burattini S., Greenland B. W., Hayes W., Mackay M. E., Rowan S. J., Colquhoun H. M. (2011). A Supramolecular Polymer Based on Tweezer-Type Π–π
Stacking Interactions: Molecular Design for Healability and Enhanced
Toughness. Chem. Mater..

[31] Burattini S., Greenland B. W., Merino D. H., Weng W., Seppala J., Colquhoun H. M., Hayes W., Mackay M. E., Hamley I. W., Rowan S. J. (2010). A Healable
Supramolecular Polymer Blend Based on Aromatic
Π–π Stacking and Hydrogen-Bonding Interactions. J. Am. Chem. Soc..

[32] Taylor D. L., in het Panhuis M. (2016). Self-Healing
Hydrogels. Adv.
Mater..

[33] Wang Z., An G., Zhu Y., Liu X., Chen Y., Wu H., Wang Y., Shi X., Mao C. (2019). 3D-Printable Self-Healing
and Mechanically Reinforced Hydrogels with Host-Guest Non-Covalent
Interactions Integrated into Covalently Linked Networks. Mater. Horiz..

[34] Kakuta T., Takashima Y., Nakahata M., Otsubo M., Yamaguchi H., Harada A. (2013). Preorganized Hydrogel: Self-Healing Properties of Supramolecular
Hydrogels Formed by Polymerization of Host-Guest-Monomers That Contain
Cyclodextrins and Hydrophobic Guest Groups. Adv. Mater..

[35] Zhang C., Lu X., Wang Z., Xia H. (2022). Progress in Utilizing Dynamic Bonds
to Fabricate Structurally Adaptive Self-Healing, Shape Memory, and
Liquid Crystal Polymers. Macromol. Rapid Commun..

[36] Scheutz G. M., Lessard J. J., Sims M. B., Sumerlin B. S. (2019). Adaptable Crosslinks
in Polymeric Materials: Resolving the Intersection of Thermoplastics
and Thermosets. J. Am. Chem. Soc..

[37] Luo J., Demchuk Z., Zhao X., Saito T., Tian M., Sokolov A. P., Cao P.-F. (2022). Elastic Vitrimers: Beyond Thermoplastic
and Thermoset Elastomers. Matter.

[38] Cuminet F., Caillol S., Dantras É., Leclerc É., Ladmiral V. (2021). Neighboring
Group Participation and Internal Catalysis Effects on Exchangeable
Covalent Bonds: Application to the Thriving Field of Vitrimer Chemistry. Macromolecules.

[39] Denissen W., Winne J. M., Du Prez F. E. (2016). Vitrimers:
Permanent Organic Networks
with Glass-like Fluidity. Chem. Sci..

[40] Winne J. M., Leibler L., Du Prez F. E. (2019). Dynamic
Covalent Chemistry in Polymer
Networks: A Mechanistic Perspective. Polym.
Chem..

[41] Chen X., Dam M. A., Ono K., Mal A., Shen H., Nutt S. R., Sheran K., Wudl F. (2002). A Thermally Re-Mendable
Cross-Linked Polymeric Material. Science.

[42] Gacal B., Durmaz H., Tasdelen M. A., Hizal G., Tunca U., Yagci Y., Demirel A. L. (2006). Anthracene-Maleimide-Based Diels-Alder
“Click Chemistry” as a Novel Route to Graft Copolymers. Macromolecules.

[43] Zhang L., You Z. (2021). Dynamic Oxime-Urethane
Bonds, a Versatile Unit of High Performance
Self-Healing Polymers for Diverse Applications. Chin. J. Polym. Sci..

[44] Liu W. X., Zhang C., Zhang H., Zhao N., Yu Z. X., Xu J. (2017). Oxime-Based and Catalyst-Free Dynamic
Covalent Polyurethanes. J. Am. Chem. Soc..

[45] Bapat A. P., Sumerlin B. S., Sutti A. (2020). Bulk Network Polymers with Dynamic
B–O Bonds: Healable and Reprocessable Materials. Mater. Horiz..

[46] Li L., Chen X., Jin K., Rusayyis M. B., Torkelson J. M. (2021). Arresting
Elevated-Temperature Creep and Achieving Full Cross-Link Density Recovery
in Reprocessable Polymer Networks and Network Composites via Nitroxide-Mediated
Dynamic Chemistry. Macromolecules.

[47] Yokochi H., Ohira M., Oka M., Honda S., Li X., Aoki D., Otsuka H. (2021). Topology Transformation toward Cyclic,
Figure-Eight-Shaped, and Cross-Linked Polymers Based on the Dynamic
Behavior of a Bis­(Hindered Amino)­Disulfide Linker. Macromolecules.

[48] Capelot M., Montarnal D., Tournilhac F., Leibler L. (2012). Metal-Catalyzed Transesterification
for Healing and Assembling of Thermosets. J.
Am. Chem. Soc..

[49] Snyder R. L., Fortman D. J., De Hoe G. X., Hillmyer M. A., Dichtel W. R. (2018). Reprocessable
Acid-Degradable Polycarbonate Vitrimers. Macromolecules.

[50] Self J. L., Dolinski N. D., Zayas M. S., Read de Alaniz J., Bates C. M. (2018). Bronsted-Acid-Catalyzed Exchange in Polyester Dynamic
Covalent Networks. ACS Macro Lett..

[51] Montarnal D., Capelot M., Tournilhac F., Leibler L. (2011). Silica-Like Malleable
Materials from Permanent Organic Networks. Science.

[52] Denissen W., Rivero G., Nicolaÿ R., Leibler L., Winne J. M., Du Prez F. E. (2015). Vinylogous Urethane Vitrimers. Adv. Funct. Mater..

[53] Denissen W., Droesbeke M., Nicolaÿ R., Leibler L., Winne J. M., Du Prez F. E. (2017). Chemical
Control of the Viscoelastic Properties of
Vinylogous Urethane Vitrimers. Nat. Commun..

[54] Christensen P. R., Scheuermann A. M., Loeffler K. E., Helms B. A. (2019). Closed-Loop Recycling
of Plastics Enabled by Dynamic Covalent Diketoenamine Bonds. Nat. Chem..

[55] Kovaricek P., Lehn J. M. (2012). Merging Constitutional and Motional
Covalent Dynamics
in Reversible Imine Formation and Exchange Processes. J. Am. Chem. Soc..

[56] Bhattacharya S., Phatake R. S., Nabha
Barnea S., Zerby N., Zhu J. J., Shikler R., Lemcoff N. G., Jelinek R. (2019). Fluorescent Self-Healing
Carbon Dot/Polymer Gels. ACS Nano.

[57] Kathan M., Kovaricek P., Jurissek C., Senf A., Dallmann A., Thunemann A. F., Hecht S. (2016). Control of Imine Exchange Kinetics
with Photoswitches to Modulate Self-Healing in Polysiloxane Networks
by Light Illumination. Angew. Chem., Int. Ed..

[58] Zheng P., McCarthy T. J. (2012). A Surprise from 1954: Siloxane Equilibration Is a Simple,
Robust, and Obvious Polymer Self-Healing Mechanism. J. Am. Chem. Soc..

[59] Nishimura Y., Chung J., Muradyan H., Guan Z. (2017). Silyl Ether as a Robust
and Thermally Stable Dynamic Covalent Motif for Malleable Polymer
Design. J. Am. Chem. Soc..

[60] Elling B. R., Dichtel W. R. (2020). Reprocessable Cross-Linked
Polymer Networks: Are Associative
Exchange Mechanisms Desirable?. ACS. Cent. Sci..

[61] Wang K.-Z. (2012). Lanthanides:
β-Diketonate Compounds. Encyclopedia of
Inorganic and Bioinorganic Chemistry.

[62] Mehrotra R. C. (1988). Chemistry
of Metal β-Diketonates. Pure Appl. Chem..

[63] Bray D. J., Clegg J. K., Lindoy L. F., Schilter D. (2006). Self-Assembled Metallo-Supramolecular
Systems Incorporating Beta-Dixetone Motifs as Structural Elements. Adv. Inorg. Chem..

[64] Feng J., Zhang H. (2013). Hybrid Materials Based on Lanthanide
Organic Complexes: A Review. Chem. Soc. Rev..

[65] Wanninger S., Lorenz V., Subhan A., Edelmann F. T. (2015). Metal Complexes
of Curcumin–Synthetic Strategies, Structures and Medicinal
Applications. Chem. Soc. Rev..

[66] Niu W., Zhang Z., Chen Q., Cao P.-F., Advincula R. C. (2021). Highly
Recyclable, Mechanically Isotropic and Healable 3D-Printed Elastomers
via Polyurea Vitrimers. ACS Mater. Lett..

[67] González D. M., Cruz N. B., Hernández L. A., Oyarce J., Benavente R., Manzur C. (2020). Bis-β-(Diketonates) Zn­(II) Complexes Substituted
with Thiophene: Electropolymerization, Homogeneous and Heterogeneous
Catalysis for Ring Opening Polymerization of Lactide. J. Polym. Sci..

[68] Zhang X., Vidavsky Y., Aharonovich S., Yang S. J., Buche M. R., Diesendruck C. E., Silberstein M. N. (2020). Bridging Experiments and Theory:
Isolating the Effects of Metal-Ligand Interactions on Viscoelasticity
of Reversible Polymer Networks. Soft Matter.

[69] Wright T., Tomkovic T., Hatzikiriakos S. G., Wolf M. O. (2019). Photoactivated Healable
Vitrimeric Copolymers. Macromolecules.

[70] Ma Y., Stellacci F. (2025). Structure–Property
Relationships of Elastomeric
Vinylogous Urethane Thermosets and Their Application as Closed-Loop
Recyclable Strain Sensors. Macromolecules.

[71] Lin Y., Chen Y., Yu Z., Huang Z., Lai J.-C., Tok J. B.-H., Cui Y., Bao Z. (2022). Reprocessable and Recyclable
Polymer Network Electrolytes via Incorporation of Dynamic Covalent
Bonds. Chem. Mater..

[72] Deng L., Furuta P. T., Garon S., Li J., Kavulak D., Thompson M. E., Frechet J. M. J. (2006). Living Radical
Polymerization of
Bipolar Transport Materials for Highly Efficient Light Emitting Diodes. Chem. Mater..

[73] Savchenko I., Berezhnytska A., Trunova E., Rusakova N., Fedorov Y., Grozduyk G. (2016). Poly Complexes Based Unsaturated β-Diketones
and Rare Earth Elements for Optoelectronics. Mol. Cryst. Liq. Cryst..

[74] Efstathiou S., Ma C., Coursari D., Patias G., Al-Shok L., Eissa A. M., Haddleton D. M. (2022). Functional
pH-Responsive Polymers Containing Dynamic
Enaminone Linkages for the Release of Active Organic Amines. Polym. Chem..

[75] Wang C., Zhao N., Yuan W. (2020). NIR/Thermoresponsive
Injectable Self-Healing
Hydrogels Containing Polydopamine Nanoparticles for Efficient Synergistic
Cancer Thermochemotherapy. ACS Appl. Mater.
Interfaces.

[76] Kassem H., Imbernon L., Stricker L., Jonckheere L., Du Prez F. E. (2023). Reprocessable Polyurethane Foams Using Acetoacetyl-Formed
Amides. ACS Appl. Mater. Interfaces.

[77] Ji S., Wang L., Feng S., Li L. (2024). Closed-Loop Recylcing
of Thermoset Poly­(Vinylogous Urethane) Foams. Chem. Eng. J..

[78] Abd-alhamed H., Keshe M., Alabaas R. (2024). The Chemical Organic
Reactions of
β-Diketones to Prepare Different β-Diketone Derivatives,
Their Properties and Its Applications: A Review. Results Chem..

[79] Kljun J., Turel I. (2017). β-Diketones as Scaffolds for
Anticancer Drug Design–From
Organic Building Blocks to Natural Products and Metallodrug Components. Eur. J. Inorg. Chem..

[80] Pröhl M., Schubert U. S., Weigand W., Gottschaldt M. (2016). Metal Complexes
of Curcumin and Curcumin Derivatives for Molecular Imaging and Anticancer
Therapy. Coord. Chem. Rev..

[81] Joo W.-J., Oh C.-H., Song S.-H., Kim P.-S., Han Y.-K. (2001). Photoinduced
Birefringence in Poly­(Malonic Ester) Containing *p*-Cyanoazobenzene with Photoexcitation of Cis Conformer. J. Phys. Chem. B.

[82] Banerjee S., Chakravarty A. R. (2015). Metal Complexes
of Curcumin for Cellular Imaging, Targeting,
and Photoinduced Anticancer Activity. Acc. Chem.
Res..

[83] Lessard J. J., Scheutz G. M., Sung S. H., Lantz K. A., Epps T. H., Sumerlin B. S. (2020). Block Copolymer Vitrimers. J.
Am. Chem. Soc..

[84] Chang J. Y., Kim B. J., Park C. R., Han M. J., Kim T. J., Yun H. (2001). Synthesis and Polymerization Mechanism of Bisacetoacetamides. J. Polym. Sci. A Polym. Chem..

[85] Manley J. M., Kalman M. J., Conway B. G., Ball C. C., Havens J. L., Vaidyanathan R. (2003). Early Amidation
Approach to 3-[(4-Amido)­Pyrrol-2-Yl]-2-Indolinones. J. Org. Chem..

[86] Liu J., Li J.-J. (2022). Mapping Crosslinking
Reaction-Structure-Property Relationship in
Polyether-Based Vinylogous Urethane Vitrimers. AIChE J..

[87] Taplan C., Guerre M., Bowman C. N., Du Prez F. E. (2021). Surface Modification
of (Non)-Fluorinated Vitrimers through Dynamic Transamination. Macromol. Rapid Commun..

[88] Haida P., Chirachanchai S., Abetz V. (2023). Starch-Reinforced Vinylogous Urethane
Vitrimer Composites: An Approach to Biobased, Reprocessable, and Biodegradable
Materials. ACS Sustain. Chem. Eng..

[89] Liu H., Guo L., Tao S., Huang Z., Qi H. (2021). Freely Moldable Modified
Starch as a Sustainable and Recyclable Plastic. Biomacromolecules.

[90] Masuda S., Minagawa K. (1996). Tautomers as Monomers
and Initiators. Prog. Polym. Sci..

[91] Masuda S., Sertova N., Petkov I. (1997). Photochemical
Behavior of Poly­(Ethylacryloylacetate)
and Poly­(Acryloylacetone) Films. J. Polym. Sci.
A Polym. Chem..

[92] Yamamoto M., Ando K., Inoue M., Kanoh H., Yamagami M., Wakiya T., Iida E., Taniguchi T., Kishikawa K., Kohri M. (2020). Poly-β-Ketoester
Particles
as a Versatile Scaffold for Lanthanide-Doped Colorless Magnetic Materials. ACS Appl. Mater. Interfaces.

[93] Sanchez-Sanchez A., Pomposo J. A. (2015). Efficient Synthesis
of Single-Chain Polymer Nanoparticles
via Amide Formation. J. Nanomater..

[94] Kaliyappan T., Kannan P. (2000). Co-Ordination Polymers. Prog.
Polym. Sci..

[95] Schlaad H., Krasia T., Patrickios C. S. (2001). Controlled
Synthesis of Coordination
Block Copolymers with β-Dicarbonyl Ligating Segments. Macromolecules.

[96] Krasia T., Soula R., Borner H. G., Schlaad H. (2003). Controlled Synthesis
of Homopolymers and Block Copolymers Based on 2-(Acetoacetoxy)­Ethyl
Methacrylate via RAFT Radical Polymerisation. Chem. Commun..

[97] Schlaad H., Krasia T., Antonietti M. (2004). Superhelices
of Poly­[2-(Acetoacetoxy)­Ethyl
Methacrylate]. J. Am. Chem. Soc..

[98] Demetriou M., Krasia-Christoforou T. (2008). Synthesis
and Characterization of Well-Defined Block
and Statistical Copolymers Based on Lauryl Methacrylate and 2-(Acetoacetoxy)­Ethyl
Methacrylate Using RAFT-Controlled Radical Polymerization. J. Polym. Sci. A Polym. Chem..

[99] Lessard J. J., Garcia L. F., Easterling C. P., Sims M. B., Bentz K. C., Arencibia S., Savin D. A., Sumerlin B. S. (2019). Catalyst-Free Vitrimers
from Vinyl Polymers. Macromolecules.

[100] Lessard J. J., Stewart K. A., Sumerlin B. S. (2022). Controlling Dynamics
of Associative Networks through Primary Chain Length. Macromolecules.

[101] Diodati L. E., Liu S., Rinaldi-Ramos C. M., Sumerlin B. S. (2023). Magnetic Nanoparticles Improve Flow Rate and Enable
Self-Healing in Covalent Adaptable Networks. ACS Appl. Mater. Interfaces.

[102] Benoit D., Chaplinski V., Braslau R., Hawker C. J. (1999). Development
of a Universal Alkoxyamine for “Living” Free Radical
Polymerizations. J. Am. Chem. Soc..

[103] Braddock C., Chadwick D., Lindner-López E. (2004). A Convenient
Synthesis of High-Loaded Palladium­(II) ROMP Polymers. Tetrahedron Lett..

[104] Christopherson C. J., Hackett Z. S., Sauvé E. R., Paisley N. R., Tonge C. M., Mayder D. M., Hudson Z. M. (2018). Synthesis
of Phosphorescent Iridium-Containing Acrylic Monomers and Their Room-Temperature
Polymerization by Cu(0)-RDRP. J. Polym. Sci.
A Polym. Chem..

[105] Mandal P., Banerjee S. L., Bhattacharya K., Singha N. K. (2018). A Superparamagnetic Metallopolymer Using Tailor-Made
Poly­[2-(Acetoacetoxy)­Ethyl Methacrylate] Bearing Pendant β-Keto
Ester Functionality. Eur. Polym. J..

[106] Tie J., Rong L., Xu H. (2020). An Autonomously
Healable, Highly
Stretchable and Cyclically Compressible, Wearable Hydrogel as a Multimodal
Sensor. Polym. Chem..

[107] Chen X., Ma Y., Qiao Y., Guo W., Min Y., Fan J., Shi Z. (2023). Strong and Tough Octyl
Enamine-Grafted
Polyvinyl Alcohol with Programmable Shape Deformation *via* Simple Soaking Treatment. Mater. Adv..

[108] Ma Y., Jiang X., Shi Z., Berrocal J. A., Weder C. (2023). Closed-Loop
Recycling of Vinylogous Urethane Vitrimers. Angew. Chem., Int. Ed..

[109] Liu J., Li J.-J., Luo Z.-H., Zhou Y.-N. (2023). Fast Room-temperature
Self-healing Vitrimers Enabled by Accelerated Associative Exchange
Kinetics. Chem. Eng. J..

[110] Sougrati L., Duval A., Avérous L. (2023). From Lignins
to Renewable Aromatic Vitrimers Based on Vinylogous Urethane. ChemSusChem.

[111] Liu H., Rong L., Wang B., Xie R., Sui X., Xu H., Zhang L., Zhong Y., Mao Z. (2017). Facile Fabrication
of Redox/pH Dual Stimuli Responsive Cellulose Hydrogel. Carbohydr. Polym..

[112] Qiao Y., Ma Y., Chen X., Guo W., Min Y., Fan J., Shi Z. (2023). Cellulose-Based Fluorescent Films
with Anti-Counterfeiting and UV Shielding Capabilities Enabled by
Enamine Bonds. Mater. Adv..

[113] Huang K., Xu H., Chen C., Shi F., Wang F., Li J., Hu S. (2021). A Novel Dual Crosslinked
Polysaccharide Hydrogel with Self-Healing and Stretchable Properties. Polym. Chem..

[114] Rong L., Liu H., Wang B., Mao Z., Xu H., Zhang L., Zhong Y., Feng X., Sui X. (2019). Durable Antibacterial
and Hydrophobic Cotton Fabrics Utilizing Enamine Bonds. Carbohydr. Polym..

[115] Wei X., Ge J., Gao F., Chen F., Zhang W., Zhong J., Lin C., Shen L. (2021). Bio-Based
Self-Healing
Coating Material Derived from Renewable Castor Oil and Multifunctional
Alamine. Eur. Polym. J..

[116] Zhu Y., Gao F., Shen L., Lin Y. (2020). Renewable Castor Oil
and DL-Limonene Derived Fully Bio-Based Vinylogous Urethane Vitrimers. Eur. Polym. J..

[117] Clemens R. J. (1986). Diketene. Chem.
Rev..

[118] Gómez-Bombarelli R., González-Pérez M., Pérez-Prior M. T., Manso J. A., Calle E., Casado J. (2009). Kinetic Study of the Neutral and Base Hydrolysis of
Diketene. J. Phys. Org. Chem..

[119] Mei C., Ding J., Yao B., Cheng Y., Xie Z., Geng Y., Wang L. (2007). Synthesis
and Characterization of
White-Light-Emitting Polyfluorenes Containing Orange Phosphorescent
Moieties in the Side Chain. J. Polym. Sci. A
Polym. Chem..

[120] Ma Z., Ding J., Zhang B., Mei C., Cheng Y., Xie Z., Wang L., Jing X., Wang F. (2010). Red-Emitting Polyfluorenes
Grafted with Quinoline-Based Iridium Complex: “Simple Polymeric
Chain, Unexpected High Efficiency”. Adv.
Funct. Mater..

[121] Xu Y., Guan R., Jiang J., Yang W., Zhen H., Peng J., Cao Y. (2008). Molecular Design of Efficient White-Light-Emitting
Fluorene-Based Copolymers by Mixing Singlet and Triplet Emission. J. Polym. Sci. A Polym. Chem..

[122] Jiang J., Jiang C., Yang W., Zhen H., Huang F., Cao Y. (2005). High-Efficiency Electrophosphorescent
Fluorene-Alt-Carbazole Copolymers N-Grafted with Cyclometalated Ir
Complexes. Macromolecules.

[123] Jiang J., Xu Y., Yang W., Guan R., Liu Z., Zhen H., Cao Y. (2006). High-Efficiency
White-Light-Emitting
Devices from a Single Polymer by Mixing Singlet and Triplet Emission. Adv. Mater..

[124] Zhang K., Chen Z., Zou Y., Gong S., Yang C., Qin J., Cao Y. (2009). Effective
Suppression
of Intra- and Interchain Triplet Energy Transfer to Polymer Backbone
from the Attached Phosphor for Efficient Polymeric Electrophosphorescence. Chem. Mater..

[125] Priyadarsini K. (2014). The Chemistry of Curcumin: From Extraction
to Therapeutic
Agent. Molecules.

[126] Prasad S., DuBourdieu D., Srivastava A., Kumar P., Lall R. (2021). Metal–Curcumin
Complexes in
Therapeutics: An Approach to Enhance Pharmacological Effects of Curcumin. IJMS.

[127] Tang H., Murphy C. J., Zhang B., Shen Y., Van Kirk E. A., Murdoch W. J., Radosz M. (2010). Curcumin Polymers as
Anticancer Conjugates. Biomaterials.

[128] Arshad N., Javaid M. A., Zia K. M., Hussain M. T., Arshad M. M., Tahir U. (2023). Development of Biocompatible Aqueous
Polyurethane Dispersions Using Chitosan and Curcumin to Improve Physicochemical
Properties of Textile Surfaces. Int. J. Biol.
Macromol..

[129] Zhang T., Deng Y., Zhang W., Wang G., Zhong Y., Su C., Li H. (2022). A Self-Colored Waterborne
Polyurethane Film with Natural Curcumin as a Chain Extender and Excellent
UV-Absorbing Properties. Polymer.

[130] Feng Y., Xiao K., He Y., Du B., Hong J., Yin H., Lu D., Luo F., Li Z., Li J., Tan H., Fu Q. (2021). Tough and Biodegradable
Polyurethane-Curcumin Composited Hydrogel with Antioxidant, Antibacterial
and Antitumor Properties. Mater. Sci. Eng.,
C.

[131] Zhang Q., Niu S., Wang L., Lopez J., Chen S., Cai Y., Du R., Liu Y., Lai J. C., Liu L., Li C. H., Yan X., Liu C., Tok J. B., Jia X., Bao Z. (2018). An Elastic Autonomous
Self-Healing Capacitive Sensor Based on a Dynamic Dual Crosslinked
Chemical System. Adv. Mater..

[132] Ma Y., Ren Q., Liu Z., Wang K., Zhou S., Shi Z., Yin J. (2021). Reversible
Stimuli-Responsive Luminescent Polymers
with Adaptable Mechanical Properties Based on Europium-Malonate Complex. Polymer.

[133] Tillet G., Boutevin B., Ameduri B. (2011). Chemical Reactions
of Polymer Crosslinking and Post-Crosslinking at Room and Medium Temperature. Prog. Polym. Sci..

[134] Kawaguchi S. (1986). Variety in the Coordination Modes
of β-Dicarbonyl
Compounds in Metal Complexes. Coord. Chem. Rev..

[135] Hilt G., Weske D. F. (2009). Aromatic Compounds
as Synthons for
1,3-Dicarbonyl Derivatives. Chem. Soc. Rev..

[136] Weerathaworn S., Abetz V. (2023). Tailor-Made Vinylogous
Urethane Vitrimers
Based on Binary and Ternary Block and Random Copolymers: An Approach
toward Reprocessable Materials. Macromol. Chem.
Phys..

[137] Berezhnytska O. S., Savchenko I. A., Ivakha N. B., Rogovtsov O. O., Trunova O. K., Rusakova N. V. (2020). Luminescent Properties of Heterometallic
β-Dicarbonyl Complexes and Polymers on Their Basis. J. Mol. Struct..

[138] Hoshino Y., Hagihara Y. (1999). Synthesis and Characterization
of
a Metal Coordination Polymer Consisting of Ruthenium­(III) β-Diketone
Units Linked by Butadiyne Bridges. Inorg. Chim.
Acta.

[139] Ma Y., Liu Z., Zhou S., Jiang X., Shi Z., Yin J. (2021). Aminoesterenamide Achieved by Three-Component Reaction
Heading toward
Tailoring Covalent Adaptable Network with Great Freedom. Macromol. Rapid Commun..

[140] Ma Y., Jiang X., Yin J., Weder C., Berrocal J. A., Shi Z. (2023). Chemical Upcycling
of Conventional Polyureas into Dynamic Covalent
Poly­(Aminoketoenamide)­s. Angew. Chem., Int.
Ed..

[141] Ma Y., Berrocal J. A., Jiang X., Shi Z. (2023). Fast (Re)­Processing
of Urea-Containing Polymers Enabled by Dynamic Aminoketoenamide Bonds. ACS Sustain. Chem. Eng..

[142] Liu Z., Yu C., Zhang C., Shi Z., Yin J. (2019). Revisiting
Acetoacetyl Chemistry to Build Malleable Cross-Linked Polymer Networks
via Transamidation. ACS Macro Lett..

[143] Helms B. A. (2022). Polydiketoenamines for a Circular
Plastics Economy. Acc. Chem. Res..

[144] Jadhav T., Dhokale B., Saeed Z., Hadjichristidis N., Mohamed S. (2024). Dynamic Covalent Chemistry of Enamine-ones: Exploring
Tunable Reactivity in Vitrimeric Polymers and Covalent Organic Frameworks. ChemSusChem.

[145] Stukenbroeker T., Wang W., Winne J. M., Du Prez F. E., Nicolaÿ R., Leibler L. (2017). Polydimethylsiloxane Quenchable Vitrimers. Polym. Chem..

[146] Haida P., Abetz V. (2020). Acid-Mediated Autocatalysis
in Vinylogous
Urethane Vitrimers. Macromol. Rapid Commun..

[147] Demarteau J., Epstein A. R., Christensen P. R., Abubekerov M., Wang H., Teat S. J., Seguin T. J., Chan C. W., Scown C. D., Russell T. P., Keasling J. D., Persson K. A., Helms B. A. (2022). Circularity in Mixed-Plastic Chemical
Recycling Enabled by Variable Rates of Polydiketoenamine Hydrolysis. Sci. Adv..

[148] Demarteau J., Cousineau B., Wang Z., Bose B., Cheong S., Lan G., Baral N. R., Teat S. J., Scown C. D., Keasling J. D., Helms B. A. (2023). Biorenewable and
Circular Polydiketoenamine Plastics. Nat. Sustain..

[149] Hu Z., Hu F., Deng L., Yang Y., Xie Q., Gao Z., Pan C., Jin Y., Tang J., Yu G., Zhang W. (2023). Reprocessible
Triketoenamine-Based Vitrimers with Closed-Loop Recyclability. Angew. Chem., Int. Ed..

[150] Breuillac A., Kassalias A., Nicolaÿ R. (2019). Polybutadiene
Vitrimers Based on Dioxaborolane Chemistry and Dual Networks with
Static and Dynamic Cross-Links. Macromolecules.

[151] Röttger M., Domenech T., Van Der Weegen R., Breuillac A., Nicolaÿ R., Leibler L. (2017). High-Performance Vitrimers
from Commodity Thermoplastics through Dioxaborolane Metathesis. Science.

[152] Cordes E. H., Bull H. G. (1974). Mechanism and Catalysis
for Hydrolysis
of Acetals, Ketals, and Ortho Esters. Chem.
Rev..

[153] Zhang W., Gao F., Chen X., Shen L., Chen Y., Lin Y. (2023). Recyclable, Degradable, and Fully
Bio-Based Covalent Adaptable Polymer Networks Enabled by a Dynamic
Diacetal Motif. ACS Sustain. Chem. Eng..

[154] Yu S., Wu S., Zhang C., Tang Z., Luo Y., Guo B., Zhang L. (2020). Catalyst-Free Metathesis of Cyclic Acetals and Spirocyclic
Acetal Covalent Adaptable Networks. ACS Macro
Lett..

[155] Zhang H., Wu Y., Yang J., Wang D., Yu P., Lai C. T., Shi A., Wang J., Cui S., Xiang J., Zhao N., Xu J. (2019). Superstretchable Dynamic
Polymer Networks. Adv. Mater..

[156] Zhang Z., Lei D., Zhang C., Wang Z., Jin Y., Zhang W., Liu X., Sun J. (2023). Strong and Tough Supramolecular
Covalent Adaptable Networks with Room-Temperature Closed-Loop Recyclability. Adv. Mater..

[157] Lu X., Xie P., Li X., Li T., Sun J. (2024). Acid-Cleavable
Aromatic Polymers for the Fabrication of Closed-Loop Recyclable Plastics
with High Mechanical Strength and Excellent Chemical Resistance. Angew. Chem., Int. Ed..

[158] Berezhnytska O., Savchenko I., Ivakha N., Trunova O., Rusakova N., Smola S., Rogovtsov O. (2017). Synthesis,
Characterization, and Luminescent Properties of Polymer Complexes
of Nd­(III) with β-Dicarbonyl Ligands. Nanoscale Res. Lett..

[159] Zhao J., Yuan J., Fang Z., Huang S., Chen Z., Qiu F., Lu C., Zhu J., Zhuang X. (2022). One-Dimensional Coordination
Polymers Based on Metal–Nitrogen
Linkages. Coord. Chem. Rev..

[160] Van Lijsebetten F., De Bruycker K., Van Ruymbeke E., Winne J. M., Du Prez F. E. (2022). Characterising Different
Molecular
Landscapes in Dynamic Covalent Networks. Chem.
Sci..

[161] García F., Smulders M. M. J. (2016). Dynamic Covalent Polymers. J.
Polym. Sci. A Polym. Chem..

[162] Burnworth M., Knapton D., Rowan S. J., Weder C. (2007). Metallo-Supramolecular
Polymerization: A Route to Easy-To-Process Organic/Inorganic Hybrid
Materials. J. Inorg. Organomet. Polym..

[163] Winter A., Schubert U. S. (2016). Synthesis and Characterization of
Metallo-Supramolecular Polymers. Chem. Soc.
Rev..

[164] Balkenende D. W. R., Coulibaly S., Balog S., Simon Y. C., Fiore G. L., Weder C. (2014). Mechanochemistry with Metallosupramolecular
Polymers. J. Am. Chem. Soc..

[165] Marx F., Beccard M., Ianiro A., Dodero A., Neumann L. N., Stoclet G., Weder C., Schrettl S. (2023). Structure
and Properties of Metallosupramolecular Polymers with a Nitrogen-Based
Bidentate Ligand. Macromolecules.

[166] Onori I., Formon G. J. M., Weder C., Augusto
Berrocal J. (2024). Toughening Healable Supramolecular Double Polymer Networks
Based on Hydrogen Bonding and Metal Coordination. Chem.—Eur. J..

[167] Sautaux J., Marx F., Gunkel I., Weder C., Schrettl S. (2022). Mechanically Robust Supramolecular
Polymer Co-Assemblies. Nat. Commun..

[168] Li C. H., Zuo J. L. (2020). Self-Healing Polymers
Based on Coordination
Bonds. Adv. Mater..

[169] Park H., Kang T., Kim H., Kim J.-C., Bao Z., Kang J. (2023). Toughening Self-Healing
Elastomer Crosslinked by Metal–Ligand
Coordination through Mixed Counter Anion Dynamics. Nat. Commun..

[170] Brock A. J., Clegg J. K., Li F., Lindoy L. F. (2018). Recent
Developments in the Metallo-Supramolecular Chemistry of Oligo-β-Diketonato
Ligands. Coord. Chem. Rev..

[171] Bray D. J., Clegg J. K., Lindoy L. F., Schilter D. (2006). Self-Assembled
Metallo-Supramolecular Systems Incorporating β-Diketone Motifs
as Structural Elements. Adv. Inorg. Chem..

[172] Kurajica S. (2019). A Brief Review on the Use of Chelation
Agents in Sol-Gel
Synthesis with Emphasis on β-Diketones and β-Ketoesters. Chem. Biochem. Eng. Q..

[173] Clegg J. K., Li F., Lindoy L. F. (2022). Oligo-β-Diketones
as Versatile Ligands for Use in Metallo-Supramolecular Chemistry:
Recent Progress and Perspectives. Coord. Chem.
Rev..

[174] De Gonzalo G., Alcántara A. R. (2021). Recent
Developments in the Synthesis
of β-Diketones. Pharm..

[175] Ulbricht C., Becer C. R., Winter A., Veldman D., Schubert U. S. (2008). Copolymers Containing Phosphorescent
Iridium­(III) Complexes
Obtained by Free and Controlled Radical Polymerization Techniques. Macromol. Rapid Commun..

[176] Kim E. E., Kononevich Y. N., Dyuzhikova Y. S., Ionov D. S., Khanin D. A., Nikiforova G. G., Shchegolikhina O. I., Vasil’ev V. G., Muzafarov A. M. (2022). Cross-Linked
Luminescent Polymers Based on β-Diketone-Modified Polysiloxanes
and Organoeuropiumsiloxanes. Polymers.

[177] Tsurui M., Kitagawa Y., Shoji S., Fushimi K., Hasegawa Y. (2023). Enhanced Circularly Polarized Luminescence
of Chiral
Eu­(iii) Coordination Polymers with Structural Strain. Dalton Trans..

[178] Ye M.-Y., Zhang M.-X., Xu Q.-F., Xu H., Long L.-S., Zheng L.-S. (2023). Highly Stable Eu-Coordination Polymer
Exhibiting the Highest Quantum Yield. Sci. China
Chem..

[179] Papaphilippou P., Loizou L., Popa N. C., Han A., Vekas L., Odysseos A., Krasia-Christoforou T. (2009). Superparamagnetic
Hybrid Micelles, Based on Iron Oxide Nanoparticles and Well-Defined
Diblock Copolymers Possessing β-Ketoester Functionalities. Biomacromolecules.

[180] Gohy J.-F. (2009). Metallo-Supramolecular Block Copolymer
Micelles. Coord. Chem. Rev..

[181] He J., Zhang T.-Y., Chen G. (2012). Ammonia Gas-Sensing
Characteristics
of Fluorescence-Based Poly­(2-(Acetoacetoxy)­Ethyl Methacrylate) Thin
Films. J. Colloid Interface Sci..

[182] Godlewska-Żyłkiewicz B., Zambrzycka E., Leśniewska B., Wilczewska A. Z. (2012). Separation
of Ruthenium from Environmental
Samples on Polymeric Sorbent Based on Imprinted Ru­(III)-Allyl Acetoacetate
Complex. Talanta.

[183] Marmor S., Kidane G. (1978). Preparation of a Chelating
Poly­(β-Diketone)
Resin from Poly­(Vinyl Alcohol) and Its Use in the Reversible Complexing
of Metal Ions. Polym. Bull..

[184] Panteli S., Savva I., Efstathiou M., Vekas L., Marinica O. M., Krasia-Christoforou T., Pashalidis I. (2019). β-Ketoester-Functionalized Magnetoactive Electrospun
Polymer Fibers as Eu­(III) Adsorbents. SN Appl.
Sci..

[185] Demetriou M., Krasia-Christoforou T. (2012). Well-Defined
Diblock Copolymers Possessing
Fluorescent and Metal Chelating Functionalities as Novel Macromolecular
Sensors for Amines and Metal Ions. J. Polym.
Sci. A Polym. Chem..

[186] Cao F., Yuan Z., Liu J., Ling J. (2015). Europium­(Iii) β-Diketone
Complex as Portable Luminescent Chemosensor for Naked Eye Cu2+ Detection
and Recyclable on-off-on Vapor Response. RSC
Adv..

[187] Tie J., Liu H., Lv J., Wang B., Mao Z., Zhang L., Zhong Y., Feng X., Sui X., Xu H. (2019). Multi-Responsive,
Self-Healing and Adhesive PVA Based Hydrogels Induced
by the Ultrafast Complexation of Fe3+ Ions. Soft Matter.

[188] Quan L., Tie J., Wang Y., Mao Z., Zhang L., Zhong Y., Sui X., Feng X., Xu H. (2022). Mussel-Inspired Chitosan-Based Hydrogel
Sensor with pH-Responsive
and Adjustable Adhesion, Toughness and Self-Healing Capability. Polym. Adv. Technol..

[189] Xu H., Zhao S., Yuan A., Zhao Y., Wu X., Wei Z., Lei J., Jiang L. (2023). Exploring Self-Healing and Switchable
Adhesives Based on Multi-Level Dynamic Stable Structure. Small.

[190] Spiesschaert Y., Danneels J., Van Herck N., Guerre M., Acke G., Winne J., Du Prez F. (2021). Polyaddition
Synthesis Using Alkyne Esters for the Design of Vinylogous Urethane
Vitrimers. Macromolecules.

[191] Sanchez-Sanchez A., Fulton D. A., Pomposo J. A. (2014). pH-Responsive
Single-Chain
Polymer Nanoparticles Utilising Dynamic Covalent Enamine Bonds. Chem. Commun..

[192] Capelot M., Unterlass M. M., Tournilhac F., Leibler L. (2012). Catalytic Control of the Vitrimer
Glass Transition. ACS Macro Lett..

[193] Demongeot A., Groote R., Goossens H., Hoeks T., Tournilhac F., Leibler L. (2017). Cross-Linking of Poly­(Butylene
Terephthalate)
by Reactive Extrusion Using Zn­(II) Epoxy-Vitrimer Chemistry. Macromolecules.

[194] Imbernon L., Norvez S., Leibler L. (2016). Stress Relaxation
and
Self-Adhesion of Rubbers with Exchangeable Links. Macromolecules.

[195] Van Lijsebetten F., Debsharma T., Winne J. M., Du Prez F. E. (2022). A Highly
Dynamic Covalent Polymer Network without Creep: Mission Impossible?. Angew. Chem., Int. Ed..

[196] Van Lijsebetten F., Holloway J. O., Winne J. M., Du Prez F. E. (2020). Internal
Catalysis for Dynamic Covalent Chemistry Applications and Polymer
Science. Chem. Soc. Rev..

[197] Guerre M., Taplan C., Winne J. M., Du Prez F. E. (2020). Vitrimers:
Directing Chemical Reactivity to Control Material Properties. Chem. Sci..

[198] Hamzehlou S., Ruipérez F. (2022). Computational
Study of the Transamination
Reaction in Vinylogous Acyls: Paving the Way to Design Vitrimers with
Controlled Exchange Kinetics. J. Polym. Sci..

[199] Spiesschaert Y., Taplan C., Stricker L., Guerre M., Winne J. M., Du Prez F. E. (2020). Influence of the Polymer Matrix on
the Viscoelastic Behaviour of Vitrimers. Polym.
Chem..

[200] Taplan C., Guerre M., Winne J. M., Du Prez F. E. (2020). Fast Processing
of Highly Crosslinked, Low-Viscosity Vitrimers. Mater. Horiz..

[201] Engelen S., Dolinski N. D., Chen C., Ghimire E., Lindberg C. A., Crolais A. E., Nitta N., Winne J. M., Rowan S. J., Du Prez F. E. (2024). Vinylogous Urea - Urethane Vitrimers:
Accelerating and Inhibiting Network Dynamics through Hydrogen Bonding. Angew. Chem., Int. Ed..

[202] Ishibashi J. S. A., Pierce I. C., Chang A. B., Zografos A., El-Zaatari B. M., Fang Y., Weigand S. J., Bates F. S., Kalow J. A. (2021). Mechanical and Structural Consequences
of Associative
Dynamic Cross-Linking in Acrylic Diblock Copolymers. Macromolecules.

[203] Asempour F., Laurent E., Ecochard Y., Maric M. (2024). Reprocessable
Biobased Statistical and Block Copolymer Methacrylic-Based Vitrimers
with a Shape Memory Effect. ACS Appl. Polym.
Mater..

[204] Fang H., Gao X., Zhang F., Zhou W., Qi G., Song K., Cheng S., Ding Y., Winter H. H. (2022). Triblock
Elastomeric Vitrimers: Preparation, Morphology, Rheology, and Applications. Macromolecules.

[205] Formon G. J. M., Storch S., Delplanque A. Y. -G., Bresson B., Van Zee N. J., Nicolaÿ R. (2023). Overcoming
the Tradeoff Between Processability and Mechanical Performance of
Elastomeric Vitrimers. Adv. Funct. Mater..

[206] Wang S., Li L., Liu Q., Urban M. W. (2022). Self-Healable
Acrylic-Based Covalently Adaptable Networks. Macromolecules.

[207] Gong T., Guo L., Ye J., He L., Qiu T., Li X. (2021). A Post Curing Strategy toward the
Feasible Covalent
Adaptable Networks in Polyacrylate Latex Films. J. Polym. Sci..

[208] Lessard J. J., Scheutz G. M., Hughes R. W., Sumerlin B. S. (2020). Polystyrene-Based
Vitrimers: Inexpensive and Recyclable Thermosets. ACS Appl. Polym. Mater..

[209] Tellers J., Pinalli R., Soliman M., Vachon J., Dalcanale E. (2019). Reprocessable Vinylogous Urethane
Cross-Linked Polyethylene
via Reactive Extrusion. Polym. Chem..

[210] Guerre M., Taplan C., Nicolaÿ R., Winne J. M., Du Prez F. E. (2018). Fluorinated
Vitrimer Elastomers with
a Dual Temperature Response. J. Am. Chem. Soc..

[211] Wang S., Li L., Urban M. W. (2022). Combined Reprocessability
and Self-Healing in Fluorinated Acrylic-Based Covalent Adaptable Networks
(CANs). ACS Appl. Polym. Mater..

[212] Farkhondehnia M., Maric M. (2024). Thermally Reprocessable Bio-Based
Polyhydroxyurethane Vitrimers. Polymer.

[213] Spiesschaert Y., Guerre M., De Baere I., Van Paepegem W., Winne J. M., Du Prez F. E. (2020). Dynamic Curing Agents
for Amine-Hardened
Epoxy Vitrimers with Short (Re)­Processing Times. Macromolecules.

[214] Asempour F., Marić M. (2023). Vitrification:
Versatile Method To
Modulate Properties of Myrcene-Based Rubbers. ACS Appl. Polym. Mater..

[215] Yang R., Li W., Mo R., Zhang X. (2023). Recyclable
and Degradable Vinylogous Urethane Epoxy Thermosets with Tunable Mechanical
Properties from Isosorbide and Vanillic Acid. ACS Appl. Polym. Mater..

[216] Xu H., Wang H., Zhang Y., Wu J. (2022). Vinylogous Urethane
Based Epoxy Vitrimers with Closed-Loop and Multiple Recycling Routes. Ind. Eng. Chem. Res..

[217] Zhao X.-L., Tian P.-X., Li Y.-D., Zeng J.-B. (2022). Biobased
Covalent Adaptable Networks: Towards Better Sustainability of Thermosets. Green Chem..

[218] Ma C., Liu W., Zhou X., He J., Wang Z., Wang Z. (2022). Dynamic Chemical Cross-Linking and
Mechanical Training of Bio-Based
Polyamides Fabricate Strong and Recyclable Elastomers. ACS Sustain. Chem. Eng..

[219] Hajiali F., Tajbakhsh S., Marić M. (2021). Thermally
Reprocessable Bio-Based Polymethacrylate Vitrimers and Nanocomposites. Polymer.

[220] Chen F., Gao F., Zhong J., Shen L., Lin Y. (2020). Fusion of Biobased Vinylogous Urethane Vitrimers with Distinct Mechanical
Properties. Mater. Chem. Front..

[221] Engelen S., Wróblewska A.
A., De Bruycker K., Aksakal R., Ladmiral V., Caillol S., Du Prez F. E. (2022). Sustainable
Design of Vanillin-Based Vitrimers Using Vinylogous Urethane Chemistry. Polym. Chem..

[222] Liu H., Li C., Wang B., Sui X., Wang L., Yan X., Xu H., Zhang L., Zhong Y., Mao Z. (2018). Self-Healing
and Injectable Polysaccharide Hydrogels with Tunable Mechanical Properties. Cellulose.

[223] Liu H., Yang J., Yin Y., Qi H. (2020). A Facile Strategy
to
Fabricate Polysaccharide-Based Magnetic Hydrogel Based on Enamine
Bond. Chin. J. Chem..

[224] Denissen W., De Baere I., Van Paepegem W., Leibler L., Winne J., Du Prez F. E. (2018). Vinylogous Urea
Vitrimers and Their Application in Fiber Reinforced Composites. Macromolecules.

[225] Li L., Chen X., Jin K., Torkelson J. M. (2018). Vitrimers
Designed Both To Strongly Suppress Creep and To Recover Original Cross-Link
Density after Reprocessing: Quantitative Theory and Experiments. Macromolecules.

[226] Van Lijsebetten F., De Bruycker K., Spiesschaert Y., Winne J., Du Prez F. E. (2022). Suppressing Creep
and Promoting Fast
Reprocessing of Vitrimers with Reversibly Trapped Amines. Angew. Chem., Int. Ed..

[227] Holloway J. O., Taplan C., Du Prez F. E. (2022). Combining
Vinylogous
Urethane and β-Amino Ester Chemistry for Dynamic Material Design. Polym. Chem..

[228] Van Lijsebetten F., De Bruycker K., Winne J. M., Du Prez F. E. (2022). Masked
Primary Amines for a Controlled Plastic Flow of Vitrimers. ACS Macro Lett..

[229] Mondal S., Wong A. J., Wagh M. A., Alperstein L., Sanjayan G. J., Sumerlin B. S. (2024). Creep Resistance
in Doubly Crosslinked
Dynamic Covalent Networks. Polym. Chem..

[230] Worrell B. T., McBride M. K., Lyon G. B., Cox L. M., Wang C., Mavila S., Lim C.-H., Coley H. M., Musgrave C. B., Ding Y., Bowman C. N. (2018). Bistable and Photoswitchable
States of Matter. Nat. Commun..

[231] Park J., Song H. Y., Choi S., Ahn S., Hyun K., Kim C. B. (2022). Spatiotemporal Vitrimerization of
a Thermosetting Polymer Using a Photo-Latent Catalyst for Transesterification. J. Mater. Chem. A.

[232] Reisinger D., Kaiser S., Rossegger E., Alabiso W., Rieger B., Schlögl S. (2021). Introduction
of Photolatent Bases for Locally Controlling Dynamic Exchange Reactions
in Thermo-Activated Vitrimers. Angew. Chem.,
Int. Ed..

[233] Dailing E. A., Khanal P., Epstein A. R., Demarteau J., Persson K. A., Helms B. A. (2024). Circular Polydiketoenamine Elastomers
with Exceptional Creep Resistance via Multivalent Cross-Linker Design. ACS Cent. Sci..

[234] Berne D., Cuminet F., Lemouzy S., Joly-Duhamel C., Poli R., Caillol S., Leclerc E., Ladmiral V. (2022). Catalyst-Free
Epoxy Vitrimers Based on Transesterification Internally Activated
by an α–CF _3_ Group. Macromolecules.

[235] Li Q., Ma S., Li P., Wang B., Yu Z., Feng H., Liu Y., Zhu J. (2021). Fast Reprocessing of
Acetal Covalent Adaptable Networks with High Performance Enabled by
Neighboring Group Participation. Macromolecules.

[236] Delahaye M., Tanini F., Holloway J. O., Winne J. M., Du Prez F. E. (2020). Double
Neighbouring Group Participation for Ultrafast
Exchange in Phthalate Monoester Networks. Polym.
Chem..

[237] Zheng N., Xu Y., Zhao Q., Xie T. (2021). Dynamic Covalent
Polymer Networks: A Molecular Platform for Designing Functions beyond
Chemical Recycling and Self-Healing. Chem. Rev..

[238] Liu Z., Fang Z., Zheng N., Yang K., Sun Z., Li S., Li W., Wu J., Xie T. (2023). Chemical Upcycling
of Commodity Thermoset Polyurethane Foams towards High-Performance
3D Photo-Printing Resins. Nat. Chem..

[239] Grdadolnik M., Drinčić A., Oreški A., Onder O. C., Utroša P., Pahovnik D., Žagar E. (2022). Insight into
Chemical Recycling of Flexible Polyurethane Foams by Acidolysis. ACS Sustain. Chem. Eng..

[240] Santos L. M. D., Carone C. L. P., Dullius J., Ligabue R., Einloft S. (2014). Using Different Catalysts in the
Chemical Recycling
of Waste from Flexible Polyurethane Foams. Polímeros.

[241] Sternberg J., Pilla S. (2023). Chemical Recycling of a Lignin-Based
Non-Isocyanate Polyurethane Foam. Nat. Sustain..

[242] Simón D., Borreguero A. M., De Lucas A., Rodríguez J. F. (2018). Recycling
of Polyurethanes from Laboratory to Industry, a Journey towards the
Sustainability. Waste Manage..

[243] Ou X., Zou X., Liu Q., Li L., Li S., Cui Y., Zhou Y., Yan F. (2023). Recyclable,
Fire-Resistant, Superstrong,
and Reversible Ionic Polyurea-Based Adhesives. Chem. Mater..

[244] Lai J., Xing X., Feng H., Wang Z., Xia H. (2023). Covalent Adaptive
Networks with Repairable, Reprocessable, Reconfigurable, Recyclable,
and Re-Adhesive (5R) Performance *via* Dynamic Isocyanate
Chemistry. Polym. Chem..

[245] Li Y., Wang Y., Wang S., Ye Z., Bian C., Xing X., Hong T., Jing X. (2022). Highly Tunable
and
Robust Dynamic Polymer Networks via Conjugated–Hindered Urea
Bonds. Macromolecules.

[246] Delebecq E., Pascault J.-P., Boutevin B., Ganachaud F. (2013). On the Versatility
of Urethane/Urea Bonds: Reversibility, Blocked Isocyanate, and Non-Isocyanate
Polyurethane. Chem. Rev..

[247] Bai S., Zhang K., Zhang Q., Zhu Y., Wang W., Zhang J., Li X., Zhang X., Wang R. (2023). Intrinsic
Flame Retardancy and Flexible Solid–Solid Phase Change Materials
with Self-Healing and Recyclability. ACS Appl.
Mater. Interfaces.

[248] Liu K., Wang M., Huang C., Yuan Y., Ning Y., Zhang L., Wan P. (2024). Flexible Bioinspired
Healable Antibacterial
Electronics for Intelligent Human-Machine Interaction Sensing. Adv. Sci..

[249] Shen Y., Jia Q., Xu S., Yu J., Huang C., Wang C., Lu C., Yong Q., Wang J., Chu F. (2024). Fast-Photocurable, Mechanically Robust,
and Malleable Cellulosic Bio-Thermosets Based on Hindered Urea Bond
for Multifunctional Electronics. Adv. Funct.
Mater..

[250] Michael J. P., De Koning C. B., Gravestock D., Hosken G. D., Howard A. S., Jungmann C. M., Krause R. W. M., Parsons A. S., Pelly S. C., Stanbury T. V. (1999). Enaminones:
Versatile
Intermediates for Natural Product Synthesis. Pure Appl. Chem..

[251] Soavi G., Portone F., Battegazzore D., Paravidino C., Arrigo R., Pedrini A., Pinalli R., Fina A., Dalcanale E. (2023). Phenoxy Resin-Based Vinylogous Urethane
Covalent Adaptable Networks. React. Funct. Polym..

[252] Zhang X., Cai S., Jian Z., Yang X., Wang Y., Wang Z., Lu X., Xia H. (2023). Degradable
Carbon Fiber-Reinforced Epoxy Resin Composites Based on Dynamic Benzyl
Ether Bonds. Ind. Eng. Chem. Res..

[253] Qin B., Liu S., Xu J.-F. (2023). Reversible Amidation Chemistry Enables
Closed-Loop Chemical Recycling of Carbon Fiber Reinforced Polymer
Composites to Monomers and Fibers. Angew. Chem.,
Int. Ed..

[254] Hong J., Hong Y., Jeong J., Oh D., Goh M. (2023). Robust Biobased Vitrimers and Its Application to Closed-Loop Recyclable
Carbon Fiber-Reinforced Composites. ACS Sustain.
Chem. Eng..

[255] Wang Y., Ma W., Jian Z., Zhang X., Yang X., Lu X., Wang Z., Xia H. (2023). Carbon Fiber-Reinforced
Dynamic Covalent Polymer Networks Containing Acylsemicarbazide Bonds:
Toward High-Performance Composites with Excellent Self-Healing and
Upcycling Performance. ACS Sustain. Chem. Eng..

[256] Yang R., Li W., Mo R., Zhang X. (2023). Recyclable
Biobased Dynamic Cross-Linking Epoxy Thermosets Containing Vinylogous
Urethane Bonds with Shape Memory Properties. ACS Appl. Polym. Mater..

[257] Liu Z., Ma Y. (2024). Chemical Recycling
of Step-Growth Polymers Guided by
Le Chatelier’s Principle. ACS Eng. Au.

[258] Zhang X., Zhao J., Liu K., Li G., Zhao D., Zhang Z., Wan J., Yang X., Bai R., Wang Y., Zhang W., Yan X. (2022). Weldable and Closed-Loop
Recyclable Monolithic Dynamic Covalent Polymer Aerogels. Natl. Sci. Rev..

[259] Liu Y., Huang Y., Li C., Si G., Chen M. (2024). Highly Elastic
and Degradable Vitrimeric Elastomers Using Polycondensation. Chin. Chem. Lett..

[260] Liu J., Shi Y., Li J.-J., Luo Z.-H., Zhou Y.-N. (2023). Closed-Loop
Recyclable Vinylogous Carbamothioate-Based Covalent Adaptable Networks. Macromolecules.

[261] Thiyagarajan S., Maaskant-Reilink E., Ewing T. A., Julsing M. K., Van Haveren J. (2021). Back-to-Monomer
Recycling of Polycondensation Polymers:
Opportunities for Chemicals and Enzymes. RSC
Adv..

[262] Guo X., Gao F., Chen F., Zhong J., Shen L., Lin C., Lin Y. (2021). Dynamic Enamine-One Bond Based Vitrimer via Amino-Yne
Click Reaction. ACS Macro Lett..

[263] Chen F., Gao F., Guo X., Shen L., Lin Y. (2022). Tuning the Dynamics
of Enamine-One-Based Vitrimers through Substituent
Modulation of Secondary Amine Substrates. Macromolecules.

[264] Fu X., Qin A., Tang B. Z. (2023). X-yne Click Polymerization. Aggregate.

[265] Chen X., Bai T., Hu R., Song B., Lu L., Ling J., Qin A., Tang B. Z. (2020). Aroylacetylene-Based
Amino-Yne Click Polymerization toward Nitrogen-Containing Polymers. Macromolecules.

[266] Zupanc A., Kotnik T., Štanfel U., Brodnik Žugelj H., Kristl A., Ručigaj A., Matoh L., Pahovnik D., Grošelj U., Opatz T., Požgan F., Štefane B., Žagar E., Svete J. (2019). Chemical Recycling of Polyenaminones
by Transamination Reaction via Amino–Enaminone Polymerisation/Depolymerisation. Eur. Polym. J..

[267] Gorsche C., Schnoell C., Koch T., Moszner N., Liska R. (2018). Debonding on Demand with Highly Cross-Linked
Photopolymers: A Combination
of Network Regulation and Thermally Induced Gas Formation. Macromolecules.

[268] Liu Z., Ma Y., Xia C., Ren Y., Gao J., Xiang Y., Shi S. (2024). Sustainable, Recyclable
and Biodegradable
Castor Oil-Derived Elastomers Enabled by Dynamic Acetoacetyl Formed
Amides. Chem. Eng. J..

[269] Dömling A., Wang W., Wang K. (2012). Chemistry
and Biology
Of Multicomponent Reactions. Chem. Rev..

[270] Liu Z., Ma Y. (2025). Recyclable Dynamic
Covalent Networks Derived from Isocyanate
Chemistry: The Critical Role of Electronic and Steric Effects in Reversibility. ChemSusChem.

[271] He C., Christensen P. R., Seguin T. J., Dailing E. A., Wood B. M., Walde R. K., Persson K. A., Russell T. P., Helms B. A. (2020). Conformational Entropy as a Means to Control the Behavior
of Poly­(Diketoenamine) Vitrimers In and Out of Equilibrium. Angew. Chem., Int. Ed..

[272] Epstein A. R., Demarteau J., Helms B. A., Persson K. A. (2023). Variable
Amine Spacing Determines Depolymerization Rate in Polydiketoenamines. J. Am. Chem. Soc..

[273] Vora N., Christensen P. R., Demarteau J., Baral N. R., Keasling J. D., Helms B. A., Scown C. D. (2021). Leveling
the Cost and Carbon Footprint of Circular Polymers That Are Chemically
Recycled to Monomer. Sci. Adv..

[274] Neidhart E. K., Hua M., Peng Z., Kearney L. T., Bhat V., Vashahi F., Alexanian E. J., Sheiko S. S., Wang C., Helms B. A., Leibfarth F. A. (2023). C–H
Functionalization of Polyolefins to Access Reprocessable Polyolefin
Thermosets. J. Am. Chem. Soc..

[275] Feng H., Wang S., Lim J. Y. C., Li B., Rusli W., Liu F., Hadjichristidis N., Li Z., Zhu J. (2024). Catalyst-Free
α-Acetyl Cinnamate/Acetoacetate
Exchange to Enable High Creep-Resistant Vitrimers. Angew. Chem., Int. Ed..

[276] Ma Y., Zheng C., Slor G., Özkan M., Gubelmann O. J., Stellacci F. (2024). Reaction of
β-Ketoester and
1,3-Diol to Access Chemically Recyclable and Mechanically Robust Poly­(vinyl
alcohol) Thermosets through Incorporation of β-(1,3-dioxane)­ester. Angew. Chem., Int. Ed..

[277] Dei T., Morino K., Sudo A., Endo T. (2011). Construction of Reversible
Hydration–Dehydration System by a Model Compound and a Novel
Polymer Bearing Vicinal Tricarbonyl Structure. J. Polym. Sci. A Polym. Chem..

[278] Morino K., Sudo A., Endo T. (2012). Reversible
Fixation
and Release of Alcohols by a Polymer Bearing Vicinal Tricarbonyl Moieties
and Its Application to Synthesis and Reversible Cross-Linking–De-Cross-Linking
System of a Networked Polymer. Macromolecules.

[279] Yonekawa M., Furusho Y., Takata T., Endo T. (2014). Reversible
Crosslinking and Decrosslinking of Polymers Containing Alcohol Moiety
Using an Acyclic Bifunctional Vicinal Triketone. J. Polym. Sci. A Polym. Chem..

[280] Yuki T., Yonekawa M., Matsumoto K., Tomita I., Endo T. (2016). Construction of Reversible Crosslinking–Decrosslinking
System Consisting of a Polymer Bearing Vicinal Tricarbonyl Structure
and Poly­(Ethylene Glycol). Polym. Bull..

[281] Yuki T., Yonekawa M., Matsumoto K., Sei Y., Tomita I., Endo T. (2016). Hemithioketal Formation of Vicinal
Tricarbonyl Compound with Thiols and Their Recovery. Tetrahedron.

[282] Yuki T., Yonekawa M., Furusho Y., Sei Y., Tomita I., Endo T. (2016). Reversible Capture and Release of
Aromatic Amines by Vicinal Tricarbonyl Compound. Tetrahedron.

[283] Endo T., Yonekawa M., Sudo A. (2021). Synthesis of Polymers
Containing Vicinal Tricarbonyl Moiety and Construction of Reversible
Crosslinking–Decrosslinking Polymer System. Polym. Int..

[284] Sims M. B., Lessard J. J., Bai L., Sumerlin B. S. (2018). Functional
Diversification of Polymethacrylates by Dynamic β-Ketoester
Modification. Macromolecules.

[285] Li L., Rong L., Xu Z., Wang B., Feng X., Mao Z., Xu H., Yuan J., Liu S., Sui X. (2020). Cellulosic
Sponges with pH Responsive Wettability for Efficient Oil-Water Separation. Carbohydr. Polym..

[286] Rong L., Liu H., Wang B., Mao Z., Xu H., Zhang L., Zhong Y., Yuan J., Sui X. (2018). Enamine Approach
for Versatile and Reversible Functionalization on Cellulose Related
Porous Sponges. ACS Sustain. Chem. Eng..

[287] Liu H., Sui X., Xu H., Zhang L., Zhong Y., Mao Z. (2016). Self-Healing
Polysaccharide Hydrogel Based on Dynamic Covalent Enamine
Bonds. Macromol. Mater. Eng..

[288] Maiti B., Ruidas B., De P. (2015). Dynamic Covalent
Cross-Linked
Polymer Gels through the Reaction between Side-Chain β-Keto
Ester and Primary Amine Groups. React. Funct.
Polym..

[289] Jang S., Hernandez Alvarez E.
I., Chen C., Jing B. B., Shen C., Braun P. V., Schleife A., Schroeder C. M., Evans C. M. (2023). Control of Lithium Salt Partitioning,
Coordination, and Solvation in Vitrimer Electrolytes. Chem. Mater..

[290] Wang Y., Gao M., Li S., Liu J., Feng A., Zhang L. (2022). Recyclable, Self-Healable and Reshape
Vitrified Poly-Dimethylsiloxane Composite Filled with Renewable Cellulose
Nanocrystal. Polymer.

[291] Bai L., Zheng J. (2020). Robust, Reprocessable
and Shape-Memory Vinylogous Urethane
Vitrimer Composites Enhanced by Sacrificial and Self-Catalysis Zn­(II)–Ligand
Bonds. Compos. Sci. Technol..

[292] Liu Z., Zhang C., Shi Z., Yin J., Tian M. (2018). Tailoring
Vinylogous Urethane Chemistry for the Cross-Linked Polybutadiene:
Wide Freedom Design, Multiple Recycling Methods, Good Shape Memory
Behavior. Polymer.

[293] Chen F., Cheng Q., Gao F., Zhong J., Shen L., Lin C., Lin Y. (2021). The Effect
of Latent
Plasticity on the Shape Recovery of a Shape Memory Vitrimer. Eur. Polym. J..

[294] Xiao Y., Rong L., Wang B., Mao Z., Xu H., Zhong Y., Zhang L., Sui X. (2018). A Light-Weight
and
High-Efficacy Antibacterial Nanocellulose-Based Sponge via Covalent
Immobilization of Gentamicin. Carbohydr. Polym..

[295] Shen Z., Wu Y., Qiu S., Deng H., Hou R., Zhu Y. (2020). UV-Thermal Dual-Cured
Polymers with Degradable and
Anti-Bacterial Function. Prog. Org. Coat..

[296] Rong L., Shen X., Wang B., Mao Z., Feng X., Sui X. (2020). Antibacterial Thyme Oil-Loaded Organo-Hydrogels
Utilizing Cellulose Acetoacetate as Reactive Polymer Emulsifier. Int. J. Biol. Macromol..

[297] Zhang Y., Yuan L., Guan Q., Liang G., Gu A. (2017). Developing Self-Healable and Antibacterial
Polyacrylate Coatings
with High Mechanical Strength through Crosslinking by Multi-Amine
Hyperbranched Polysiloxane via Dynamic Vinylogous Urethane. J. Mater. Chem. A.

[298] Markwart J. C., Battig A., Urbaniak T., Haag K., Koschek K., Schartel B., Wurm F. R. (2020). Intrinsic
Flame
Retardant Phosphonate-Based Vitrimers as a Recyclable Alternative
for Commodity Polymers in Composite Materials. Polym. Chem..

[299] Stewart K. A., DeLellis D. P., Lessard J. J., Rynk J. F., Sumerlin B. S. (2023). Dynamic
Ablative Networks: Shapeable Heat-Shielding
Materials. ACS Appl. Mater. Interfaces.

[300] Wang F., Huang K., Xu Z., Shi F., Chen C. (2022). Self-Healable
Nanocellulose Composite Hydrogels Combining Multiple
Dynamic Bonds for Drug Delivery. Int. J. Biol.
Macromol..

[301] Jiang X., Yang X., Yang B., Zhang L., Lu A. (2021). Highly Self-Healable
and Injectable Cellulose Hydrogels via Rapid
Hydrazone Linkage for Drug Delivery and 3D Cell Culture. Carbohydr. Polym..

[302] Tran C. H., Pham L. T. T., Jang H. B., Kim S. A., Kim I. (2021). Effect of α-, β-, γ-, and δ-Dicarbonyl Complexing
Agents on the Double Metal Cyanide-Catalyzed Ring-Opening Polymerization
of Propylene Oxide. Catal. Today.

[303] Ivakha N. B., Savchenko I. O., Berezhnytska O. S., Rusakova N. V., Trunova O. K. (2020). Ytterbium Metal
Polymers as Precursors
of Luminescent Materials Emitting in the near Infrared Region. Appl. Nanosci..

[304] Savchenko I., Berezhnytska O., Fedorov Y., Smola S., Trunova O. (2018). Luminescent Properties
of New Polymer Metal Complexes
Based β-Diketones and REE. Mol. Cryst.
Liq. Cryst..

[305] Berezhnytska O., Savchenko I., Rohovtsov O., Smola S., Fedorov Y., Trunova O. (2021). Luminescent Properties
of Complexes and Polymers of Sm (III). Opt.
Mater..

[306] Berezhnytska O. S., Savchenko I. O., Ivakha N. B., Trunova O. K., Smola S. S., Zheleznova L. I. (2018). Near Infrared Electroluminescence
Polymeric Systems Containing β-Diketones and Lanthanides as
Emitters for Organic Light-Emitting Diodes. Mol. Cryst. Liq. Cryst..

[307] Irina S., Oleksandra B., Olena T., Yaroslav F., Sergiy S., Nataliya R. (2019). Monomer and
Metallopolymer Compounds
of Tb­(III) as Precursors for OLEDs. Appl. Nanosci..

[308] Kurpik G., Walczak A., Markiewicz G., Harrowfield J., Stefankiewicz A. R. (2023). Enhanced Catalytic Performance Derived
from Coordination-Driven Structural Switching between Homometallic
Complexes and Heterometallic Polymeric Materials. Nanoscale.

